# Common cortical areas have different neural mechanisms for covert and overt visual pursuits

**DOI:** 10.1038/s41598-021-93259-9

**Published:** 2021-07-06

**Authors:** Ken-ichi Morishige, Nobuo Hiroe, Masa-aki Sato, Mitsuo Kawato

**Affiliations:** 1 Department of Intelligent Robotics, Toyama Prefectural University, 5180 Kurokawa, Imizu, Toyama 939-0398 Japan; 2 Neural Information Analysis Laboratories, Advanced Telecommunications Research Institute International, 2-2-2 Hikaridai, Keihanna Science City, Kyoto 619-0288 Japan; 3 Brain Information Communication Research Laboratory Group, Advanced Telecommunications Research Institute International, 2-2-2 Hikaridai, Keihanna Science City, Kyoto 619-0288 Japan

**Keywords:** Attention, Smooth pursuit

## Abstract

Although humans can direct their attention to visual targets with or without eye movements, it remains unclear how different brain mechanisms control visual attention and eye movements together and/or separately. Here, we measured MEG and fMRI data during covert/overt visual pursuit tasks and estimated cortical currents using our previously developed extra-dipole, hierarchical Bayesian method. Then, we predicted the time series of target positions and velocities from the estimated cortical currents of each task using a sparse machine-learning algorithm. The predicted target positions/velocities had high temporal correlations with actual visual target kinetics. Additionally, we investigated the generalization ability of predictive models among three conditions: control, covert, and overt pursuit tasks. When training and testing data were the same tasks, the largest reconstructed accuracies were overt, followed by covert and control, in that order. When training and testing data were selected from different tasks, accuracies were in reverse order. These results are well explained by the assumption that predictive models consist of combinations of three computational brain functions: visual information-processing, maintenance of attention, and eye-movement control. Our results indicate that separate subsets of neurons in the same cortical regions control visual attention and eye movements differently.

## Introduction

Although humans can direct their attention to visual targets with and without eye movements, it remains unclear how different brain mechanisms control visual attention and eye movements together and/or separately^1–3^. Previous neurophysiological studies in monkeys and brain imaging studies in humans have repeatedly shown that multiple cortical regions (frontal eye field (FEF), supplementary eye field, lateral intraparietal cortex, and so on), which are related to saccade eye movements, contribute also to visual attention^4–8^. Thompson et al. investigated the link between FEF activity and covert spatial attention by recording from FEF visual and saccade-related neurons in monkeys performing covert visual search tasks without eye movements. They reported that there exist different neural populations for saccades and visual selection in FEF^9–13^. Covert and overt attention rely on shared cortical regions, but different neural mechanisms.

Ohlendorf et al. investigated effects of dissociating visual attention and gaze directions during smooth pursuit eye movements using functional magnetic resonance imaging (fMRI)^14^. They found that covert and overt pursuit activated the cortical oculomotor network similarly, indicating that covert and overt pursuit are processed by similar neural networks. Furthermore, activations of overt pursuit tasks were stronger than those of covert pursuit tasks.

Lovejoy and colleagues investigated the spatial allocation of attention during smooth pursuit using a letter discrimination task^15^. Their results demonstrated that performance on the discrimination task was best at the tracked target location during pursuit eye movements, and symmetrically decreased ahead or behind target locations, regardless of speed. Watamaniuk and colleagues also investigated attentional allocation across a pursuit object, and their results suggest that attention is flexibly allocated during pursuit, but performance is limited by crowding and set size^16^. These behavioral results suggested that smooth pursuit and covert attention are related, but controlled differently.

Matsushima and Tanaka examined single-neuron activities in the lateral PFC when monkeys covertly tracked one of identical moving objects without eye movements^17^. They found that the majority of neurons in the lateral PFC modulated their activities depending mostly on target location. Because they did not show results of the comparison of neural activities between covert and overt object tracking, the role of attention during overt and covert pursuit remains unknown. Thus, this remaining issue is of major importance, whether there exist similar representations of the attentive target trajectory between covert and overt pursuit at the neurophysiological level.

Although there are some important differences between saccades and smooth pursuit eye movements (e.g., latencies of pursuit and saccades tend to be different), both types of eye movements are controlled by largely overlapping neural networks at the neurophysiological level^18,19^, and the two types of eye movements have similar relationships with covert attention^15,16^. Since these cortical regions are expected to provide visual-target information for controlling both visual attention and eye movements, the time series of the target information (e.g., target positions and velocities) must be represented in brain regions as necessary visual-target information for attentional control.

A neuronal-level approach is suitable for investigating from a single cell with high-temporal resolution. However, this method is limited in that it cannot simultaneously investigate brain activities of multiple cortical regions. Although the fMRI method can measure whole brain activities, acquiring high-resolution temporal data is quite difficult because of its measurement principles. Additionally, measured voxel values are not directly derived from neural responses, nor do they reflect physical entities of neuronal activities. Magnetoencephalography (MEG) is one candidate for the most promising, non-invasive brain measurement method during covert/overt visual pursuit tasks. However, eye movements induce large magnetic artifacts that contaminate the magnetic field derived from brain activities, thus complicating investigations of brain activities from MEG data with eye movements.

We simultaneously estimated not only cortical currents, but also multiple extra-brain source currents from contaminated MEG data. Even though measured MEG data were contaminated by multiple artifacts such as eye movements and heartbeats, our proposed method separated effects of artifacts and estimated cortical currents of the whole brain (extra-dipole method)^20^. Then, our sparse linear regression (SLiR) method can automatically select, in a data-driven manner, truly important cortical currents for visual attention and eye-movement control from estimated cortical currents in multiple cortical regions. Furthermore, it can predict the time series of visual-target information from selected current sources^21^. Combining the extra-dipole method and SLiR, we quantitatively predicted the time series of target information from brain regions related to visual attention and eye-movement control. We can objectively investigate what kind of time series of visual-target information is represented in these cortical regions.

If the same set of neurons are activated during both attention and eye movements, the same cortical currents must be estimated from MEG signals, because cortical currents mainly reflect neural activities. If cortical currents are common to attention and eye-movement control, the predictive model using cortical currents during attention can predict target motion during eye movements, and vice versa. If attention and eye-movement control share the same set of neurons, predictive models have some generalization abilities for both attention and eye movements. In contrast, if the two functions are controlled by different subsets of neurons in the same cortical region, cortical currents should not be able to predict target temporal information equally for both visual attention and eye movements. This paper examines the above theoretical assumptions regarding generalization abilities from one experimental condition to the other.

The main purpose of this study is to illuminate the relationship between mechanisms that govern maintenance of attention on a moving object and mechanisms that govern maintenance of fixation on a moving object, by investigating generalization ability of machine-learning-based predictive models from MEG signals to the target motion among the three experimental conditions: control, covert pursuit, and overt pursuit tasks. These trained models are expected to reflect three computational brain functions: visual information-processing, maintenance of attention, and eye-movement control. These functions are computationally different. The control task condition (only watching the fixation point) contains only the function of visual information-processing, and the covert condition contains visual information-processing and maintenance of attention, and the overt condition contains all the three functions (Table 1). If these three functions are represented by the same neuronal populations, predictive models should generalize well among all three conditions. In contrast, if neural mechanisms share no common neural populations under the three conditions, predictive models probably have no generalization ability across the three conditions.

**Table 1:** Relationship between tasks and neural functions. A circle indicates that a neural function is required for a task condition.

Task type	Function		
	Visual information-processing	Maintenance of Attention	Eye-movement control
Control	○	–	–
Covert	○	○	–
Overt	○	○	○

With respect to visual information-processing if there is a common function involved in all three conditions, the trained model using control task data should depend solely on a single function of visual information-processing. Thus, the trained model is expected to have some generalization ability, not only for control task data, but also for covert and overt task data. Additionally, the trained model using covert task data must also have the function of attentional control. The trained model, using overt task data, must have the function of attentional control, too. So, it is expected to have some generalization ability from covert to overt task data. When it comes to the reverse-direction generalization ability, prediction is more complicated, as follows. If cortical currents estimated in overt and covert task conditions mainly represent functions of motor control and attentional control, respectively, we predict that the determination coefficients as indices for generalization ability should be low under conditions from overt to covert, and from overt to control, as well as from covert to control tasks.

We found that the above predictions were supported by our data and analyses; thus, we suggest that the same set of neurons in a cortical region governs the three experimental conditions, but at the same time, the three conditions also involve some additional subsets of neurons in the same cortical region. More concretely, we conclude that in this set of neurons, the function of attentional control is common to both covert and overt conditions. However, the function of eye-movement control exists only for the overt condition and utilizes distinct neural populations. These results indicate that the different subpopulations in the same cortical regions encode visual, attentional, and eye movement-related processes in covert and overt pursuit tasks.

Another main finding is that smaller cortical currents were estimated in the precentral and parietal cortexes during visual attention tasks than during eye movement tasks, indicating that different subsets of neurons in the same cortical region exist for covert and overt visual pursuit tasks. These results demonstrate that the MEG and machine learning-based modeling approach that tests the generalization ability of the models was able to detect signatures of different active subpopulations in the same cortical region.

## Results

### Cortical current estimates for covert/overt visual pursuit tasks

**Figure 1:**
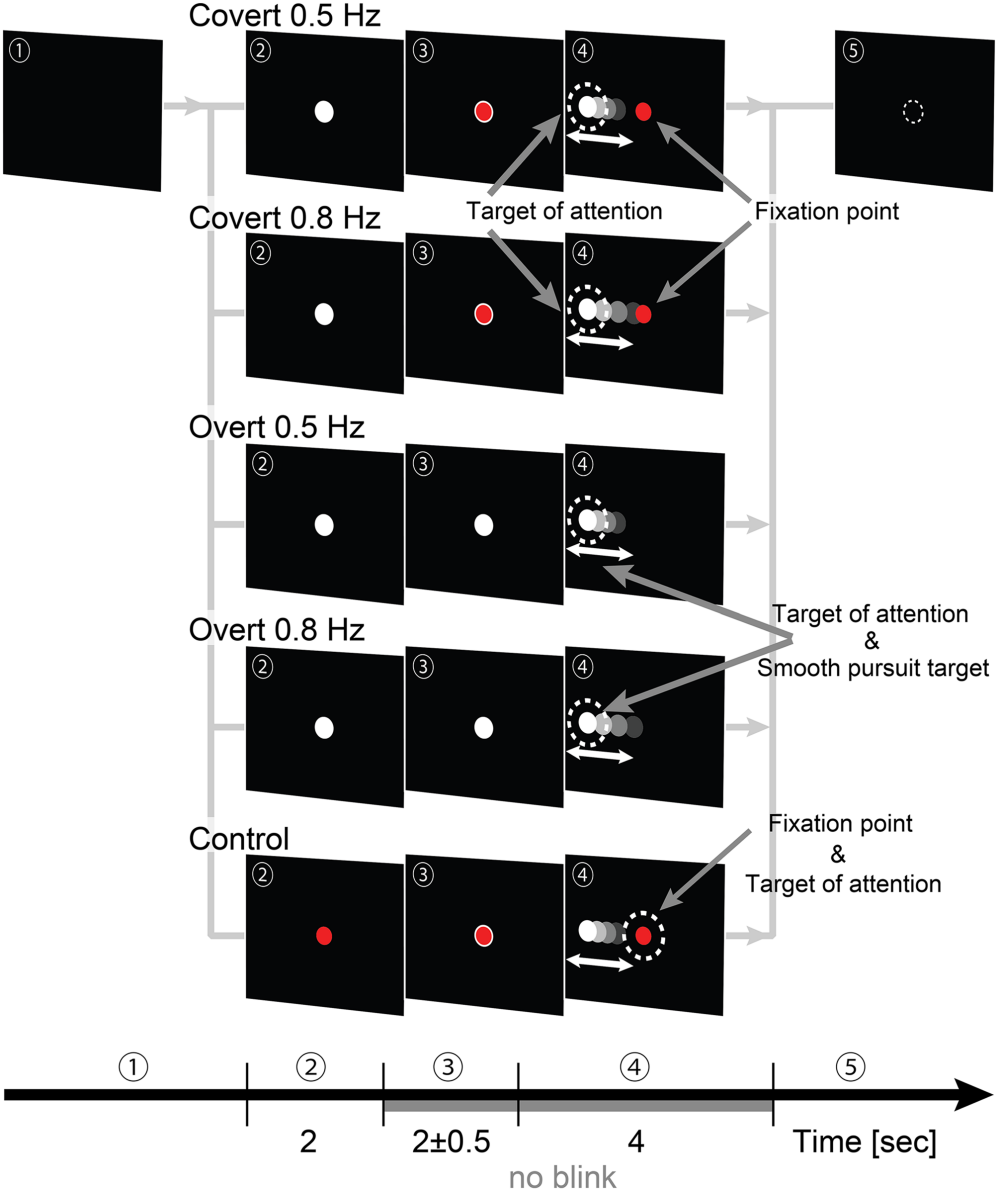
Experimental protocol. Target motions were sinusoidal. Their frequencies were 0.5 or 0.8 Hz, and their peak velocities were 20°/s. Peak amplitudes of 0.5 and 0.8 Hz target motions were 6.37° and 3.98°, respectively. Diameters of white and red-filled targets were 0.44° and 0.35°, respectively.

We asked participants to pursue a periodically moving visual target of attention covertly or overtly (see Fig. 1 and “Methods”). (1) Participants pressed a start button when they were ready. (2) Either a white or red-filled target was presented in the center of the monitor immediately after pressing the button for 2 s. If the white target appeared, participants performed covert/overt pursuit tasks, but if the red-filled target appeared, participants performed the control task. (3) After that, a white-bordered red target or white-filled target was presented in the center of the monitor for 2 s more with a uniform, random jitter ± 0.5 s. This white-bordered red target represented two targets overlap: fixation point (red-filled target) and target of attention (white-filled target). The white-filled target represented only the target of attention. (4) Then, the target of attention started to move in a horizontal direction. The participants began to pursue the target of attention covertly or overtly. (5) The fixation point and the target of attention disappeared after 4 s of movement, and participants then took a short rest. These processes were termed “trials.” One task consisted of 100 repetitions of a trial. Each participant performed 500 trials (100 trials × 5 tasks = 500 trials).

**Figure 2:**
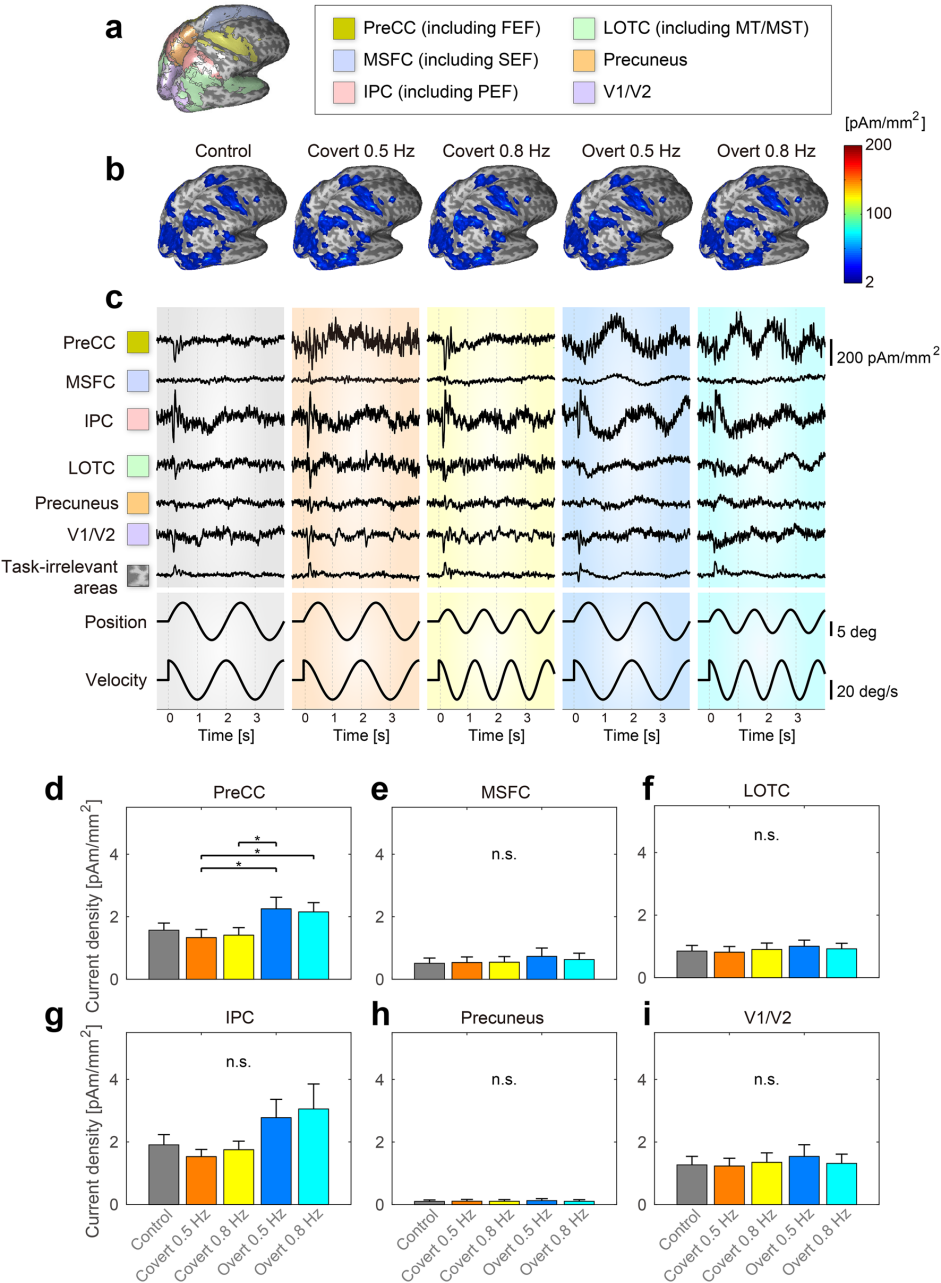
(**a**) Cortical regions of PreCC, MSFC, LOTC, IPC, Precuneus, and early visual area V1/V2. (**b**) Spatial–temporal characteristics of estimated cortical currents (an example of a typical participant). (**c**) Examples of the time series of estimated cortical currents. Starting from the left, we showed the results of the following conditions: control, covert 0.5 Hz, covert 0.8 Hz, overt 0.5 Hz, and overt 0.8 Hz. (**d**–**i**) Comparisons of current densities among five different tasks: (**d**–**i**) show PreCC, MSFC, LOTC, IPC, Precuneus, and V1/V2 results, respectively. * indicates a significant difference of multiple comparisons among five tasks at difference level 5%.

We estimated cortical currents for each participant and task using an extra-dipole method. We also calculated trial-averaged values from estimated current densities and plotted absolute and maximum values on the cortical surface model (Fig. 2b) (the results of a typical participant). The precentral cortex (PreCC), the medial superior frontal cortex (MSFC), the lateral occipito-temporal cortex (LOTC), the intraparietal cortex (IPC), the precuneus, and early visual areas (V1/V2) shared large current intensities among our participants. From previous studies, these cortical areas were the same as the expected activation areas for the smooth pursuit and visual attention tasks. We also searched maximum current densities across all dipoles on the cortical surface for each subject and then calculated mean values and standard deviations for all participants. The values of covert 0.5 Hz, covert 0.8 Hz, overt 0.5 Hz, overt 0.8 Hz, and control were respectively 131.62 ± 86.94, 132.74 ± 77.26, 319.23 ± 301.86, 347.80 ± 518.62, 149.65 ± 97.02 pAm/mm^2^. In previous studies based on electrophysiological methods^22,23^, estimated current densities were roughly in the range of 25–250 pAm/mm^2^. Current densities of pigs and guinea pigs were 400 and 800 pAm/mm^2^, respectively^24^. Calculated values in this study are also within the range of these values.

To investigate temporal changes in estimated cortical current densities, we calculated trial-averaged values of the time series of estimated current densities of all dipoles, and we plotted the time course of peak values for each cortical region (Fig. 2c). Visual evoked responses were observed around 0.1 s from the onset as a positive or negative deflection in several traces. After that time, the time series of some estimated currents were correlated with the target positions and/or velocities. In task-irrelevant areas, cortical current densities were smaller and dissimilar in comparison to those in task-relevant areas.

We calculated regional mean current intensities and investigated whether significant differences exist between the following five conditions of visual pursuit tasks: control, covert 0.5 Hz, covert 0.8 Hz, overt 0.5 Hz, and overt 0.8 Hz. Results of a randomized block design one-way ANOVA identified a significant difference in PreCC (F(4, 20) = 5.88, p = 0.0394). Post-hoc analysis using Tukey’s HSD tests revealed significantly higher current intensities under overt conditions than under covert conditions (PreCC: [covert 0.5 Hz]–[overt 0.5 Hz] p = 0.0118, [covert 0.5 Hz]–[overt 0.8 Hz] p = 0.0267, [covert 0.8 Hz]–[overt 0.5 Hz] p = 0.0233, Fig. 2d). There were no significant differences in MSFC, IPC, LOTC, Precuneus and V1/V2 (MSFC: F(4, 20) = 3.92, p = 0.0833; IPC: F(4, 20) = 1.19, p = 0.4157; LOTC: F(4, 20) = 1.07, p = 0.4570; Precuneus: F(4, 20) = 1.80, p = 0.2653; V1/V2: F(4, 20) = 1.36, p = 0.3663, Fig. 2e–i). These results showed that amplitudes of cortical current densities during smooth pursuit tasks were larger than those during covert pursuit tasks, mainly in the PreCC.

### Prediction of target trajectories from estimated cortical currents during control and covert/overt visual pursuit tasks

**Figure 3:**
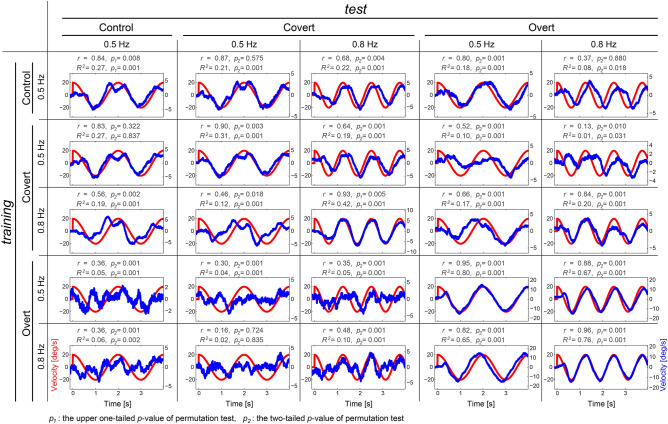
Trial-averaged values of predicted target velocities from cortical currents (example of a typical participant). Blue and red lines indicate predicted and true values, respectively.

If estimated cortical currents contain some visual-target information for control, visual attention and eye movements, we must predict the time series of target positions and velocities from estimated cortical currents. Therefore, we examined whether such predictions of target trajectories were possible from estimated single-trial cortical currents using the SLiR method (Fig. 3). When task types of training and test datasets were identical, we divided all trials into ten datasets and conducted training and tests using tenfold cross-validation (Supplementary Information 1). 58 of 60 predicted target positions and velocities (= [5 identical task combinations] × [6 participants] × [2 kinetics (position or velocity)]) had positive high correlations, and their temporal characteristics were very consistent with the true values (correlation coefficients: r = 0.82–0.98; See Tables S1, S3, and Figures S3–S14 in Supplementary Information 2 and 4), although two of these correlations did not reach significance (permutation test, [Covert 0.5 Hz training]–[Covert 0.5 Hz test] of Subj.6 (position): p = 0.402, [Control training]–[Control test] of Subj.6 (position): p = 0.584; See Figure S13 in Supplementary Information 4). Additionally, when using different tasks for training and test data, correlation coefficients between the two datasets were also large, and the true and predicted profiles had similar characteristics (correlation coefficients: r = 0.16–0.93; See Tables S1 and S3 in Supplementary Information 2). 194 of all 240 correlations (= [20 different task combinations] × [6 participants] × [2 kinetics]) reached significant levels (permutation test, BH corrected p < 0.05; See Supplementary Information 3 and Supplementary Figures S3–S14).

The determination coefficients in 58 of 60 identical task combinations and 210 of 240 different task combinations were significant (permutation test, Benjamini–Hochberg [BH] corrected p < 0.05; See Supplementary Information 3 and Supplementary Figures S3–S14). In contrast to the correlation coefficients, the determination coefficients depended on the task types for the training and test datasets. Especially when using overt task combinations as training data and covert test data and vice versa, determination coefficients tended to be small. This suggests that our obtained model has adequate generalization ability for characteristics of temporal profiles, but less for predicting their amplitudes (determination coefficients: R^2^ = − 0.09–0.92, Tables S2 and S4 in Supplementary Information 2).

**Figure 4:**
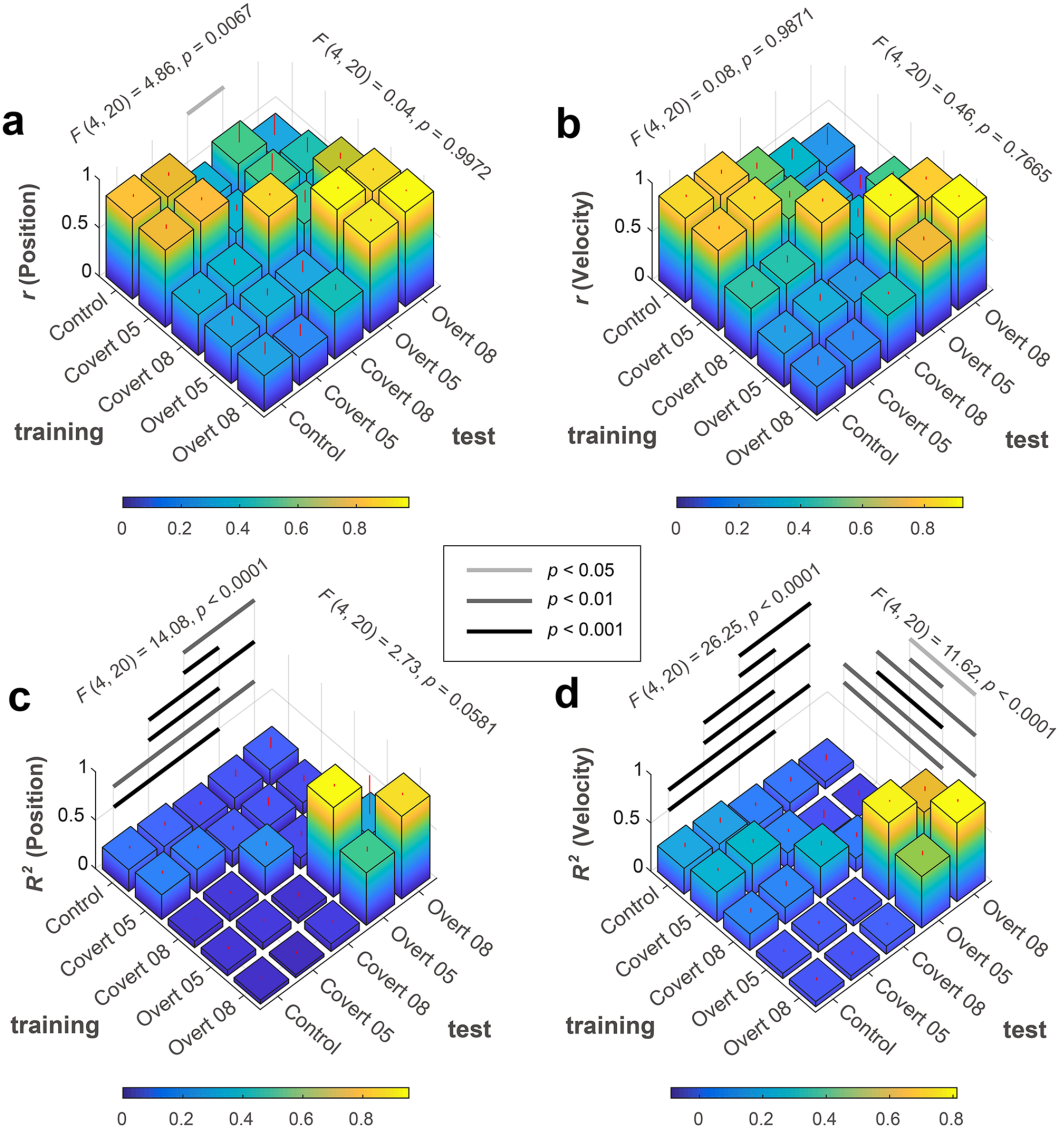
Randomized block design two-way ANOVA with Tukey’s multiple comparison test results of coefficients among task types for training and test datasets. (**a**) Correlation coefficients between target and reconstructed positions. (**b**) Correlation coefficients between target and reconstructed velocities. (**c**) Determination coefficients between target and reconstructed positions. (**d**) Determination coefficients between target and reconstructed velocities.

We conducted a randomized block design two-way ANOVA for the correlation and determination coefficients by considering the following two within-factors for the training-test condition (training or test) and the task condition (control, covert 0.5 Hz, covert 0.8 Hz, overt 0.5 Hz, or overt 0.8 Hz) (Fig. 4). Results of the randomized block design two-way ANOVA indicated no significant main effect of training and test ([main effect of training, position]: F(4, 20) = 0.04, p = 0.9972; [main effect of training, velocity]: F(4, 20) = 0.46, p = 0.7665; [main effect of test, velocity]: F(4, 20) = 0.08, p = 0.9871), except one combination ([main effect of test, position]: F(4, 20) = 4.86, p = 0.0067), but there were significant interactions ([position]: F(16, 80) = 8.70, p < 0.0001; [velocity]: F(16, 80) = 20.86, p < 0.0001). Simple main effect tests and Tukey’s multiple comparison post-hoc tests showed significant larger correlation coefficients under overt/overt or covert/covert conditions as training and test combinations (Supplementary Information 6). These results indicate that correlation coefficients did not depend much on training and test data, but they were significantly larger than the other conditions when the experimental conditions of training and test data types were the same. In contrast, for determination coefficients, randomized block design two-way ANOVA results indicated significant or marginally significant main effects of training and test and interactions ([position]; a main effect of training: F(4, 20) = 2.73, p = 0.0581; a main effect of test: F(4, 20) = 14.08, p < 0.0001; interaction between training and test: F(16, 80) = 6.56, p < 0.0001; [velocity]; a main effect of training: F(4, 20) = 11.62, p < 0.0001; a main effect of test: F(4, 20) = 26.25, p < 0.0001; interaction between training and test: F(16, 80) = 36.00, p < 0.0001). There was a significant difference based on the experimental conditions for the training and test datasets.

**Figure 5:**
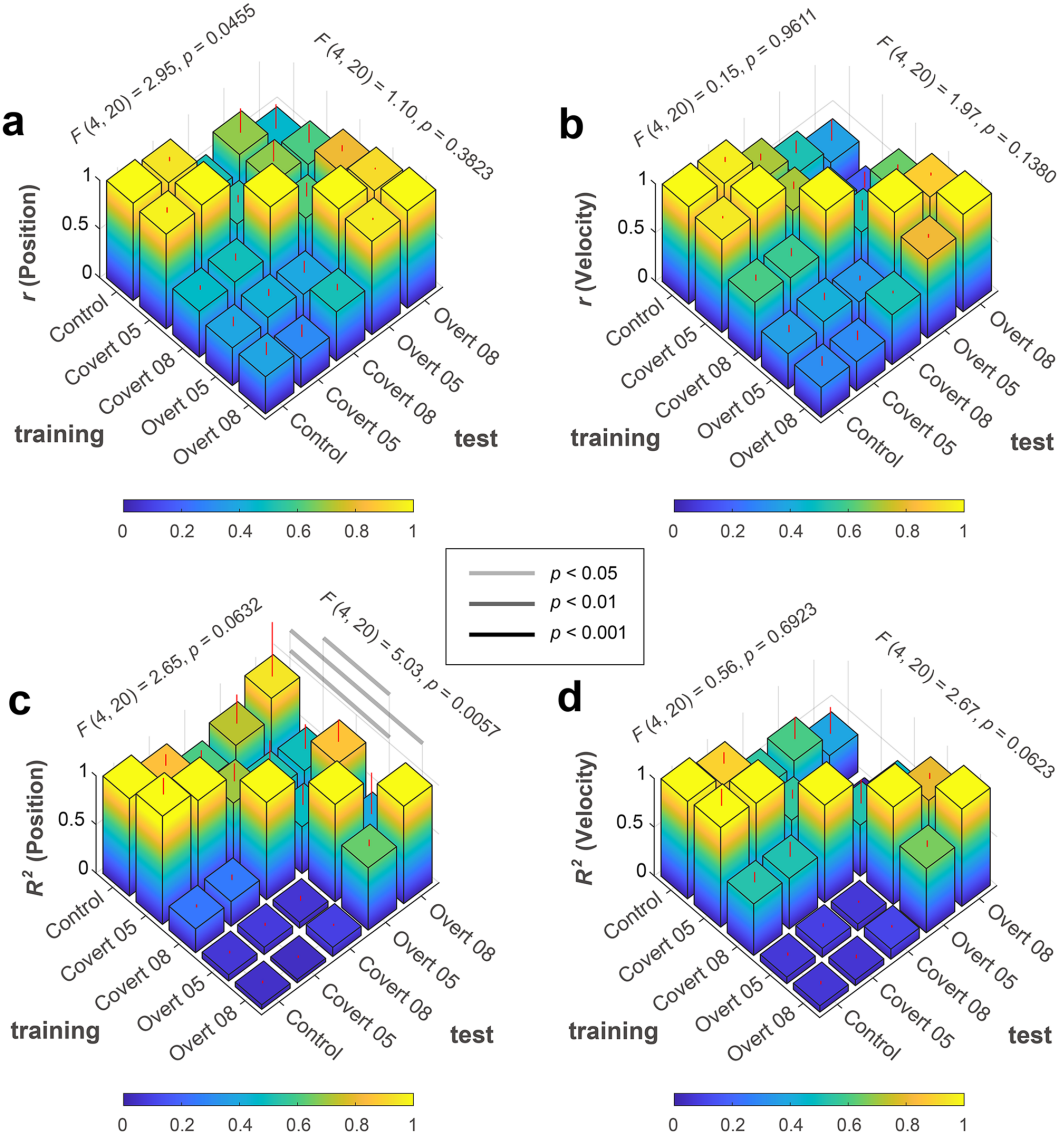
Randomized block design two-way ANOVA with Tukey’s multiple comparison test results of normalized coefficients among task types for training and test datasets. (**a**) Normalized correlation coefficients between target and reconstructed positions. (**b**) Normalized correlation coefficients between target and reconstructed velocities. (**c**) Normalized determination coefficients between target and reconstructed positions. (**d**) Normalized determination coefficients between target and reconstructed velocities.

To evaluate generalization ability among the five tasks, we calculated the normalized coefficients of correlation and determination. First, we divided each score by the value that was calculated when the task types of training and test data were the same. Second, we calculated the mean value and standard deviation among participants for each training-test data combination. Then, we compared them among task types for training and test datasets using a randomized block design two-way ANOVA (Fig. 5; Supplementary Information 7). Summarized information is shown in Table 2.

**Table 2:** Summary of generalization abilities among tasks.

	Test				
	Control	Covert		Overt	
	0.5 Hz	0.5 Hz	0.8 Hz	0.5 Hz	0.8 Hz
**Training**					
Control					
0.5 Hz	○	○	△	○	○
	0.24 ± 0.07	0.22 ± 0.10	0.14 ± 0.06	0.14 ± 0.13	0.12 ± 0.21
	1.00 ± 0.00	0.87 ± 0.27	0.58 ± 0.24	0.67 ± 0.62	0.66 ± 1.10
Covert					
0.5 Hz	○	○	○	△	×
	0.28 ± 0.06	0.29 ± 0.08	0.17 ± 0.07	0.09 ± 0.20	0.02 ± 0.27
	1.07 ± 0.40	1.00 ± 0.00	0.63 ± 0.35	0.24 ± 0.90	-0.06 ± 1.41
0.8 Hz	△	△	○	△	○
	0.12 ± 0.08	0.12 ± 0.10	0.30 ± 0.10	0.13 ± 0.11	0.19 ± 0.10
	0.39 ± 0.23	0.39 ± 0.30	1.00 ± 0.00	0.50 ± 0.53	0.66 ± 0.44
Overt					
0.5 Hz	×	×	×	○	○
	0.06 ± 0.04	0.07 ± 0.05	0.05 ± 0.07	0.86 ± 0.07	0.50 ± 0.68
	0.07 ± 0.05	0.08 ± 0.06	0.06 ± 0.08	1.00 ± 0.00	0.60 ± 0.74
0.8 Hz	×	×	×	○	○
	0.05 ± 0.05	0.04 ± 0.05	0.08 ± 0.03	0.54 ± 0.10	0.83 ± 0.05
	0.05 ± 0.06	0.05 ± 0.06	0.10 ± 0.04	0.65 ± 0.15	1.00 ± 0.00

The upper field of each cell indicates a comprehensive evaluation. Middle fields indicate the mean and standard deviation calculated from original determination coefficients for the reconstructed position and velocity data. Lower fields indicate those calculated from normalized determination coefficients. We chose each symbol in the upper field based on normalized coefficients of determination shown in the lower fields. Circle, triangle, and cross represent high- (≥ 0.6), middle- (≥ 0.2), low-generalization abilities (< 0.2), respectively. The statistical analysis and data about normalized determination coefficients are shown in Supplementary Information 7.

When control condition data were used for training, generalization ability showed good performance across all test conditions. When covert condition data were used for training, predictive models had some generalization ability for test data under control and overt conditions. When using overt condition data for training, normalized determination coefficients were maximal when training and test conditions were the same. However, the predictive model from the overt condition had poor generalization ability for other conditions (Table 2; Fig. 5c,d).

### Common dipoles selected by SLiR for covert/overt visual pursuit tasks

**Figure 6:**
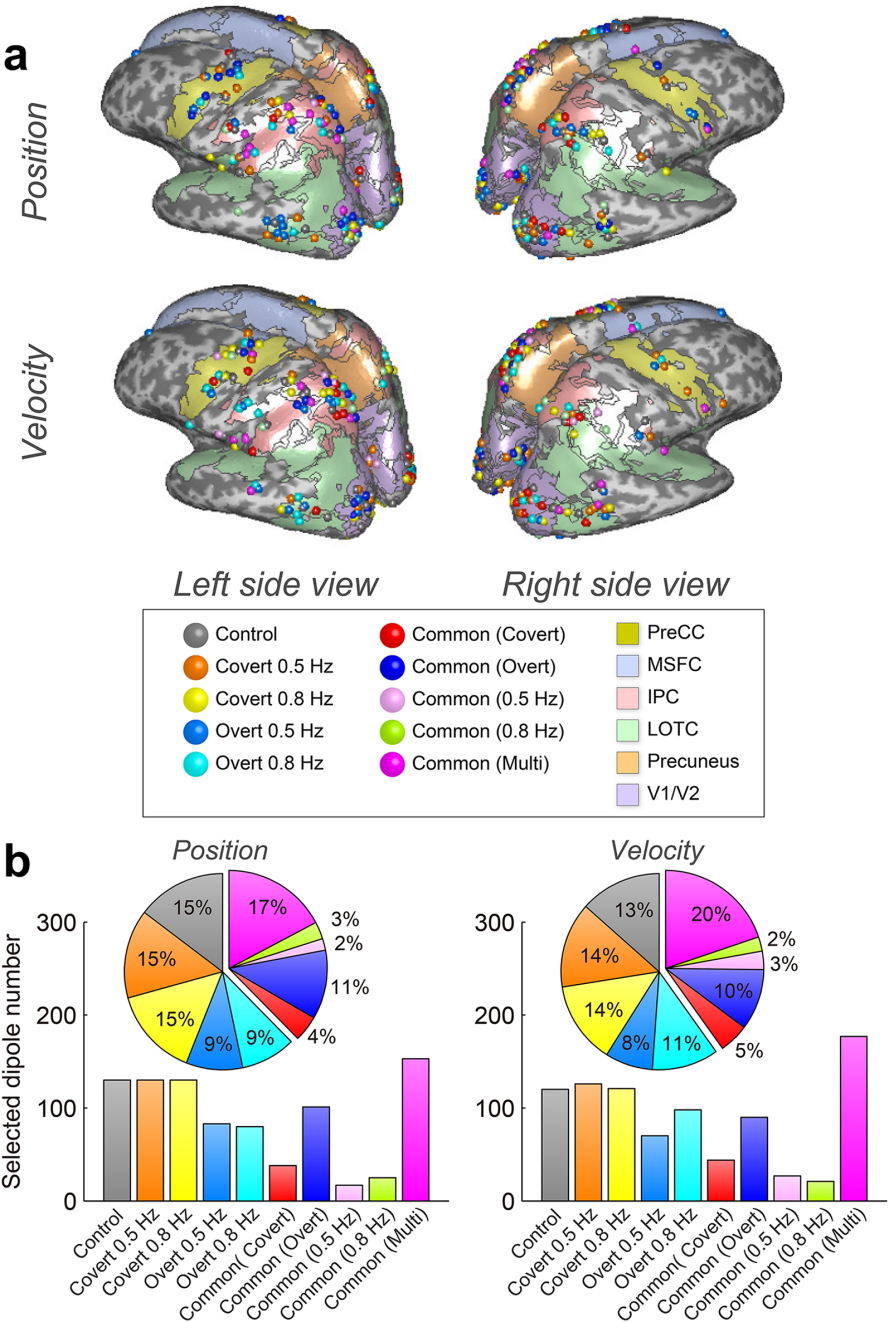
(**a**) SLiR–selected dipole locations on inflated cortical surface maps. (**b**) Rate of selected dipole numbers for tasks. The exclusively selected dipoles for control, covert 0.5 Hz, covert 0.8 Hz, overt 0.5 Hz, and overt 0.8 Hz are shown in gray, orange, yellow, blue, and cyan, respectively. The commonly selected dipoles for covert (0.5 and 0.8 Hz), overt (0.5 and 0.8 Hz), 0.5 Hz (covert and overt), and 0.8 Hz (covert and overt) are shown in red, blue-purple, pink, and green, respectively. The commonly selected dipoles by more than three tasks are shown in magenta. The same color format is used in pie charts and bar graphs in (**b**). Numbers of cortical dipole currents were 1987.17 ± 2057.03 (participant mean ± SD), and these dipole currents were used for SLiR inputs as explanatory variables. Input dimensions of control, covert 0.5 Hz, covert 0.8 Hz, overt 0.5 Hz, and overt 0.8 Hz were reduced to 55.17 ± 15.15, 62.47 ± 18.77, 55.53 ± 18.54, 64.50 ± 19.79, and 68.57 ± 20.15 [position] and 56.90 ± 16.67, 64.93 ± 17.79, 56.50 ± 14.44, 61.07 ± 25.72, and 69.07 ± 19.02 [velocity]. Cortical regions of PreCC, MSFC, LOTC, IPC, precuneus, and V1/V2 had current dipole numbers of 141.33 ± 127.20, 59.33 ± 64.35, 335.50 ± 364.52, 314.83 ± 283.26, 26.50 ± 34.80, and 529.50 ± 583.36, respectively. SLiR sparseness narrowed input dimensions to [position]: 4.83 ± 1.60, 2.50 ± 3.99, 7.00 ± 4.34, 10.50 ± 7.12, 0.67 ± 0.82, and 16.33 ± 10.80, [velocity]: 6.33 ± 3.22, 3.33 ± 4.80, 8.33 ± 7.55, 9.83 ± 6.91, 0.33 ± 0.52, and 13.17 ± 7.22, respectively.

Predicted target trajectories had some significant correlations with true target kinetics when using different as well as identical tasks for training and test data as prediction models. This result indicates that common dipoles among different tasks are expected to be selected as explanatory variables by SLiR. We categorized common and differentially selected dipoles among five tasks: control, covert 0.5 Hz, covert 0.8 Hz, overt 0.5 Hz, and overt 0.8 Hz. We used different colors for each category and plotted the dipoles on inflated cortical surface maps (Fig. 6a; Supplementary Figs. S15 and S16). Dipoles selected by SLiR, which were widely distributed in multiple cortical regions, were mainly distributed in PreCC, IPC, LOTC, and V1/V2. We investigated the rate of shared or unshared current dipole numbers and plotted pie charts (Fig. 6b). When predicting target positions and velocities, 44% and 45% of SLiR-selected dipoles were shared by two or more types of tasks. Almost one third of the dipoles were commonly used among different tasks.

## Discussion

This study examined the relationship between mechanisms that govern maintenance of attention at a moving object and mechanisms that govern maintenance of fixation on a moving object, by investigating generalization ability of machine-learning-based predictive models from MEG signals to the target motion among three experimental conditions: control, covert pursuit, and overt pursuit tasks. When task types of training and test data were the same, we divided all single-trial data into training and test datasets and predicted time series of target positions and velocities from estimated cortical currents. Predicted visual-target kinetics were highly correlated with actual target kinetics. These results identified neural representations of time series of target positions and velocities for each task. When task types of training and test data differed, prediction models could not adequately reproduce amplitudes of actual visual-target kinetics, although shared cortical dipoles were selected by SLiR. A major finding is that during visual attention tasks, smaller currents were estimated in cortical regions of precentral and parietal cortexes than during eye movement tasks. These results indicate that common cortical areas control visual attention and eye movements, even though the two functions employ different subsets of neurons in the same cortical region.

To evaluate the relationship between time series of target kinetics and reconstructed data, we employed two similarity criteria: correlation coefficients and determination coefficient (Goodness-of-fit). The first has the advantage of evaluating shape similarity of two temporal profiles, but it is not sensitive to amplitude differences. In contrast, the second criterion makes it possible to simultaneously evaluate both temporal shape and amplitudes. In this paper, the correlation coefficient represents only the temporal shape relationship between target kinematics and reconstructed data, while the determination coefficient represents not only shape, but also spatial amplitude components.

In order to pursue moving visual targets covertly or overtly, we assume that the following three brain functions mainly contribute to achieve covert/overt visual pursuit: visual information-processing and maintenance of attention, and/or eye-movement control. We hypothesize that the control task is achieved only by the visual information-processing function, the covert pursuit task is achieved with both visual information-processing and attention functions, and the overt pursuit task is achieved using visual information-processing, attention, as well as eye-movement control functions (Table 1).

If the trained model using overt task data largely contained computational components of visual information-processing and/or attentional functions, the model should reconstruct the time series of target kinetics from test data during control and covert conditions fairly well (generalization capability of overt condition). However, determination coefficients tend to be small, e.g., from 0.03 to 0.08 (Table 2; Supplementary Information Tables S2 and S4), and much smaller than the maximum value obtained for the overt test condition (Fig. 5). In other words, under the overt conditions, the trained model possessed only small components of visual information-processing or covert attention.

Using overt-condition data, the trained model showed poor generalization ability for covert-condition data (Fig. 5; Table 2; Supplementary Information Tables S2 and S4). This implies that neuronal mechanisms for attention and eye-movement control do not overlap perfectly. The trained model using overt data also showed very poor generalization ability for control data. This suggests that neuronal mechanisms for eye-movement control and visual information-processing are different. Additionally, it implies that the neuronal mechanism for visual information-processing contributes little to reconstruction of target kinetics in the model trained under the overt condition.

When covert 0.8 Hz was used for both training and test data, R^2^ ranged between 0.29 and 0.32 ([pos] R^2^ = 0.29 ± 0.09; [vel] R^2^ = 0.32 ± 0.11), and was intermediate. When covert 0.8 Hz was used for training and control data were used for test data, R^2^ varied between 0.07 and 0.17 ([pos] R^2^ = 0.07 ± 0.01; [vel] R^2^ = 0.17 ± 0.10), and was rather small. Given that this factor of acquired R^2^ values was small enough, we conclude that visual information did not contribute much to reconstruct the model trained under covert 0.8 Hz. Thus, neuronal mechanisms for visual information-processing and attention do not overlap perfectly.

The trained model using covert 0.5 Hz data can predict target kinetics of covert test data with R^2^ values between 0.24 and 0.33 ([pos] R^2^ = 0.24 ± 0.05; [vel] R^2^ = 0.33 ± 0.09). In contrast to covert 0.8 Hz, generalization ability of covert 0.5 Hz to control data varies between 0.26 and 0.31 ([pos] R^2^ = 0.26 ± 0.06; [vel] R^2^ = 0.31 ± 0.06), and was intermediate and almost the same as R^2^ values with the covert/covert reconstruction (see also Fig. 5). These results indicate that the trained model using covert 0.5 Hz data predicts target kinetics depending largely on visual information-processing (Supplementary Information Tables S2 and S4).

For that reason, we could not draw a clear conclusion about overlapping neural populations between covert and control conditions, and this question is still open to future study. However, we would like to emphasize that the main point of our paper, i.e., that neural populations for attention and eye-movement control are different, is not affected by the above-mentioned complication.

When we examined reverse-direction generalizations with a control-data-trained model to test data obtained under covert/overt conditions, determination coefficients ranged from 0.07 to 0.24. These are rather low, but are similar to determination coefficients (0.22–0.26) when control data were used for both training and testing (Figure 5). This indicates that the control-trained model has some generalization ability for covert/overt task data (Table 2; Supplementary Information Tables S2 and S4). Although these scores were not so small compared with those of generalization ability from covert/overt to control data, this is not surprising for the following reason. Because neural populations for visual information-processing are also necessary for covert/overt conditions, it is reasonable to expect that we can partially reconstruct time series of target kinetics using a control-trained model for covert/overt data.

Although we removed eye movement artifacts from overt condition MEG data using the extra-dipole method, there is still a possibility that our proposed denoising method is imperfect. If so, residual MEG artifacts in the estimated cortical currents could directly reflect eye movement in the form of electrooculography, and contribute significantly to artifactitious predictions. If residual eye-movement artifacts account for most target kinetics, there should exist no temporal lag between estimated cortical currents and target motion because eye movement artifacts occurred at the same time as actual target motion. On the other hand, if brain activities account for most target kinetics, there exists a relatively large time lag between them because brain activities were expected to precede target motion in order to compensate for the time delay caused by the control pathway for smooth pursuit eye movements. To investigate this point, by varying the time lag between cortical currents and target motion, we reconstructed target kinetics from cortical currents under overt conditions using the SLiR method, and calculated correlation coefficients between target motion and reconstructed data (Supplementary Information 8; Figure S17). Correlation coefficients were small near zero-time lag and large at the time when cortical currents preceded target motion. Especially, correlation coefficients were maximal with time lags of − 50 to − 250 ms (Figure S17). Previously, Churchland and colleagues showed that the temporal delay was about 60 ms in monkeys^25^, and Miura et al. and Shibata et al. showed that time delays were more than 70 and 200 ms in humans, respectively^26,27^. Our estimated time lag in the prediction was within the range of these previous results. We note that the above result was obtained only for a single participant, and we need to continue to analyze data for the remaining participants in the future.

Under covert conditions, smaller currents were estimated in the regions of the precentral and parietal cortexes in comparison with those under overt conditions. The precentral cortex contains a supplementary eye field, and the presence or absence of eye movements influenced the intensities of cortical currents. The parietal cortex contributes to bottom-up attention, but top-down neural mechanisms can suppress bottom-up attentional capture^17,28–30^. In overt conditions, presentation of the pursuit target itself automatically induces attention and affects bottom-up attention. In the covert condition, in contrast, participants shifted their attention to the target without moving their eyes, while gazing at the fixation point. Top-down attention is required to achieve this function. One reasonable way to explain this ability is that the prefrontal cortex (dlPFC) and FEF contribute to top-down attention (mechanisms that ignore the moving target in a positive manner), thus achieving the function of covert attention^30^.

Acs and Greenlee measured fMRI data during saccade, smooth pursuit eye movements under covert and overt conditions and investigated functional connectivity of multiple brain regions using a dynamic causal modeling (DCM) method. Their results suggested that connectivity from V1 to hMT+ contributed to covert/overt attention^31^. In our results, SLiR selected dipoles in the V1 and MT+ brain regions as common cortical areas for covert/overt pursuit. Cortical regions selected by the sparse machine learning algorithm SLiR were consistent with results of previous studies. However, other cortical area dipoles were also selected and they may contribute to covert/overt pursuit. We need to further scrutinize effective connectivity in using fMRI/MEG/estimated cortical current data^32,33^.

Different types of dipoles were identified in the same cortical region by SLiR. It is clear that each identified dipole represents each specific subpopulation of neurons such as visual cells, controlling-attention cells, and specifically active eye-movement cells. However, the plausibility of such an interpretation depends on the spatial resolution of cortical current estimation in an MEG inverse problem. We employed a cortical surface representation with 20,004 dipole sources, with an average distance of 1.88 ± 0.08 mm (subject mean and standard deviation) between nearest neighbors. One dipole represents many thousands of neurons and often approximates more complex patterns of neural electrical sources, thus indicating that each identified dipole may represent mixed subpopulations of neurons. We need careful reexamination when our results extend to neurophysiological data. Although it is plausible that the same cells in FEF and superior colliculus may be activated differently for control, covert, and overt tasks, our non-invasive method has not reached sufficient sophistication to differentiate eye movements and attention control mechanisms at neuronal levels with the required spatial resolution. This is also a limitation of our machine-learning based approach, and we need to continue to improve the current estimation method from MEG data.

At first, we expected to detect frequency features of microsaccades from the observed EOG data, but we could not detect them. In order to measure microsaccades, some additional measurement equipment is necessary. Unfortunately, we cannot discuss the relationship between visual attention and microsaccades in this paper. We hope to investigate this in the future.

## Methods

### Participants

Eight males (24–31 year old) with normal or corrected-to-normal visual acuity participated in both the MEG and fMRI experiments, which were approved by the Ethics and Safety Committee of Advanced Telecommunications Research Institute International (ATR). All experiments were performed in accordance with approved guidelines and regulations. Written informed consent was obtained from each participant prior to the experiment.

### fMRI/MRI data collections

We obtained structural and functional magnetic resonance images using a 1.5-T MR scanner (MAGNEX ECLIPSE 1.5 T Power Drive 250; Shimadzu-Marconi, Japan) with the following sequence parameters: repetition time (TR) = 3000 ms, echo time (TE) = 49 ms, field of view = 192 mm, matrix size: 64 × 64 × 30, voxel dimensions = 3 × 3 × 5 mm. T1-weighted anatomical images were acquired with 1 × 1 × 1 mm resolution with a gradient echo sequence (TR = 20 ms; TE = 2.26 ms; matrix size = 256 × 256 × 191). To minimize head movements, participants used a bite bar that was rigidly set on a head coil.

### MEG data collection

We used a whole-head, 208-channel system (MEG vision-PQ1400RM; Yokogawa Electric Co., Japan) for MEG recordings at a sampling frequency of 1000-Hz. An electrooculogram (EOG) and an electrocardiogram (ECG) were simultaneously recorded. Before the MEG experiment, the face and head shape of each participant were scanned using a hand-held laser scanner and a stylus marker (FastSCAN Cobra; Polhemus, USA) for subsequent co-registration of the MEG sensor position with the structural MRI. Electromagnetic calibration of the coil positions was conducted using the procedure of Toda et al.^34^ before and after each MEG recording session by passing alternating currents to the coils.

### Task design and procedure

To investigate the relationship between visual attention and eye-movement control, participants performed the following five tasks (Fig. 1):

- Covert pursuit task 0.5 Hz (Covert 0.5 Hz)
  Participants were instructed not to move their eyes and to covertly pursue the moving target. Target motion was sinusoidal with a frequency of 0.5 Hz.
- Covert pursuit task 0.8 Hz (Covert 0.8 Hz)
  This task was the same as in (a), but the frequency of target motion was 0.8 Hz.
- Overt pursuit task 0.5 Hz (Overt 0.5 Hz)
  Participants overtly pursued a smoothly moving target, which periodically moved in the horizontal plane. Target motion was sinusoidal, and the frequency was 0.5 Hz. The target motion was the same as in (a).
- Overt pursuit task 0.8 Hz (Overt 0.8 Hz)
  The task was the same as (a), but the frequency of target motion was 0.8 Hz. The target motion was the same as that in (b).
- Control task
  Participants were asked not to move their eyes, to avoid direct attention to the moving target, and to just gaze at the fixation point. The target motion was the same as in (a).

We used an identically shaped visual target for covert/overt visual pursuit and control tasks. In covert pursuit and control tasks, not only the visual target motion, but also the fixation points were identical. The same visual stimuli were used; only instructions for participants differed.

In the main experiment, no visual feedback was presented after individual trials. Even if participants mistakenly moved their eyes during covert/control tasks, they continued to do the experiment, so as not to abort any trial or experiment, in order to concentrate on the tasks.

### fMRI data analysis

fMRI images were preprocessed and analyzed using SPM8 (The Wellcome Department of Cognitive Neurology, UK). Statistical analyses were performed using general linear models (GLMs) for each participant. We created a boxcar regressor for four conditions (covert 0.5 Hz, covert 0.8 Hz, overt 0.5 Hz, and overt 0.8 Hz). The boxcar function of the regressor spanned the covert/overt visual pursuit or the rest intervals. The regressor was convoluted with a canonical hemodynamic response function. A parameter was estimated for the regressor using the least-square method. *t*-statistics were used for comparisons between estimated parameters to yield a *t*-value for each voxel. Although participants covertly or overtly pursued visual targets at different speeds (0.5 and 0.8 Hz), we did not distinguish between these conditions in this analysis. We used a threshold of p < 0.05 (FWE corrected) based on a previous study with hierarchical Bayesian estimation^20^. We used the yielded statistical parametric map as prior information about the cortical current variance for hierarchical Bayesian estimation.

Since one participant wasn’t able to concentrate during the experiment due to breathing difficulty caused by the bite bar, he was removed from the data analysis.

### MEG data analysis

Slow DC drift components of MEG data were removed using reference sensors. For each participant, each trial was extracted from − 0.4 s before to 4.0 s after the onset of target motion. We rejected trials in which MEG signals from all channels exceeded 95% of the recordable range. The remaining trials of the seven participants were 97.61%. These remaining data were used for analysis. We removed one participant who gave up the final session (control task) and moved his eyes for most trials during the covert task.

### Head and source models

We constructed a polygon cortical surface model for all participants using FreeSurfer software (version 5.0.0; http://surfer.nmr.mgh.harvard.edu/)^35^ with a T1 structural image for each. The number of cortical surface dipoles of the participants was 20,004. Cortical current sources were located on vertex points of the cortical surface model, and current sources were oriented perpendicular to the cortical surface. A positive current was defined as one directed toward the inside of the cortex^36^. The main noise sources of eye movements and heartbeats were assumed to be the center of each eyeball and the heart’s arterial side. The positions of each eyeball were obtained from the T1 structural image by visual inspection. The precise location from the origin at the brain’s center is unknown because it depends on the neck angle. Therefore, approximate positional values ((*x*,*y*,*z*) = (1.5, 0.0, 35.0) [cm]) were set as the main noise source of the heart’s location. Each extra-brain source was modeled using the three-dimensional resultant dipole current in the x–y–z direction. Nine dipoles (three extra-brain sources × three directions) were located there using the procedure of Morishige et al.^20^.

### Cortical and extra-brain source current estimation

We calculated cortical and extra-brain source currents using an extra-dipole method^20^ based on a hierarchical Bayesian method^37^ and simultaneously estimated cortical and extra-brain source currents by placing the dipoles on both the cortical and extra-brain sources. This method can estimate cortical currents from the MEG data contaminated by extra-brain sources. Time series of estimated currents were smoothed using a nine-period simple moving average, resampled to 200 Hz, and used for the following data analysis.

### Sparse linear regression

A sparse linear regression (SLiR) method was used to predict time series of target positions and velocities from estimated cortical currents^21,34,38^. We only introduced sparsity to spatial input dimensions in order to narrow down cortical current dipoles. We did not introduce any sparsity to temporal input dimensions for pooling. We assumed automatic relevance determination (ARD) prior to the spatial dimension^39^ and used the following regression expressions:

$${x}^{\text{pos}}\left(t\right)={\sum }_{i=1}^{N}{\sum }_{j=0}^{10}{w}_{ij}^{\text{pos}}{J}_{i}\left(\left(t-1\right)-6\times j\right)+{w}_{0}^{\text{pos}}$$

and

$${x}^{\text{vel}}\left(t\right)={\sum }_{i=1}^{N}{\sum }_{j=0}^{10}{w}_{ij}^{\text{vel}}{J}_{i}\left(\left(t-1\right)-6\times j\right)+{w}_{0}^{\text{vel}},$$

where ${x}^{\text{pos}}$ and ${x}^{\text{vel}}$ are target positions and velocities, respectively. *N* is the number of cortical current sources used for the prediction. ${w}_{ij}^{\text{pos}}$ and ${w}_{ij}{\text{vel}}$ indicate weight values for the *i*-th cortical current with time delay *j* * 6. ${w}_{0}^{\text{pos}}$ and ${w}_{0}^{\text{vel}}$ indicate residual errors. We predicted the position and velocity at time *t* from the estimated cortical currents during the time window between − 305 ((*t* − 1 − 6) × 10) and − 5 ms (*t* − 1). Eleven points were selected at time-regular intervals from the time window, and cortical currents at the points were used for predictions (SLiR parameters: Tau = 6 [ms], Dtau = 10 [dimension], and Tpred = 5 [ms]). We used cortical dipole currents on the fMRI activation areas for predicting the time series of visual target positions and velocities. In this Bayesian estimation method, we assumed a spatial smoothness constraint on the cortical current distribution at 20,004 dipole locations. To introduce spatial smoothness, we employed a smoothing matrix $\boldsymbol{W}$ (20,004 cortical dipoles × 5004 reduced dipoles) and an auxiliary variable $\boldsymbol{Z}$ (5004 reduced dipoles × 4400 sampling indices) and

1 $$\boldsymbol{J}=\boldsymbol{W}\boldsymbol{Z},$$

where $\boldsymbol{J}$ is the cortical current amplitudes. Equation (1) can be replaced by

2 $$\boldsymbol{B}=\widehat{\boldsymbol{G}}\boldsymbol{Z},$$

where $\widehat{\boldsymbol{G}}=\boldsymbol{G}\boldsymbol{W}$ is a smoothed lead field matrix. Therefore, the inverse problem becomes the problem of estimating $\boldsymbol{Z}$ with smoothed lead field matrix $\widehat{\boldsymbol{G}}$. After estimating $\boldsymbol{Z}$, cortical current $\boldsymbol{J}$ is obtained from Eq. (1). Spatial smoothing with a Gaussian weighting function does not taint the information of the $\boldsymbol{Z}$ currents^40^. For improving the computational efficiency, we used the $\boldsymbol{Z}$ currents as cortical currents when predicting the time series of the target positions and velocities.

## Supplementary Information

Supplementary Information.

## Data Availability

Datasets generated and analyzed during this study are available from the corresponding author upon request.
