# Supplementary material for: Common cortical areas have different neural mechanisms for covert and overt visual pursuits
Source: Sci Rep. 2021 Jul 6;11:13933. doi: 10.1038/s41598-021-93259-9 (PMC8260617; doi:10.1038/s41598-021-93259-9)
Supplement: Supplementary file 1 — Supplementary Information. [file 41598_2021_93259_MOESM1_ESM.pdf]

Supplementary information

Common cortical areas have different neural mechanisms for covert and overt visual pursuits

Ken-ichi Morishige[1,2,*], Nobuo Hiroe[2], Masa-aki Sato[2], and Mitsuo Kawato[1,3]

[1] *Department of Intelligent Robotics, Toyama Prefectural University, 5180 Kurokawa, Imizu-shi, Toyama 939-0398, Japan*

[2] *Neural Information Analysis Laboratories, Advanced Telecommunications Research Institute International, 2-2-2 Hikaridai, Keihanna Science City, Kyoto 619-0288, Japan*

[3] *Brain Information Communication Research Laboratory Group, Advanced Telecommunications Research Institute International, 2-2-2 Hikaridai, Keihanna Science City, Kyoto 619-0288, Japan*

***Correspondence to:**

Ken-ichi Morishige

Department of Intelligent Robotics, Toyama Prefectural University

5180 Kurokawa Imizu, Toyama 939-0398, Japan

E-mail: kmorishi@pu-toyama.ac.jp

1 Cross-validation for model parameter tuning and model evaluation

We implemented a 10-fold cross-validation with nesting for tuning the sparseness parameter and validating our model (Supplementary Fig. S1). Data were split into ten groups that contained an equal number of trials. Each group was used as a test dataset, and the rest were used as a training dataset. Test datasets were split into ten sub-groups, each of which was used as a test sub-dataset once, while the rest were used as training sub-datasets. By systematically varying the threshold for sparseness ($a_{\min}$= 10^{-7}, $10^{-6.5}$, 10^{-6}, $10^{-5.5}$, 10^{-5}, $10^{-4.5}$, 10^{-4}, $10^{-3.5}$, 10^{-3}), we predicted time series of the velocities from the first sub-dataset using SLiR. After that, we calculated the determination coefficients between presented and predicted time series of velocities using the following equation:

$$R^2 = 1 - \frac{\sum_{t=1}^{T}\left(x^{\mathrm{act}}(t) - x^{\mathrm{rec}}(t)\right)^2}{\sum_{t=1}^{T}\left(x^{\mathrm{act}}(t) - \overline{x^{\mathrm{act}}}\right)^2},$$

where x^{act} and x^{rec} were the presented and predicted values, respectively. $\overline{x^{\mathrm{act}}}$ and $\overline{x^{\mathrm{rec}}}$ were the trial mean values of the presented and predicted velocities, respectively.

We considered the $a_{\min}$ with the largest determination coefficient among them as the sub-optimal threshold of $a_{\min}$ for the sub-dataset. We also conducted the same calculation for the other nine sub-datasets and obtained 900 sub-optimal thresholds of $a_{\min}$ (9 candidates of $a_{\min}$×10 sub-datasets×10 internal-loops). In order to determine the optimal threshold $\mathrm{a}_{\min}$ of sparseness for SLiR, we counted frequencies of sub-optimal $\mathrm{a}_{\min}$ selected from the nine candidates and plotted each selected number on frequency histograms (Supplementary Fig. S2). The candidate $\mathrm{a}_{\min} = 10^{-5}$ was the most selected, regardless of which dataset was used for 10-fold nested cross-validations. In this paper, we considered this value as the optimal $\mathrm{a}_{\min}$ and used it to predict time series of target positions and velocities from the first dataset as a test dataset.

We conducted the same calculation for the remaining nine datasets and predicted

the time series of target positions and velocities from each remaining dataset. Mean values of the ten selected determination and correlation coefficients were used for evaluating our models for SLiR accuracy. We calculated correlation coefficients using the following equation:

$$r = \frac{\sum_{t=1}^{T}\left(x^{\text{act}}(t) - \overline{x^{\text{act}}}\right)\left(x^{\text{rec}}(t) - \overline{x^{\text{rec}}}\right)}{\sqrt{\sum_{t=1}^{T}\left(x^{\text{act}}(t) - \overline{x^{\text{act}}}\right)^2}\sqrt{\sum_{t=1}^{T}\left(x^{\text{rec}}(t) - \overline{x^{\text{rec}}}\right)^2}}.$$

When investigating the generalization of different tasks between the training and test data, 10-fold cross-validation was not used. Instead, all trials of the task were used as training or test data. These calculations of the nested cross-validation for each task and participant required a large amount of time ([1 task, 20 days] × [5 types of tasks] × [6 participants] = 600 days using the parallel computing of 17 CPU servers; CPU: Xeon E3-1270 3.6 GHz, Mem: 16 GB). To reduce the computational time, we calculated an optimal sparseness threshold a_{min} from one typical participant's data during the overt pursuit 0.5 Hz task and used this optimal value for the data analysis of other tasks and participants.

Orienting of attention and maintenance of attention differ in their relationship to the oculomotor system. Belopolsky and Theeuwes investigated the relationship between attention and saccades using a paradigm that allowed simultaneous measurement of attentional allocation and saccade preparation. They proposed a theory that attention is oriented through activation of an oculomotor program, but after attention is shifted, the oculomotor program can be suppressed if the probability of its execution is low [1,2].

Our study mainly focuses on the maintenance of attention. In order to discuss the time period after covertly catching up with the moving target, we excluded the time series of data before 600 ms when calculating the correlation and determination coefficients,

because mean saccadic reaction time is about 560 – 575 ms, which is below 600 ms with oculomotor priming[1]. Additionally, we also excluded the effect of visually evoked responses that were observed in the early visual cortex around 100 ms after onset when the target started to move.

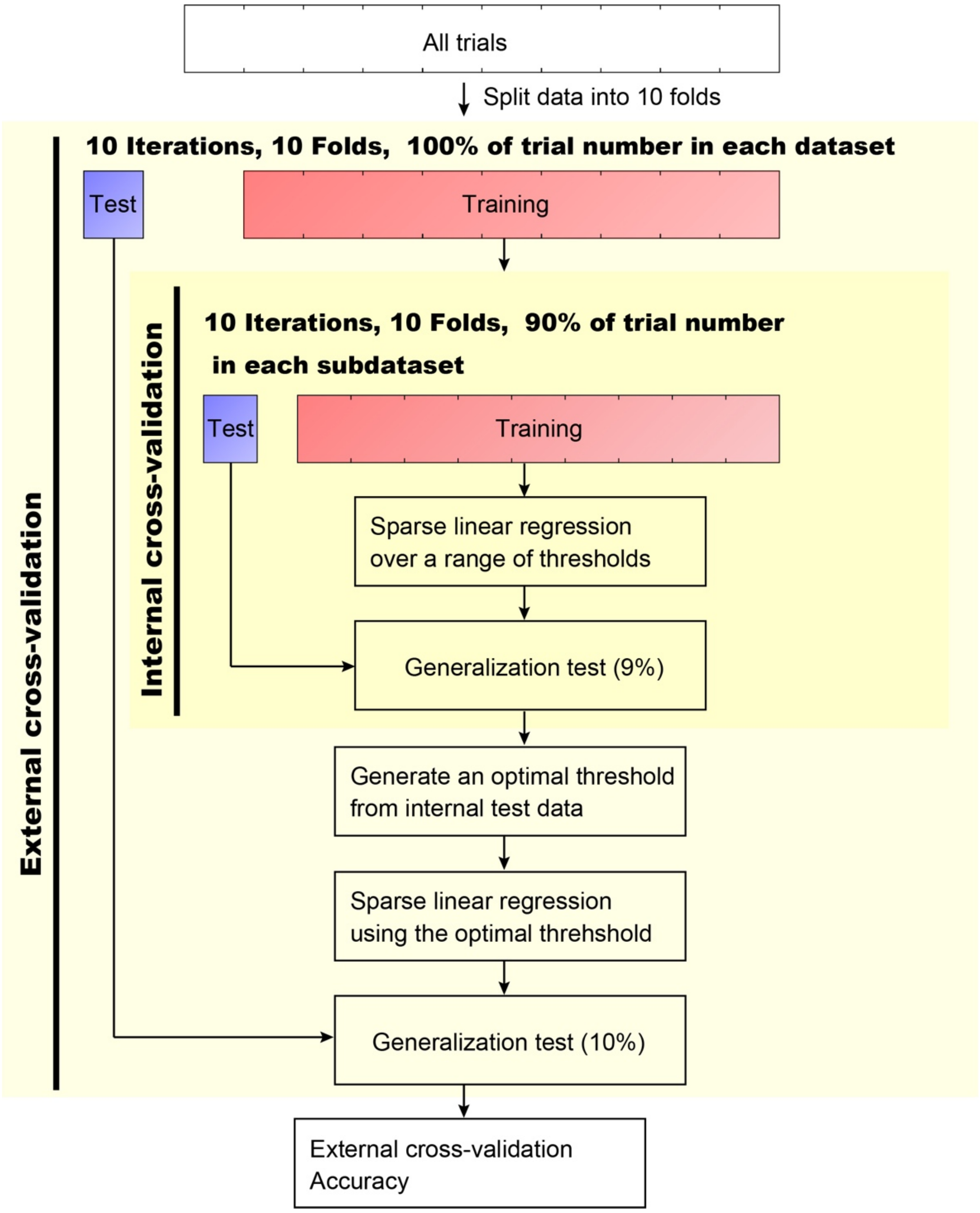

Figure S1. Diagram of analysis protocol.

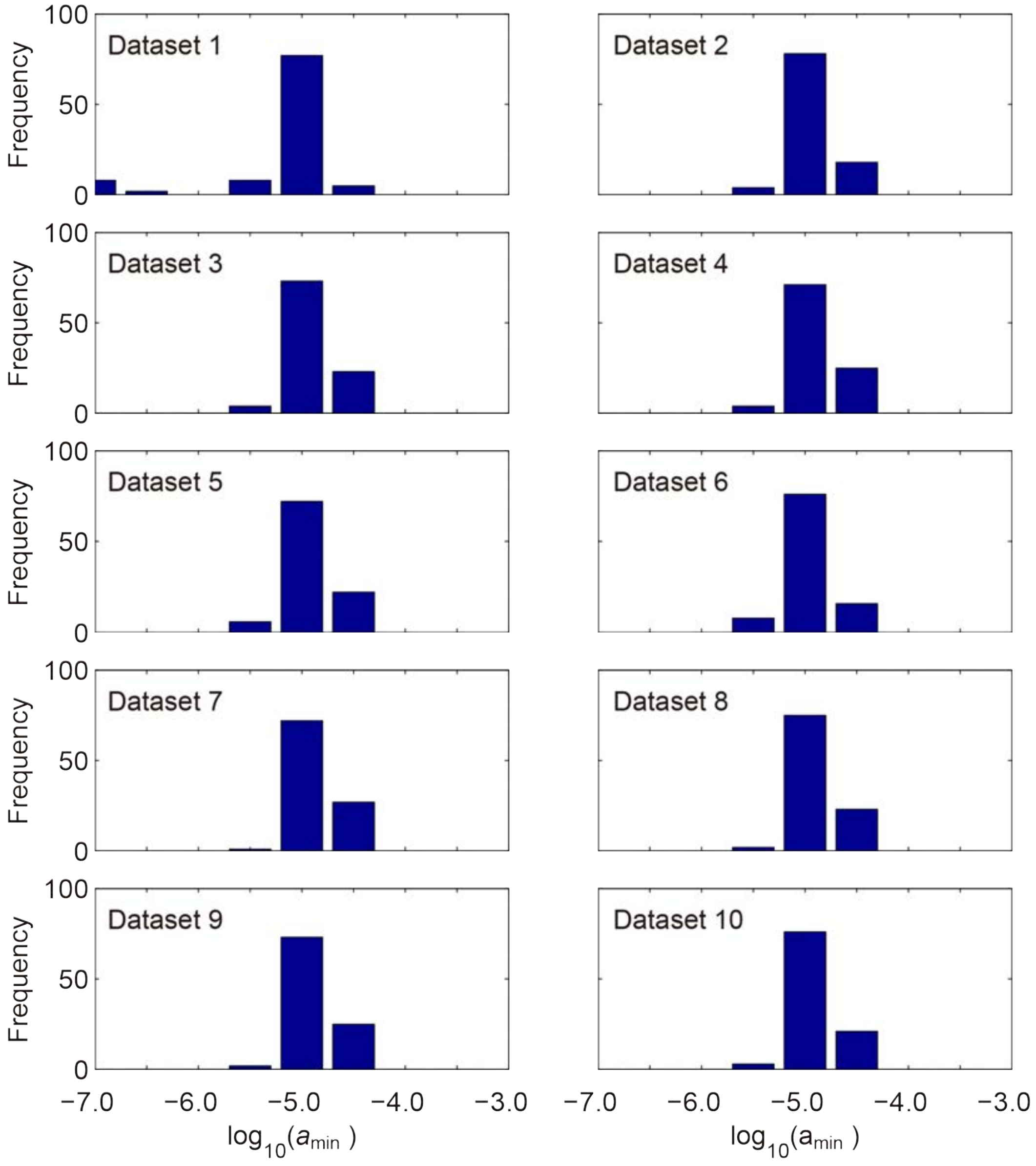

Figure S2. Frequency histograms of sub-optimal sparseness parameters selected using 10-fold nested cross validation.

2 Correlation and determination coefficients calculated from presented and reconstructed time series of target kinetics

We show mean values of correlation and determination coefficients of entire subjects (Tables S1–S4). These indices were calculated from presented and reconstructed time series of target positions and velocities.

Table S1. Mean values of correlation coefficients (position)

			Test				
			Control	Covert		Overt	
			0.5 Hz	0.5 Hz	0.8 Hz	0.5 Hz	0.8 Hz
Training	Control	0.5 Hz	0.84±0.05	0.78±0.07	0.43±0.14	0.58±0.51	0.38±0.51
	Covert	0.5 Hz	0.80±0.09	0.82±0.06	0.44±0.15	0.58±0.44	0.50±0.37
		0.8 Hz	0.42±0.09	0.44±0.14	0.89±0.03	0.54±0.31	0.72±0.14
	Overt	0.5 Hz	0.38±0.22	0.42±0.16	0.38±0.29	0.98±0.02	0.90±0.03
		0.8 Hz	0.37±0.31	0.29±0.30	0.49±0.14	0.93±0.04	0.97±0.01

Table S2. Mean values of R-square (position)

			Test				
			Control	Covert		Overt	
			0.5 Hz	0.5 Hz	0.8 Hz	0.5 Hz	0.8 Hz
Training	Control	0.5 Hz	0.22+0.04	0.19±0.07	0.13±0.06	0.15±0.17	0.17±0.27
	Covert	0.5 Hz	0.26±0.06	0.24±0.05	0.16±0.09	0.14±0.19	0.12±0.14
		0.8 Hz	0.07±0.01	0.07±0.03	0.29±0.09	0.11±0.12	0.24±0.12
	Overt	0.5 Hz	0.06±0.04	0.07±0.06	0.05±0.10	0.92±0.04	0.36±0.98
		0.8 Hz	0.04±0.04	0.03±0.04	0.08±0.03	0.54±0.11	0.85±0.04

Table S3. Mena values of correlation coefficients (velocity)

			Test				
			Control	Covert		Overt	
			0.5 Hz	0.5 Hz	0.8 Hz	0.5 Hz	0.8 Hz
Training	Control	0.5 Hz	0.87±0.04	0.84±0.09	0.62±0.14	0.44±0.27	0.32±0.46
	Covert	0.5 Hz	0.83±0.07	0.87±0.05	0.61±0.15	0.38±0.23	0.16±0.35
		0.8 Hz	0.52±0.13	0.50±0.17	0.86±0.06	0.43±0.20	0.56±0.16
	Overt	0.5 Hz	0.34±0.16	0.40±0.16	0.33±0.10	0.95±0.01	0.84±0.06
		0.8 Hz	0.29±0.22	0.29±0.22	0.49±0.05	0.77±0.08	0.95±0.00

Table S4. Mean values of R-square (velocity)

			Test				
			Control	Covert		Overt	
			0.5 Hz	0.5 Hz	0.8 Hz	0.5 Hz	0.8 Hz
Training	Control	0.5 Hz	0.26±0.09	0.24±0.11	0.15±0.07	0.13±0.10	0.07±0.14
	Covert	0.5 Hz	0.31±0.06	0.33±0.09	0.17±0.05	0.04±0.20	-0.09±0.34
		0.8 Hz	0.17±0.10	0.17±0.12	0.32±0.11	0.14±0.10	0.15±0.06
	Overt	0.5 Hz	0.05±0.05	0.07±0.05	0.06±0.02	0.79±0.02	0.63±0.10
		0.8 Hz	0.05±0.05	0.05±0.06	0.08±0.03	0.53±0.10	0.81±0.04

3 Reliability of the statistical index

To examine whether the determination and correlation coefficients were statistically significant, we prepared estimated (original) and temporally-shuffled cortical currents and performed the following permutation tests:

a) If task types were the same between the training and test data, datasets of temporally-shuffled cortical currents were made from the original data by shuffling the time points of each dipole current. The time axis of each dipole was randomly permuted for each trial.
b) We created two groups of data: original and temporally shuffled. We fixed the number of the groups and shuffled all of their data. We prepared 1,000 permuted datasets.
c) We predicted a time series of target positions and velocities using SLiR and calculated determination and correlation coefficients.
d) We investigated significant differences between statistical values (determination and correlation coefficients) calculated from the original and temporally shuffled cortical currents. We used one-tailed tests because we assumed that original values would be higher than temporally shuffled ones.
e) If task types differed between training and test data, we fixed the two datasets and shuffled all of their data. We prepared 1,000 permuted datasets, predicted target positions and velocities, and investigated significant differences using two-tailed tests.

We corrected for multiple testing by using the Benjamini–Hochberg false discovery rate (FDR) correction[3] at $p<0.05$.

4 Reconstruction of time series of target kinetics from estimated cortical currents

We show time series of target positions and velocities reconstructed from estimated cortical currents in Figures S3–S14.

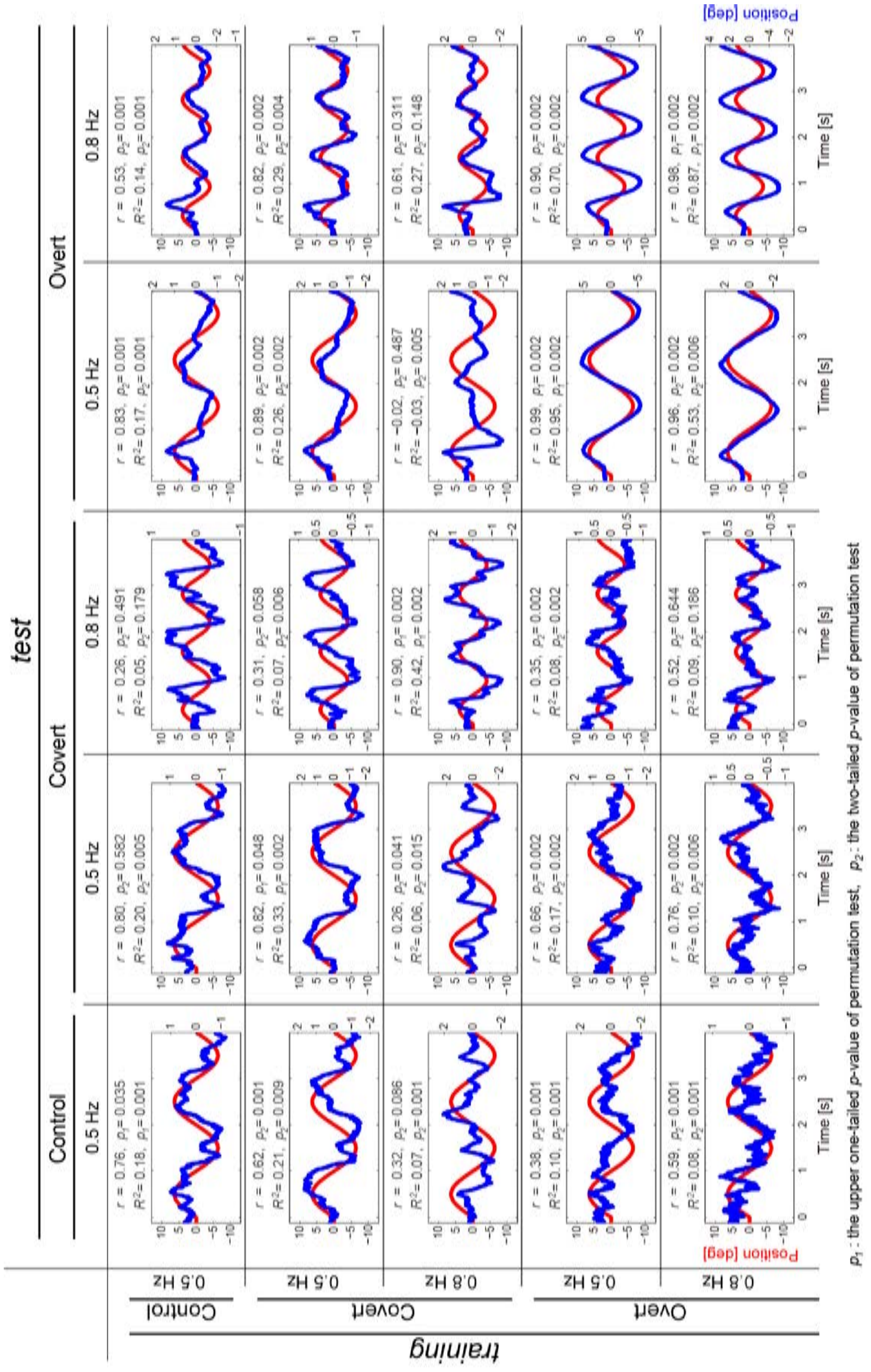

Figure S3. Reconstructed target trajectories (Subj. 1, position)

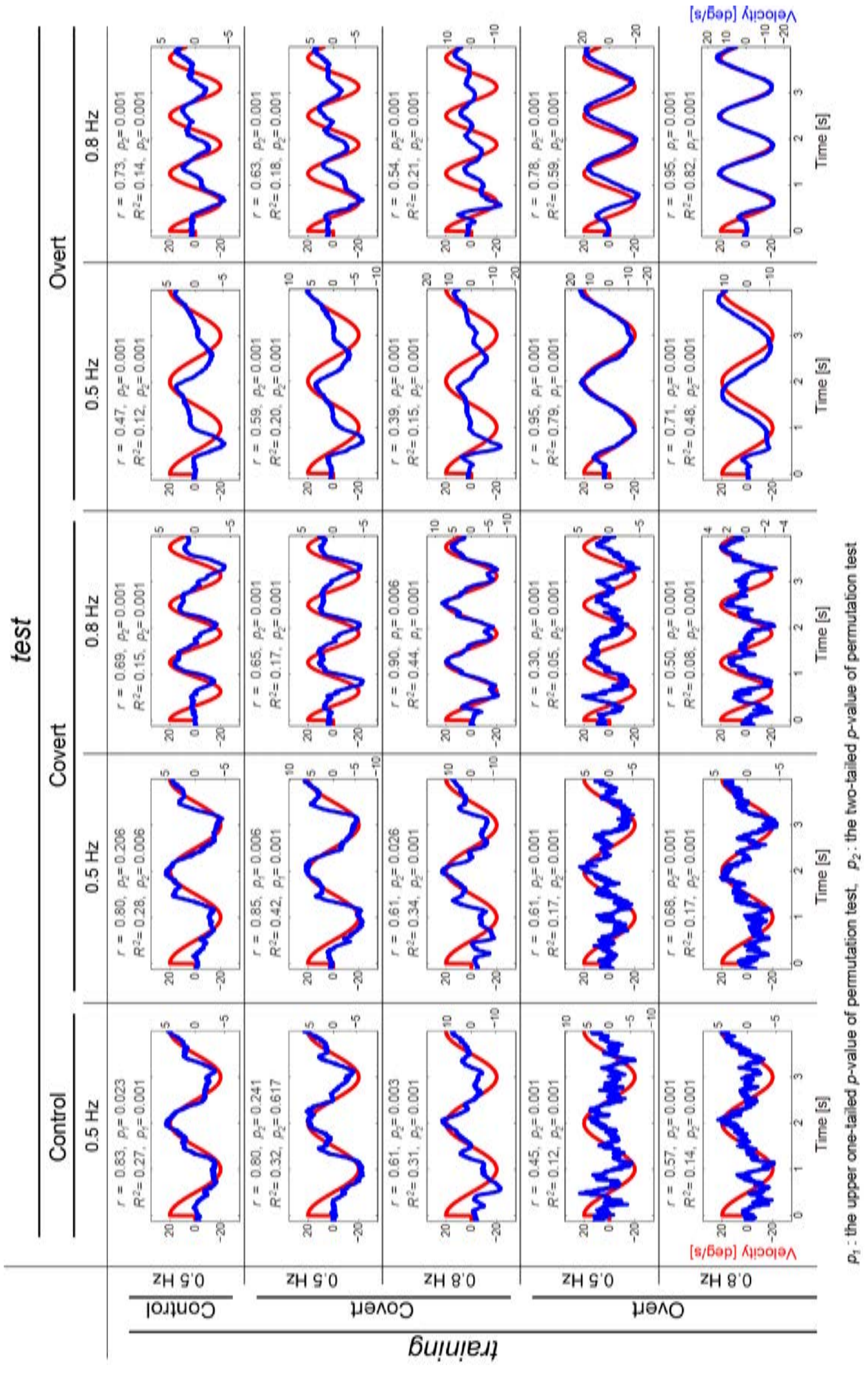

Figure S4. Reconstructed target trajectories (Subj. 1, velocity)

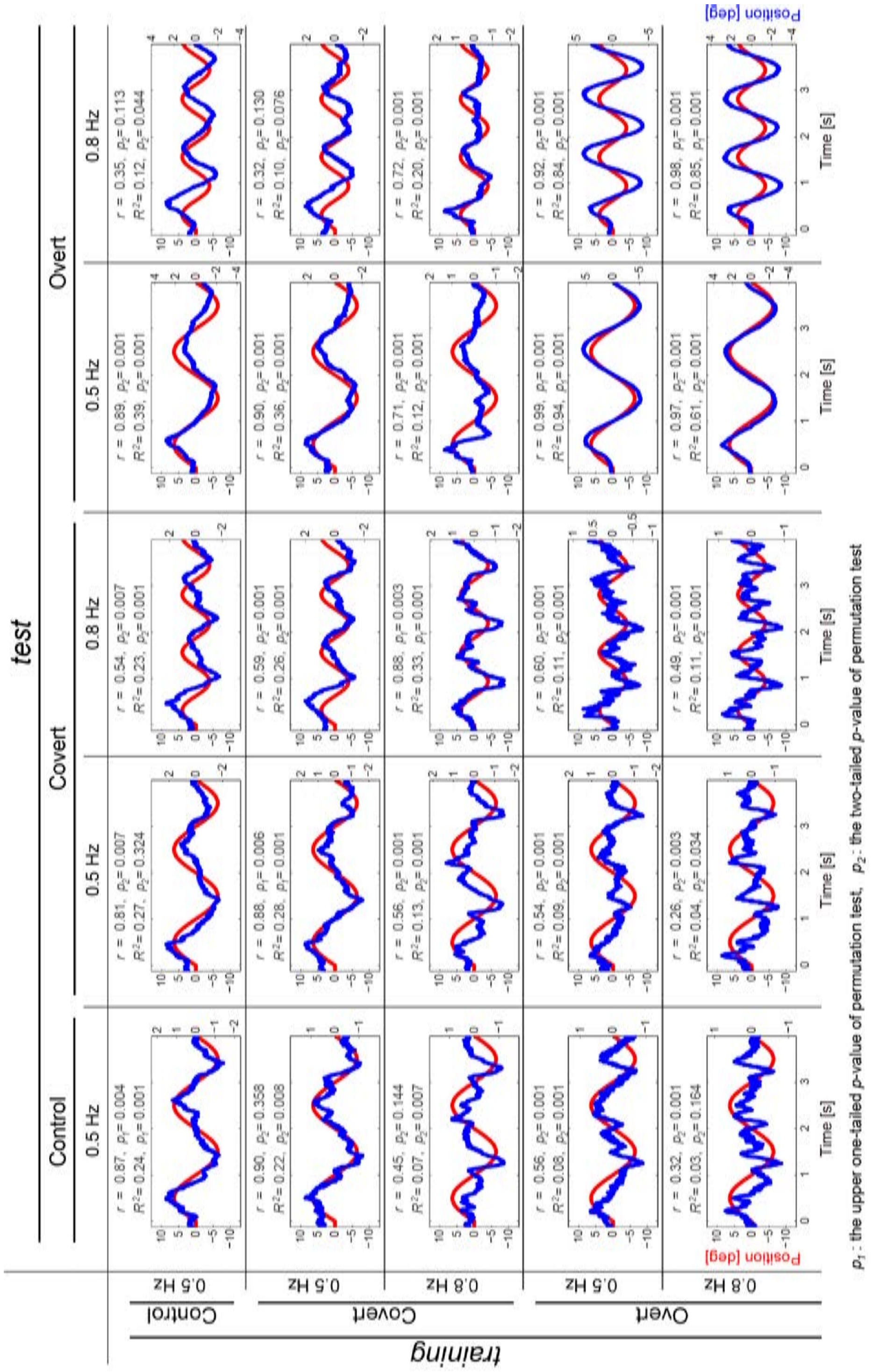

Figure S5. Reconstructed target trajectories (Subj. 2, position)

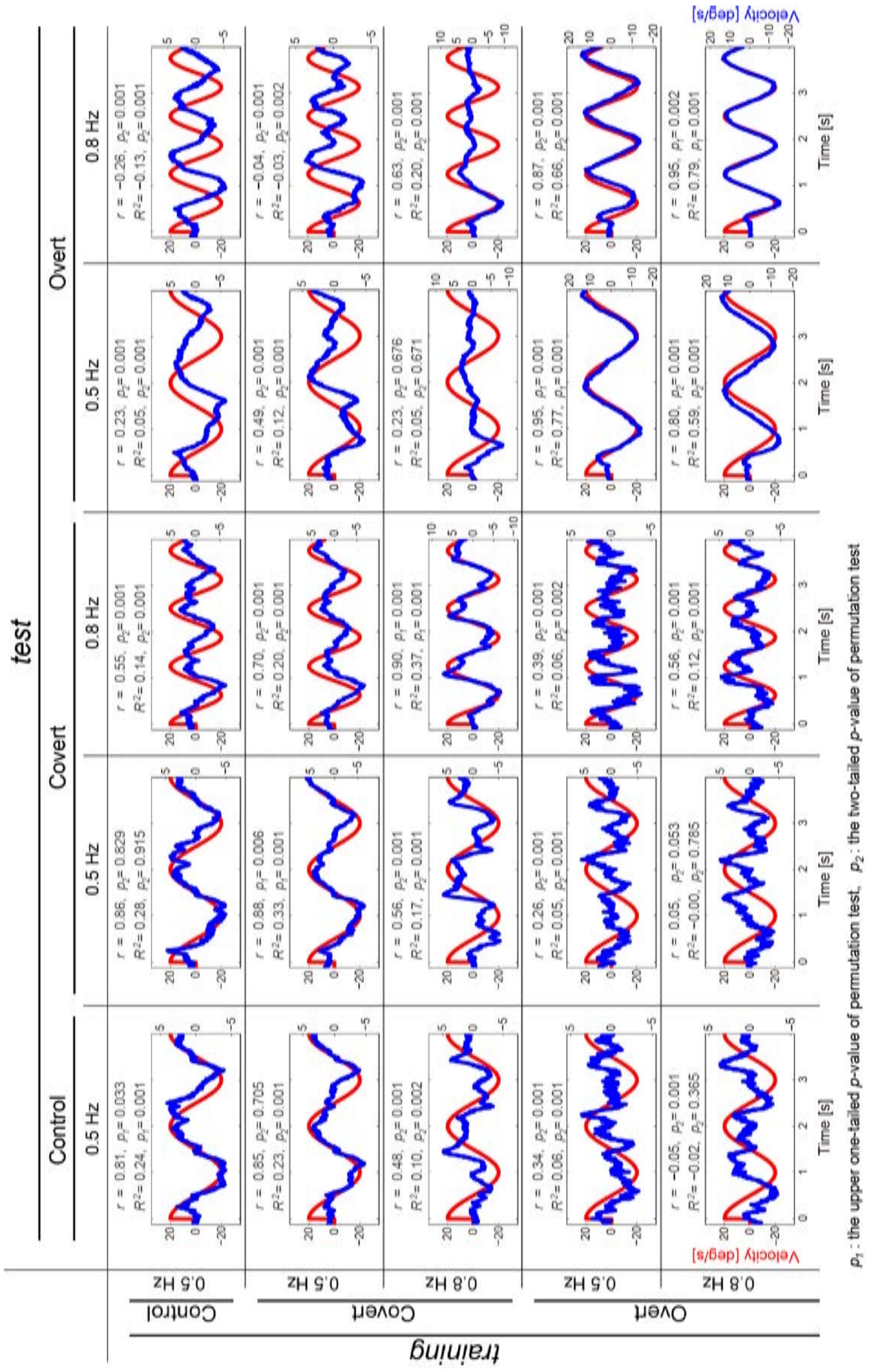

Figure S6. Reconstructed target trajectories (Subj. 2, velocity)

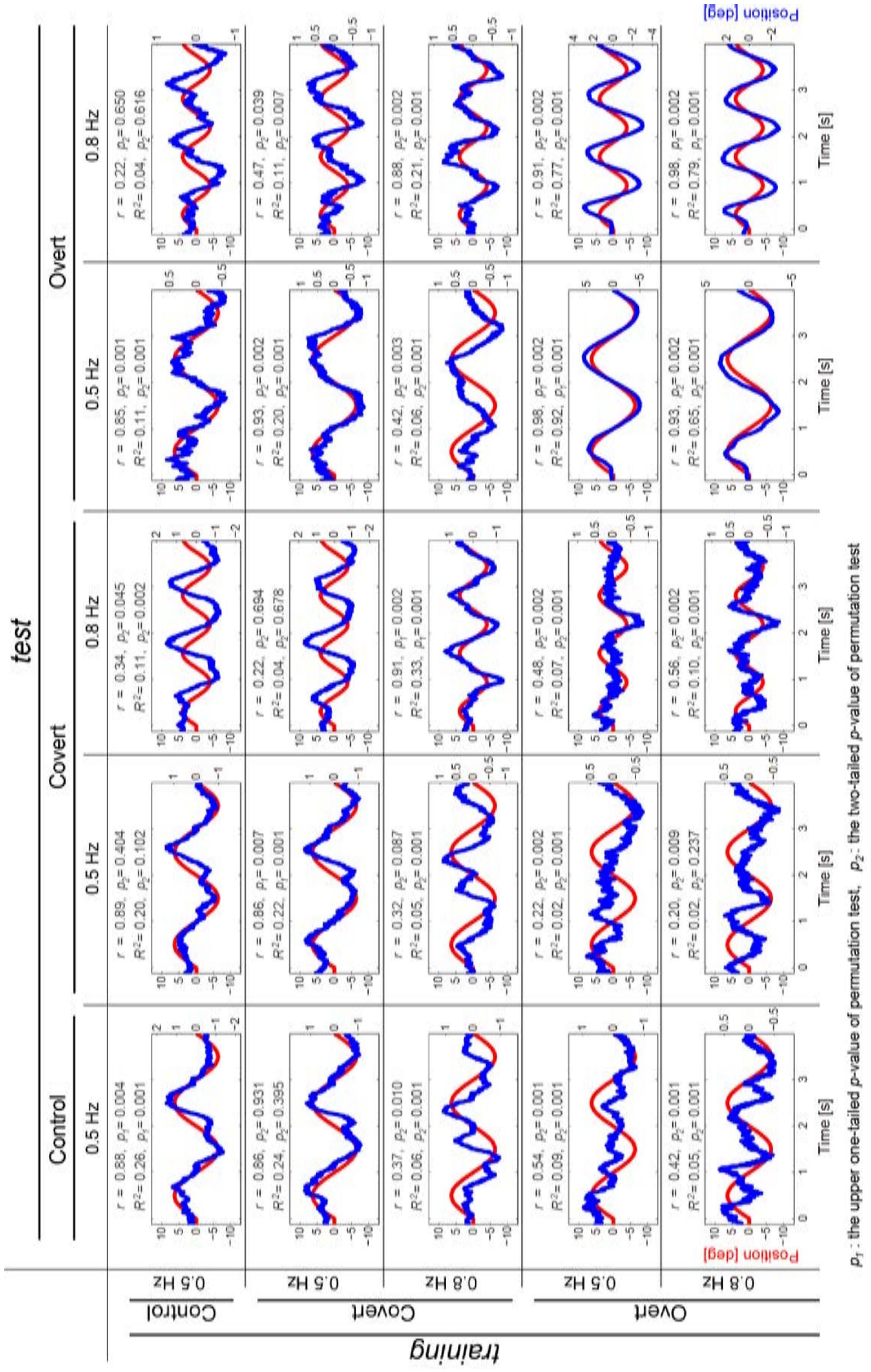

Figure S7. Reconstructed target trajectories (Subj. 3, position)

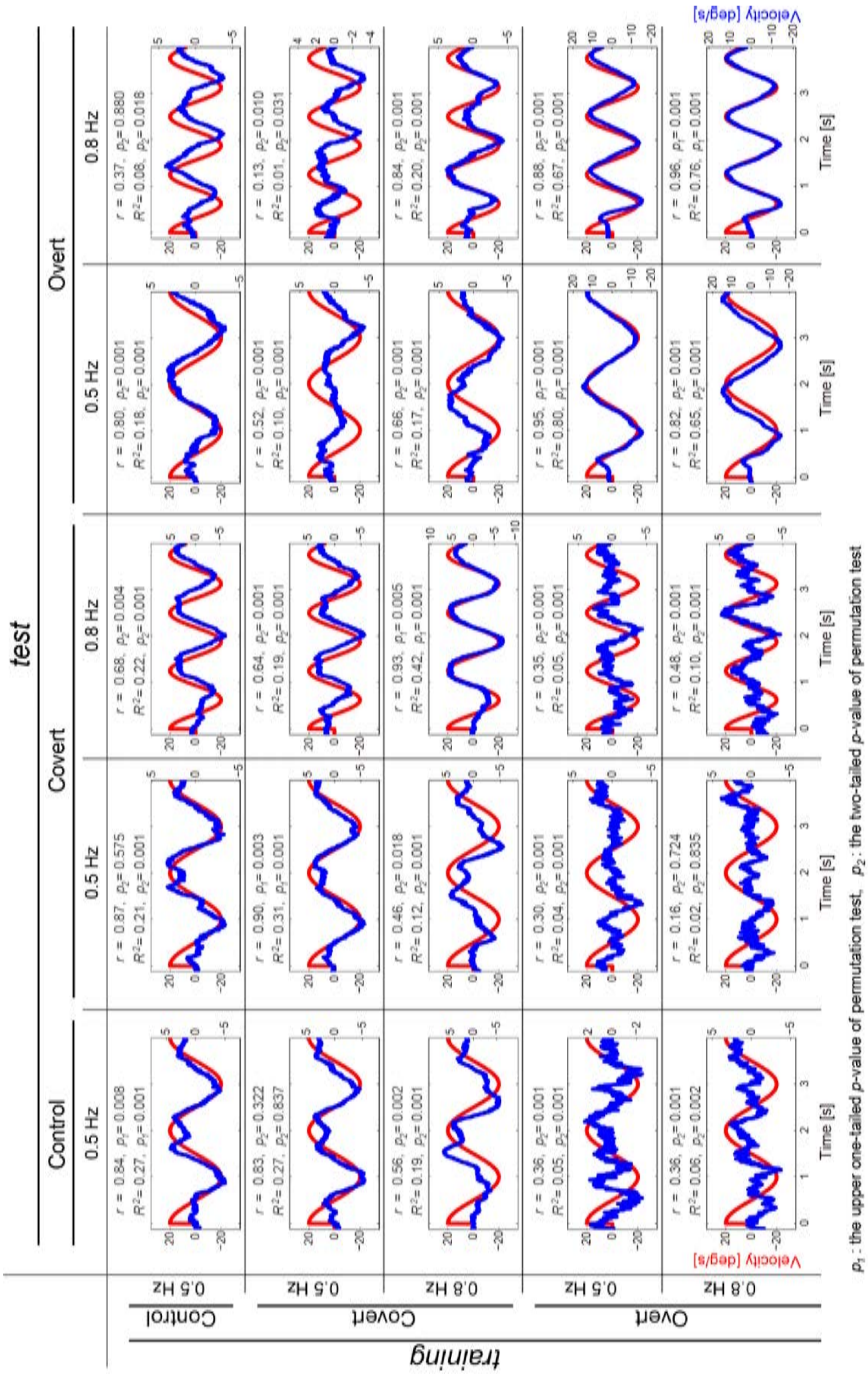

Figure S8. Reconstructed target trajectories (Subj. 3, velocity)

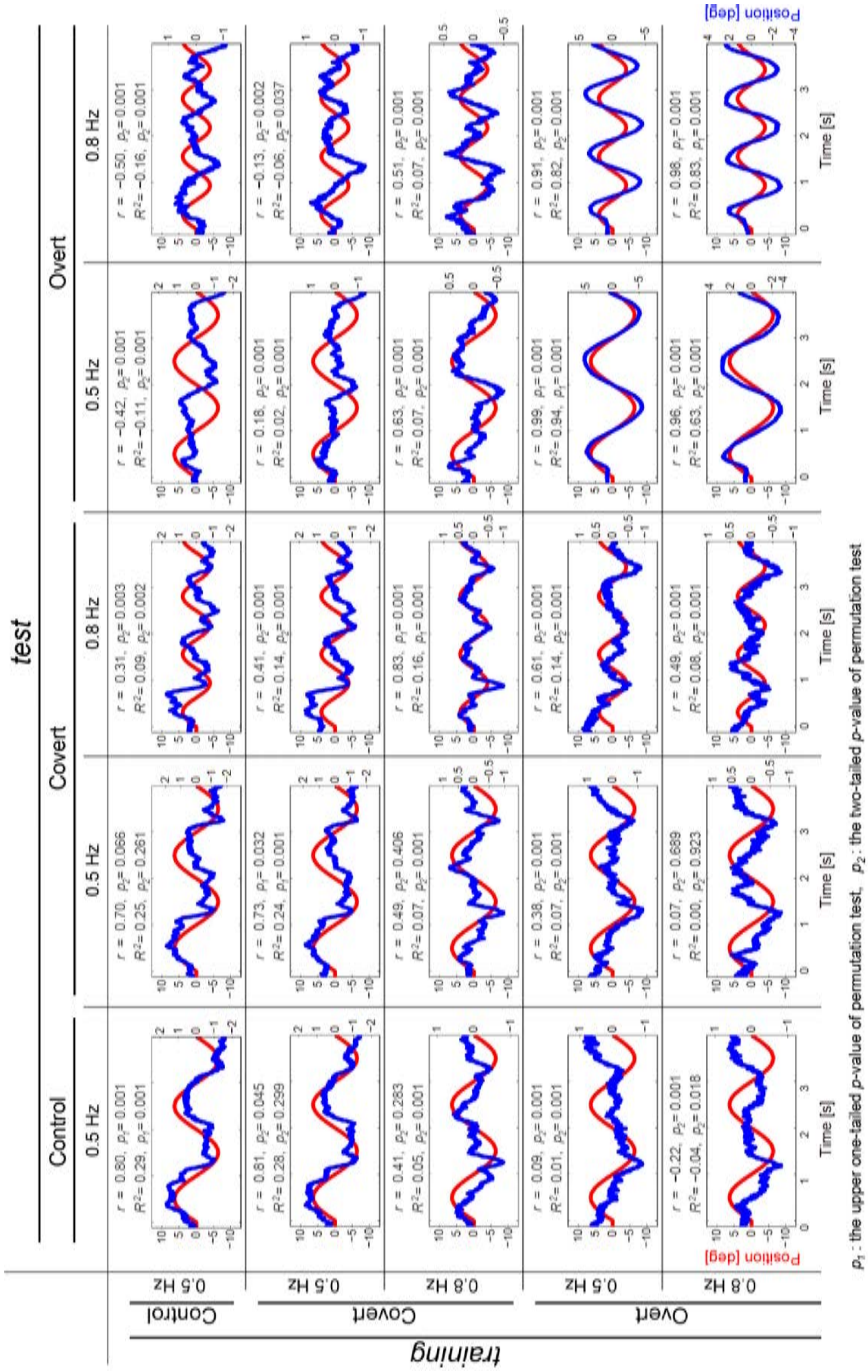

Figure S9. Reconstructed target trajectories (Subj. 4, position)

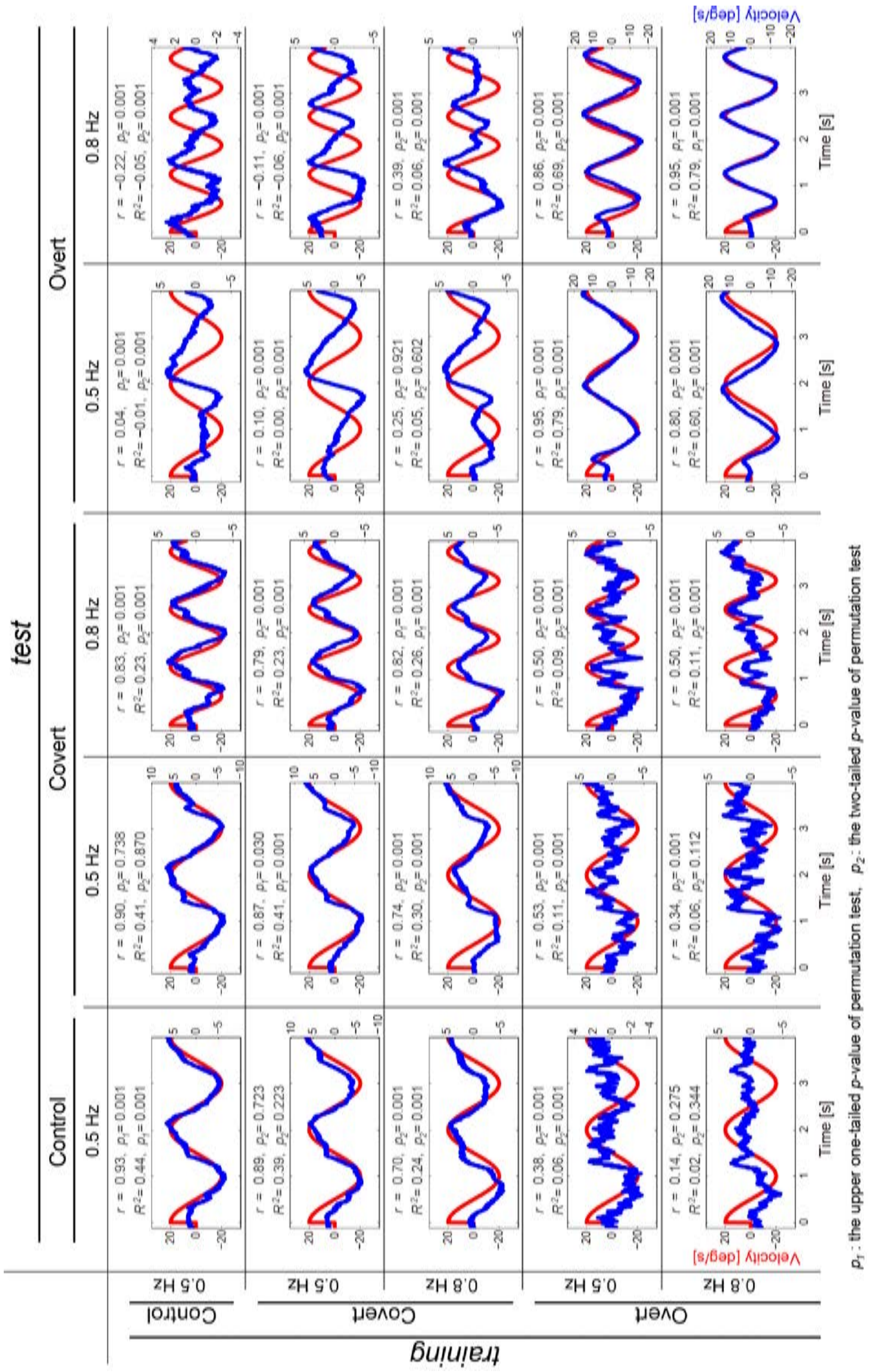

Figure S10. Reconstructed target trajectories (Subj. 4, velocity)

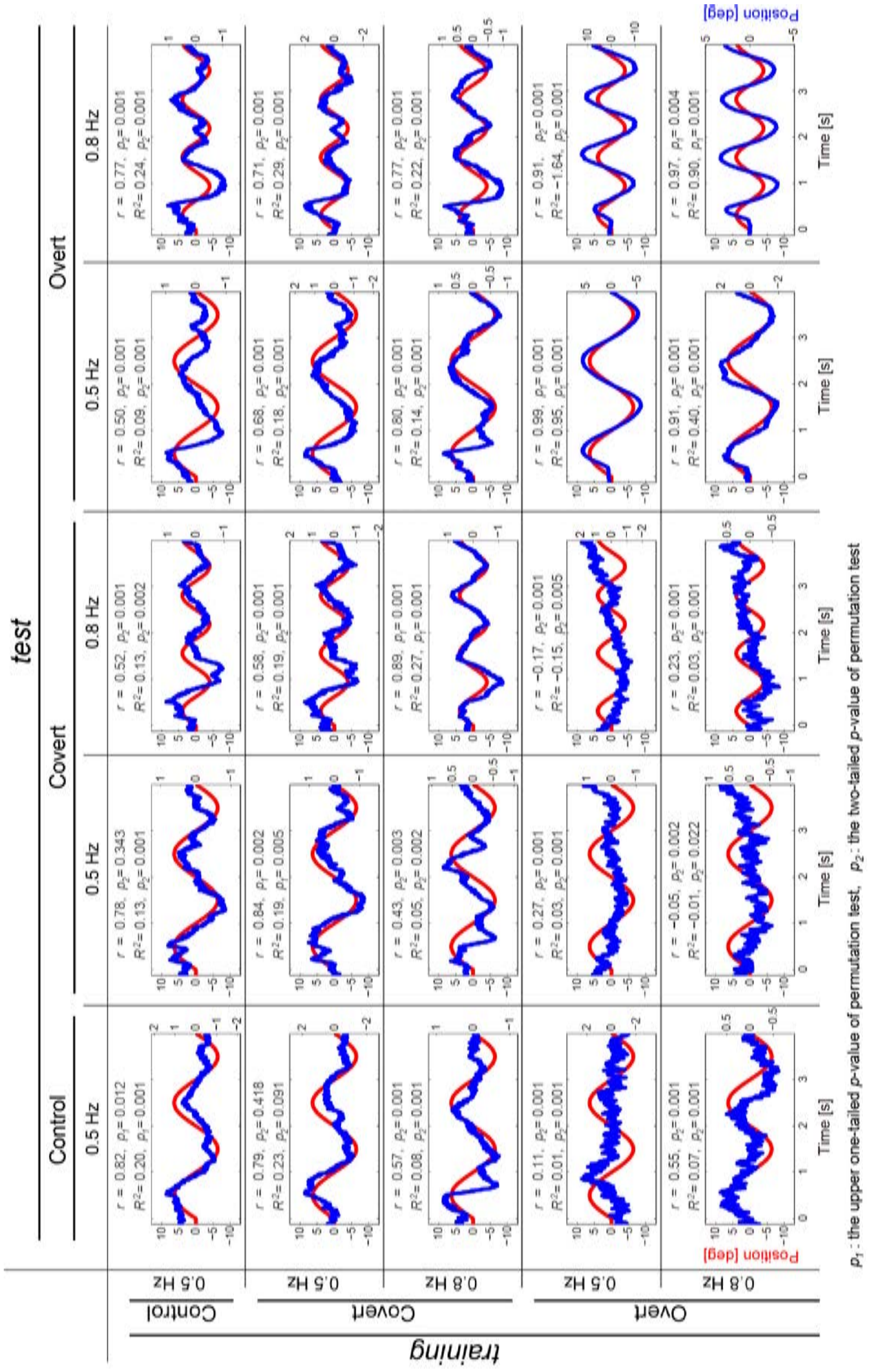

Figure S11. Reconstructed target trajectories (Subj. 5, position)

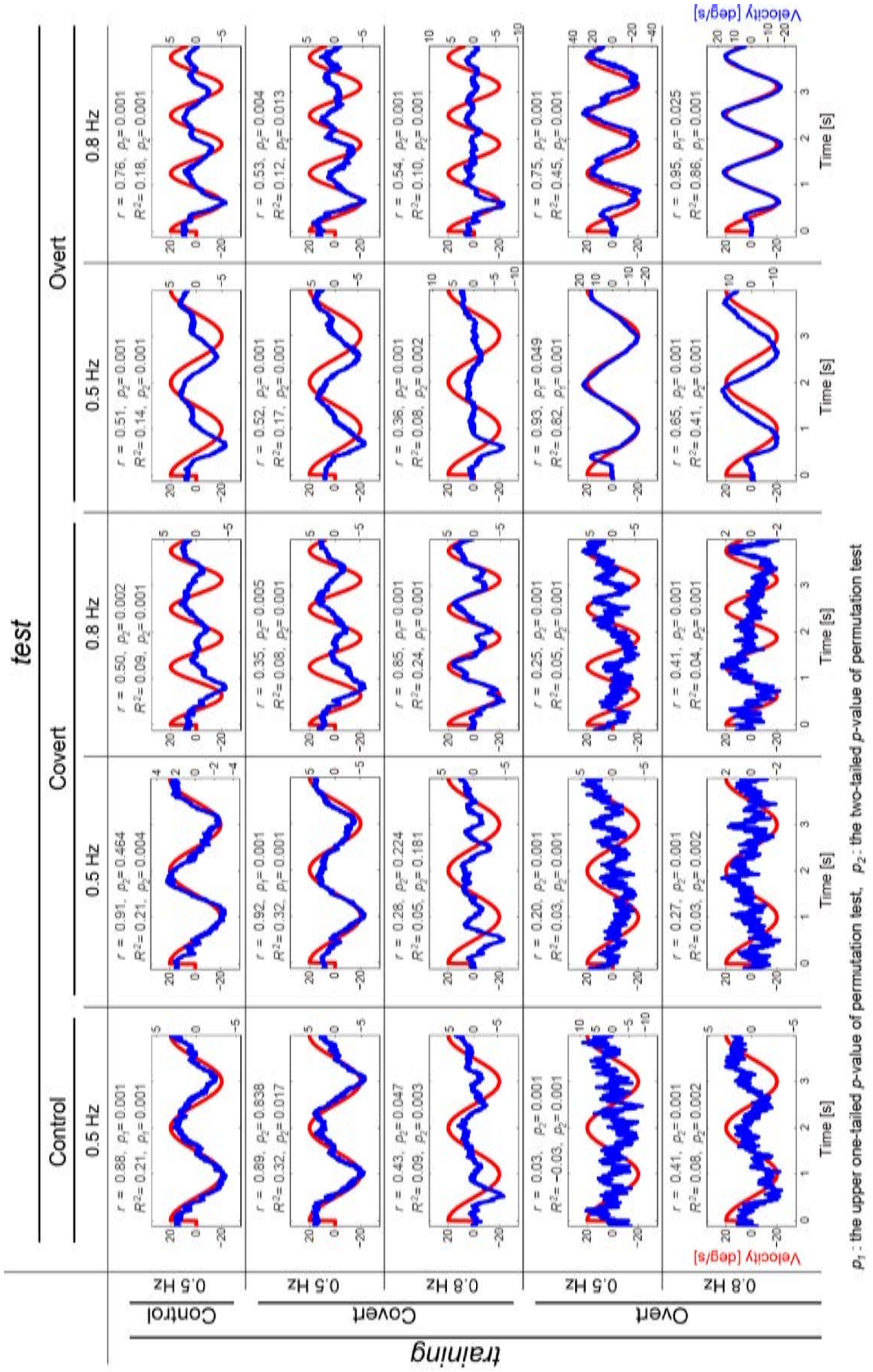

Figure S12. Reconstructed target trajectories (Subj. 5, velocity)

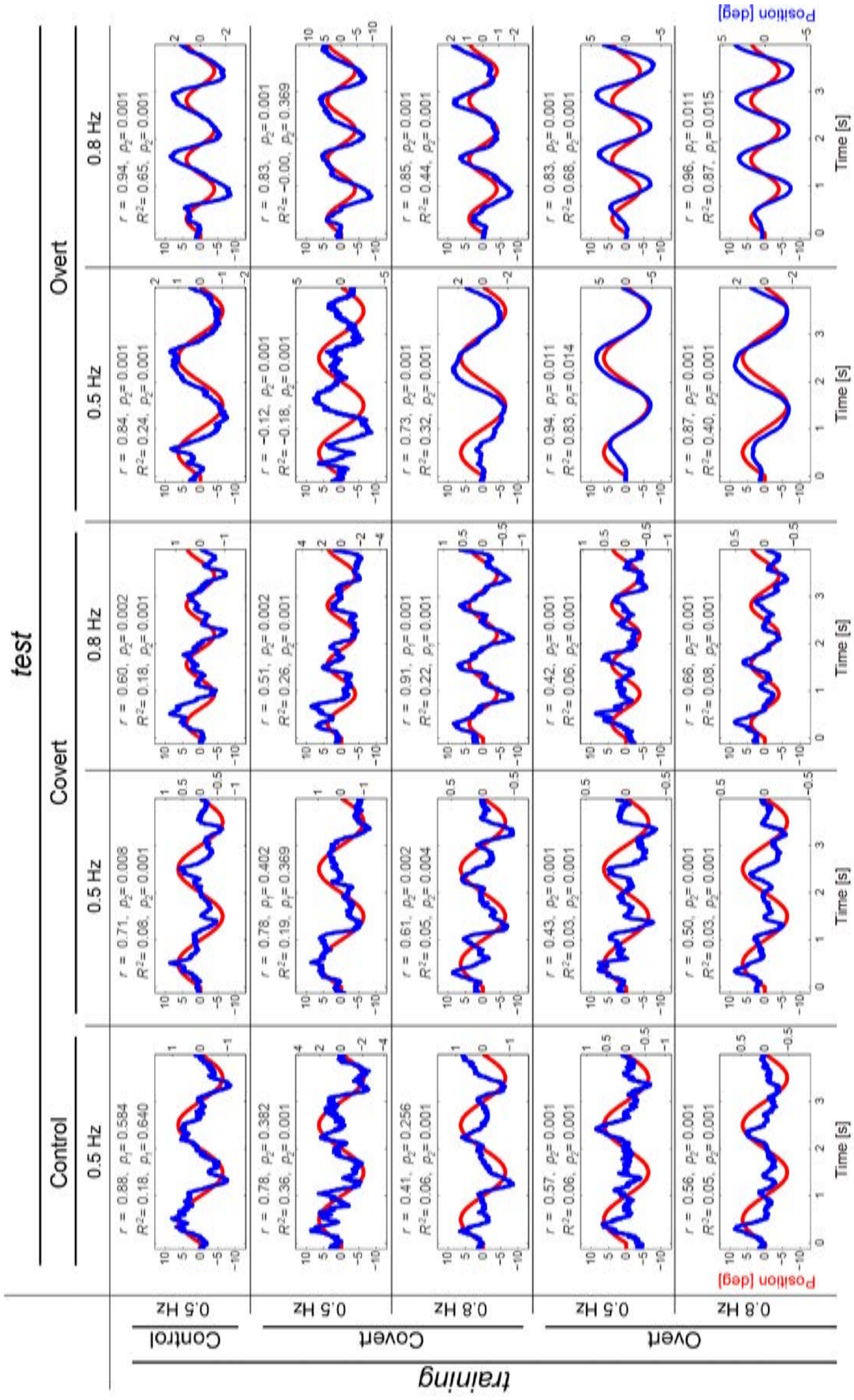

Figure S13. Reconstructed target trajectories (Subj. 6, position)

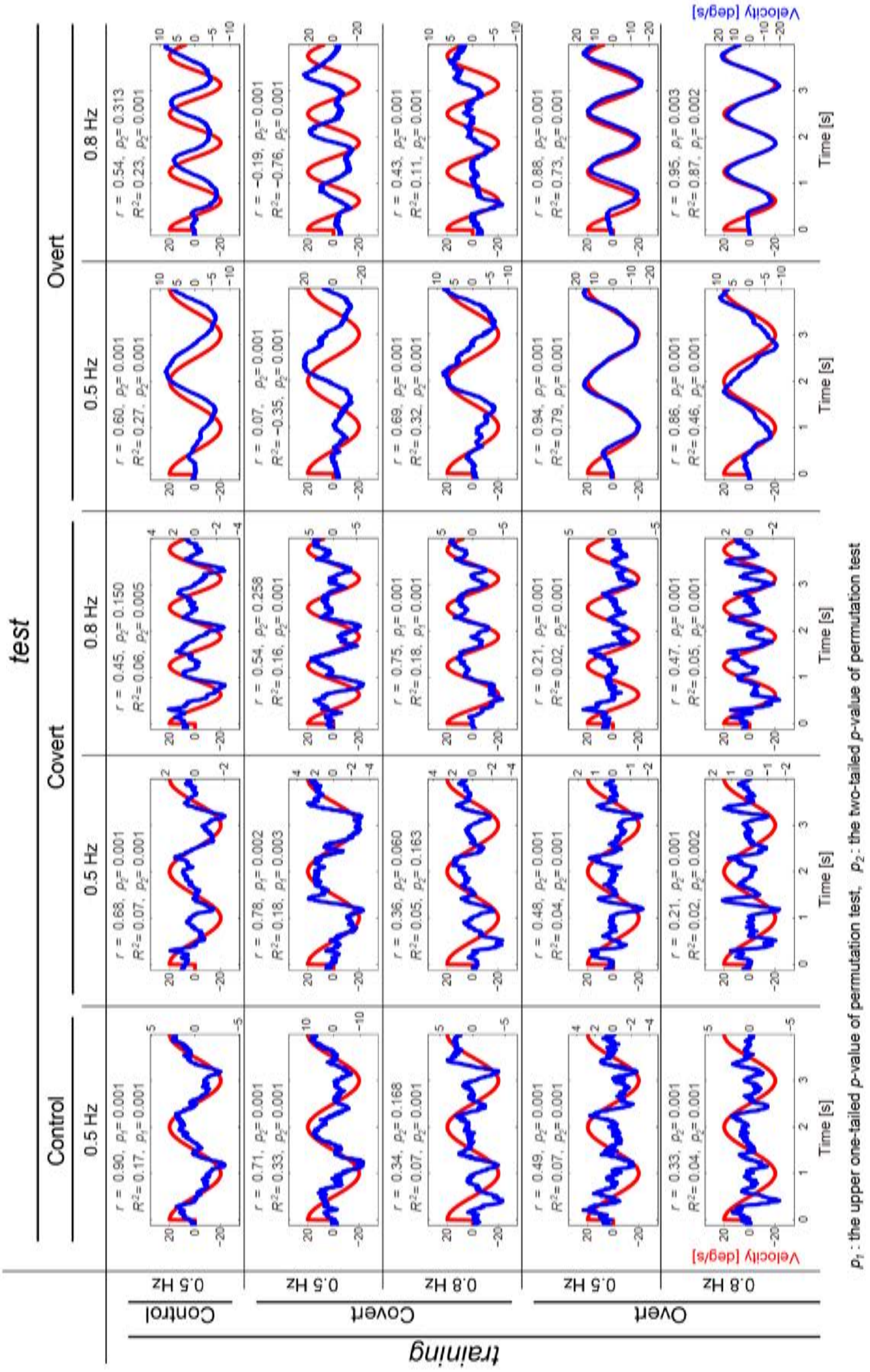

Figure S14. Reconstructed target trajectories (Subj. 6, velocity)

5 Dipole locations selected by SLiR

We show dipole locations selected by SLiR for reconstructing time series of target positions and velocities in Figures S15 and S16.

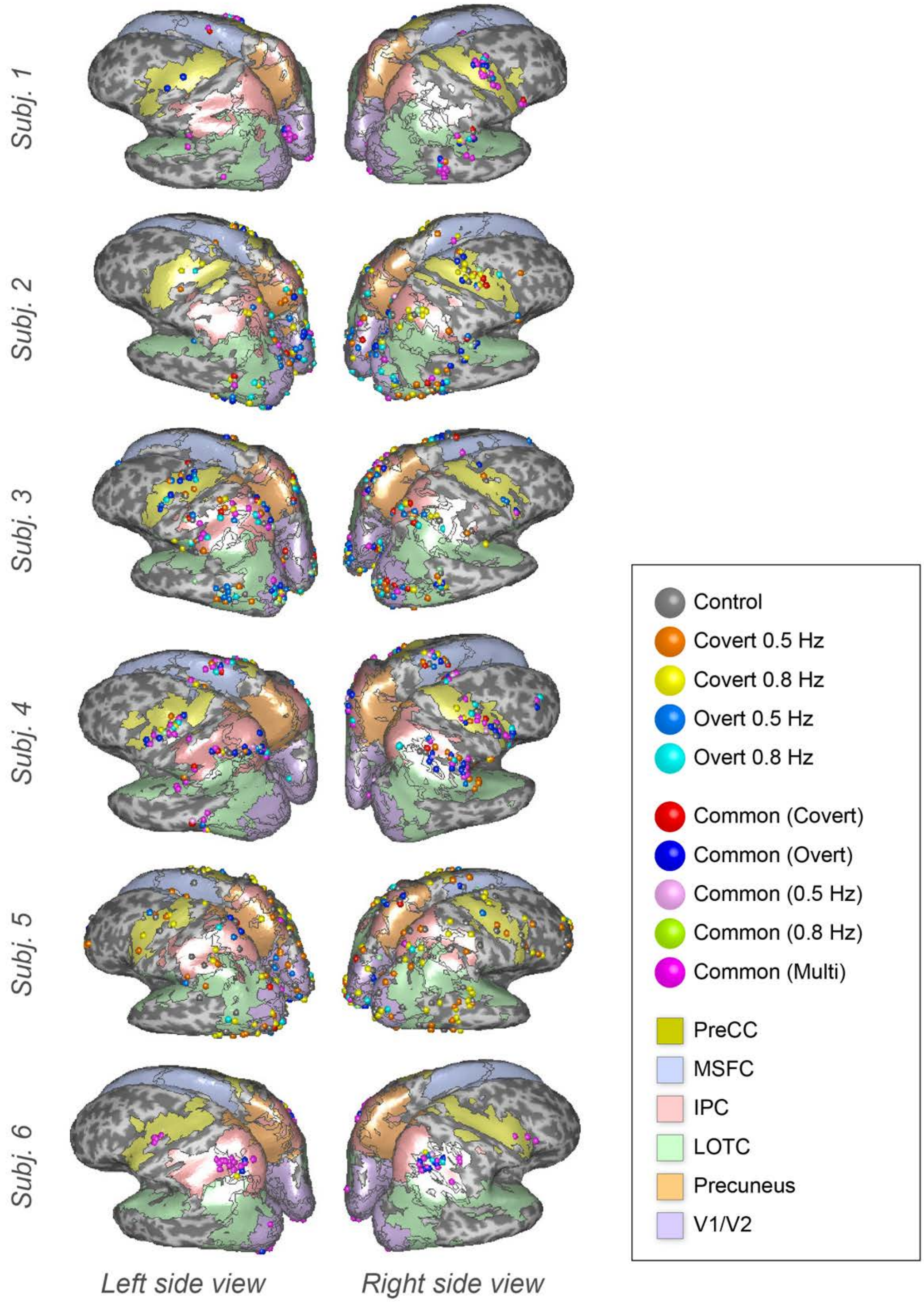

Figure S15. Selected dipole locations of all subjects (position)

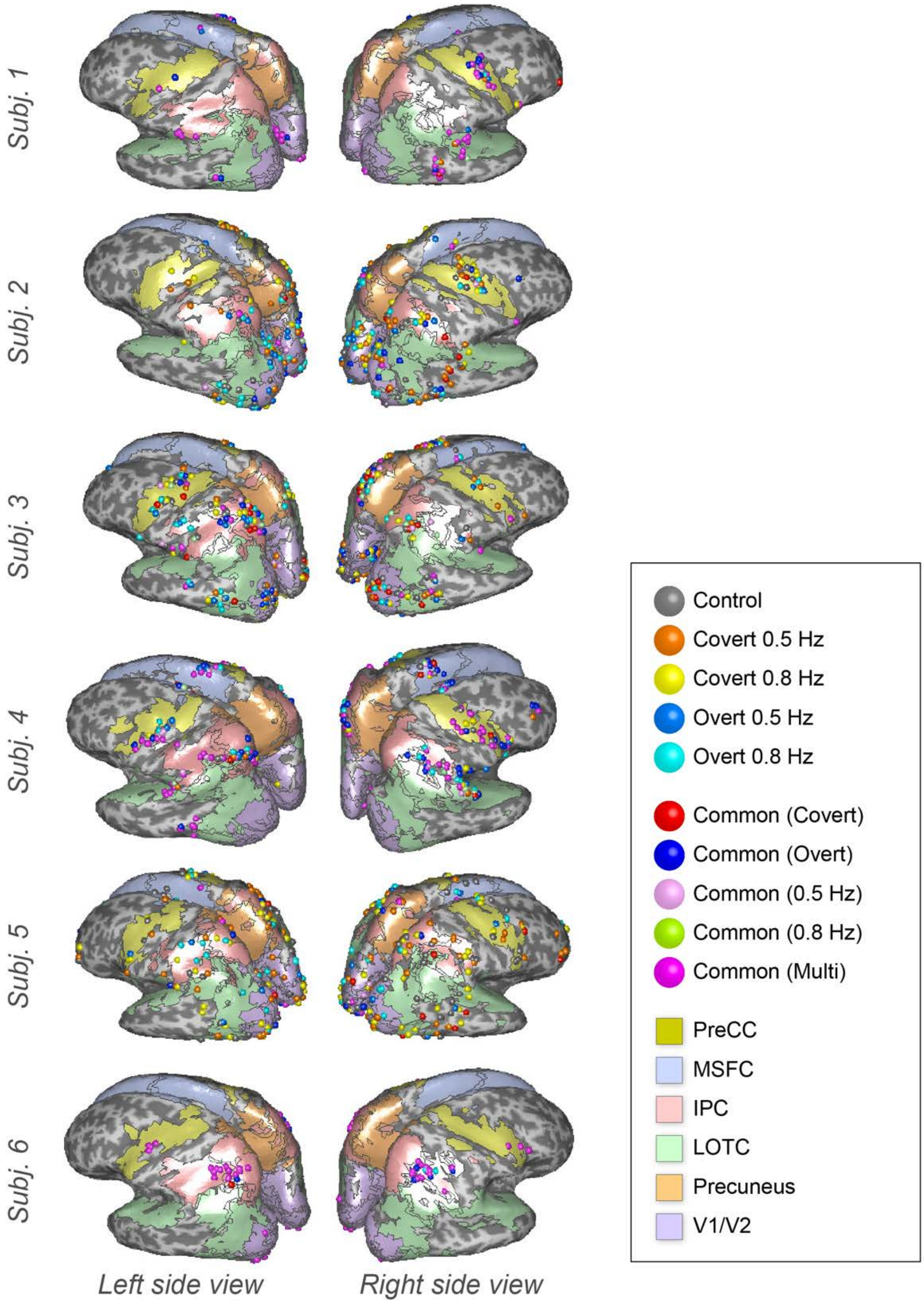

Figure S16. Selected dipole locations of all subjects (velocity)

6 Full results of randomized block design two-way ANOVA

6.1 Results of randomized block design two-way ANOVA (position, correlation coefficients)

We show full results of randomized block design two-way ANOVA on correlation coefficients for reconstructing time series of positional data (Tables S5 – S17).

Table S5. ANOVA summary table (position; correlation coefficients: *r*)

Source of variation	Sum of Squares	Degree of Freedom	Mean Square	*F*-Value	Prob > *F*
Subject: *S*	0.9887	*n*-1=5	0.1977		
Training task (A)					
Main effect: *A*	0.0106	*p*-1=4	0.0026	0.0367	0.9972
Error: *A*×*S*	1.4415	(*p*-1)(*n*-1)=20	0.0721		
Test task (B)					
Main effect: *B*	1.0042	*q*-1=4	0.2511	4.8568*	0.0067
Error: *B*×*S*	1.0338	(*q*-1)(*n*-1)=20	0.0517		
Interaction effect					
Interaction effect: *A*×*B*	6.3182	(*p*-1)(*q*-1)=3	0.3949	8.6983*	< 0.0001
Error: *A*×*B*×*S*	3.6318	(*p*-1)(*q*-1)(*n*-1)=80	0.0454		
Total: *T*	14.4289	*npq*-1=149			

* $p \leqq 0.05$

Table S6. Multiple comparison (position; correlation coefficients: *r*)

Main effect (Tukey HSD)

Training task (A)	Test task (B)	Mean difference	Standard error	*P*-Value
Main effect B				
Covert 0.8 Hz	Overt 0.5 Hz	0.1972	0.0415	0.0231

Table S7. Simple main effect test (position; correlation coefficients: *r*)

Interaction Effects

Simple main effect	Sum of Squares	Degree of Freedom	Mean Square	*F*-Value	Prob > F
A(Control)	1.3240	4	0.3310	6.5243*	0.0001
A(Covert 0.5 Hz)	1.3436	4	0.3359	6.6209*	< 0.0001
A(Covert 0.8 Hz)	1.0343	4	0.2586	5.0965*	0.0009
A(Overt 0.5 Hz)	1.1068	4	0.2767	5.4538*	0.0005
A(Overt 0.8 Hz)	1.5201	4	0.3800	7.4904*	< 0.0001
Error	$MS_{\text{pool(A)}}$	100	0.0507		
B(Control)	0.9918	4	0.2479	5.3143*	0.0006
B(Covert 0.5 Hz)	0.7195	4	0.1799	3.8552*	0.0059
B(Covert 0.8 Hz)	0.9603	4	0.2401	5.1453*	0.0008
B(Overt 0.5 Hz)	2.1706	4	0.5426	11.6305*	< 0.0001
B(Overt 0.8 Hz)	2.4803	4	0.6201	13.2902*	< 0.0001
Error	$MS_{\text{pool(B)}}$	100	0.0467		

* $p \leqq 0.05$

Table S8. Multiple comparison for the simple main effect test

[position; correlation coefficients: *r*; Training (A) - Test (Control)]

A(Control)

		Training (A)				
		Control	Covert 0.5 Hz	Covert 0.8 Hz	Overt 0.5 Hz	Overt 0.8 Hz
Training (A)	Test (B)	Test (B): Control				
Control	Control	–	0.0401	0.4163*	0.4584*	0.4654*
Covert 0.5 Hz		–	–	0.3762*	0.4183*	0.4253*
Covert 0.8 Hz		–	–	–	0.0421	0.0491
Overt 0.5 Hz		–	–	–	–	0.0070
Overt 0.8 Hz		–	–	–	–	–

HSD = 0.3704, $p \leqq 0.05$

Table S9. Multiple comparison for the simple main effect test

[position; correlation coefficients: *r*; Training (A) ‐ Test (Covert 0.5 Hz)]

A(Covert 0.5 Hz)

		Training (A)				
		Control	Covert 0.5 Hz	Covert 0.8 Hz	Overt 0.5 Hz	Overt 0.8 Hz
Training (A)	Test (B)	Test (B): Covert 0.5 Hz				
Control	Covert 0.5 Hz	–	0.0373	0.3385	0.3649	0.4939*
Covert 0.5 Hz		–	–	0.3758*	0.4022*	0.5312*
Covert 0.8 Hz		–	–	–	0.0265	0.1554
Overt 0.5 Hz		–	–	–	–	0.1290
Overt 0.8 Hz		–	–	–	–	–

HSD = 0.3704, $p \leqq 0.05$

Table S10. Multiple comparison for the simple main effect test

[position; correlation coefficients: *r*; Training (A) ‐ Test (Covert 0.8 Hz)]

A(Covert 0.8 Hz)

		Training (A)				
		Control	Covert 0.5 Hz	Covert 0.8 Hz	Overt 0.5 Hz	Overt 0.8 Hz
Training (A)	Test (B)	Test (B): Covert 0.8 Hz				
Control	Covert 0.8 Hz	–	0.0093	0.4642*	0.0424	0.0646
Covert 0.5 Hz		–	–	0.4549*	0.0517	0.0553
Covert 0.8 Hz		–	–	–	0.5066*	0.3995*
Overt 0.5 Hz		–	–	–	–	0.1070
Overt 0.8 Hz		–	–	–	–	–

HSD = 0.3704, $p \leqq 0.05$

Table S11. Multiple comparison for the simple main effect test

[position; correlation coefficients: *r*; Training (A) ‐ Test (Overt 0.5 Hz)]

A(Overt 0.5 Hz)

		Training (A)				
		Control	Covert 0.5 Hz	Covert 0.8 Hz	Overt 0.5 Hz	Overt 0.8 Hz
Training (A)	Test (B)	Test (B): Overt 0.5 Hz				
Control	Overt 0.5 Hz	–	0.0039	0.0361	0.3997*	0.3538
Covert 0.5 Hz		–	–	0.0323	0.4036*	0.3577
Covert 0.8 Hz		–	–	–	0.4359*	0.3900*
Overt 0.5 Hz		–	–	–	–	0.0459
Overt 0.8 Hz		–	–	–	–	–

HSD = 0.3704, $p \leqq 0.05$

Table S12. Multiple comparison for the simple main effect test
[position; correlation coefficients: *r*; Training (A) ‐ Test (Overt 0.8 Hz)]

A(Overt 0.8 Hz)

		Training (A)				
		Control	Covert 0.5 Hz	Covert 0.8 Hz	Overt 0.5 Hz	Overt 0.8 Hz
Training (A)	Test (B)	Test (B): Overt 0.8 Hz				
Control	Overt 0.8 Hz	–	0.1176	0.3386	0.5123*	0.5901*
Covert 0.5 Hz		–	–	0.2210	0.3947*	0.4726*
Covert 0.8 Hz		–	–	–	0.1737	0.2516
Overt 0.5 Hz		–	–	–	–	0.0778
Overt 0.8 Hz		–	–	–	–	–

HSD = 0.3704, $p \leqq 0.05$

Table S13. Multiple comparison for the simple main effect test
[position; correlation coefficients: *r*; Training (Control) - Test (B)]

B(Control)

		Training (A): Control				
		Test (B)				
Training (A)	Test (B)	Control	Covert 0.5 Hz	Covert 0.8 Hz	Overt 0.5 Hz	Overt 0.8 Hz
Control	Control	–	0.0530	0.4085*	0.2548	0.4506*
	Covert 0.5 Hz	–	–	0.3555*	0.2018	0.3976*
	Covert 0.8 Hz	–	–	–	0.1536	0.0421
	Overt 0.5 Hz	–	–	–	–	0.1957
	Overt 0.8 Hz	–	–	–	–	–

HSD = 0.3536, $p \leqq 0.05$

Table S14. Multiple comparison for the simple main effect test
[position; correlation coefficients: *r*; Training (Covert 0.5 Hz) ‐ Test (B)]

B(Covert 0.5 Hz)

		Training (A): Covert 0.5 Hz				
		Test (B)				
Training (A)	Test (B)	Control	Covert 0.5 Hz	Covert 0.8 Hz	Overt 0.5 Hz	Overt 0.8 Hz
Covert 0.5 Hz	Control	–	0.0244	0.3591*	0.2186	0.2929
	Covert 0.5 Hz	–	–	0.3835*	0.2430	0.3173
	Covert 0.8 Hz	–	–	–	0.1405	0.0662
	Overt 0.5 Hz	–	–	–	–	0.0743
	Overt 0.8 Hz	–	–	–	–	–

HSD = 0.3536, $p \leqq 0.05$

Table S15. Multiple comparison for the simple main effect test

[position; correlation coefficients: *r*; Training (Covert 0.8 Hz) - Test (B)]

B(Covert 0.8 Hz)

		Training (A): Covert 0.8 Hz				
		Test (B)				
Training (A)	Test (B)	Control	Covert 0.5 Hz	Covert 0.8 Hz	Overt 0.5 Hz	Overt 0.8 Hz
Covert 0.8 Hz	Control	–	0.0248	0.4720*	0.1253	0.3043
	Covert 0.5 Hz	–	–	0.4472*	0.1005	0.2795
	Covert 0.8 Hz	–	–	–	0.3467	0.1677
	Overt 0.5 Hz	–	–	–	–	0.1790
	Overt 0.8 Hz	–	–	–	–	–

HSD = 0.3536, $p \leqq 0.05$

Table S16. Multiple comparison for the simple main effect test

[position; correlation coefficients: *r*; Training (Overt 0.5 Hz) - Test (B)]

B(Overt 0.5 Hz)

		Training (A): Overt 0.5 Hz				
		Test (B)				
Training (A)	Test (B)	Control	Covert 0.5 Hz	Covert 0.8 Hz	Overt 0.5 Hz	Overt 0.8 Hz
Overt 0.5 Hz	Control	–	0.0405	0.0075	0.6033*	0.5201*
	Covert 0.5 Hz	–	–	0.0330	0.5628*	0.4797*
	Covert 0.8 Hz	–	–	–	0.5958*	0.5126*
	Overt 0.5 Hz	–	–	–	–	0.0831
	Overt 0.8 Hz	–	–	–	–	–

HSD = 0.3536, $p \leqq 0.05$

Table S17. Multiple comparison for the simple main effect test

[position; correlation coefficients: *r*; Training (Overt 0.8 Hz) - Test (B)]

B(Overt 0.8 Hz)

		Training (A): Overt 0.8 Hz				
		Test (B)				
Training (A)	Test (B)	Control	Covert 0.5 Hz	Covert 0.8 Hz	Overt 0.5 Hz	Overt 0.8 Hz
Overt 0.8 Hz	Control	–	0.0815	0.1215	0.5644*	0.6049*
	Covert 0.5 Hz	–	–	0.2031	0.6459*	0.6865*
	Covert 0.8 Hz	–	–	–	0.4428*	0.4834*
	Overt 0.5 Hz	–	–	–	–	0.0406
	Overt 0.8 Hz	–	–	–	–	–

HSD = 0.3536, $p \leqq 0.05$

6.2 Results of randomized block design two-way ANOVA (position, determination coefficients)

We show full results of randomized block design two-way ANOVA on determination coefficients for reconstructing time series of positional data (Tables S18 – S24).

Table S18. ANOVA summary table (position; determination coefficients: R^2)

Source	Sum of Squares	Degree of Freedom	Mean Square	F-Value	Prob > F
Subject (S)	0.3601	n-1=5	0.0720		
Training task (A)					
Training (A)	0.6290	p-1=4	0.1572	2.7292	0.0581
Error (A×S)	1.1523	(p-1)(n-1)=20	0.0576		
Test task (B)					
Test (B)	1.9326	q-1=4	0.4832	14.0756*	< 0.0001
Error (B×S)	0.6865	(q-1)(n-1)=20	0.0343		
Interaction effect					
Interaction (A×B)	5.1463	(p-1)(q-1)=3	0.3216	6.5643*	< 0.0001
Error (A×B×S)	3.9199	(p-1)(q-1)(n-1)=80	0.0490		
Total (T)	14.4289	npq-1=149			

* $p \leqq 0.05$

Table S19. Multiple comparison (position; determination coefficients: R^2)

Main effect (Tukey HSD)

Training task (A)	Test task (B)	Mean difference	Standard error	P-Value
Main effect B				
Control	Overt 0.5 Hz	0.2450	0.0338	0.0004
Control	Overt 0.8 Hz	0.2201	0.0338	0.0015
Covert 0.5 Hz	Overt 0.5 Hz	0.2535	0.0338	0.0003
Covert 0.5 Hz	Overt 0.8 Hz	0.2285	0.0338	0.0010
Covert 0.8 Hz	Overt 0.5 Hz	0.2309	0.0338	0.0009
Covert 0.8 Hz	Overt 0.8 Hz	0.2059	0.0338	0.0028

Table S20. Simple main effect test (position; determination coefficients: R^2)

Interaction Effects

Simple main effect	Sum of Squares	Degree of Freedom	Mean Square	*F*-Value	Prob > F
A(Control)	0.2486	4	0.0621	1.2251	0.3050
A(Covert 0.5 Hz)	0.1994	4	0.0499	0.9828	0.4205
A(Covert 0.8 Hz)	0.2037	4	0.0509	1.0040	0.4092
A(Overt 0.5 Hz)	3.0211	4	0.7553	14.8903*	< 0.0001
A(Overt 0.8 Hz)	2.1026	4	0.5256	10.3631*	< 0.0001
Error	$MS_{\text{pool(A)}}$	100	0.0507		
B(Control)	0.0309	4	0.0077	0.1680	0.9542
B(Covert 0.5 Hz)	0.0904	4	0.0226	0.4906	0.7426
B(Covert 0.8 Hz)	0.2505	4	0.0626	1.3593	0.2535
B(Overt 0.5 Hz)	3.3923	4	0.8481	18.4108*	< 0.0001
B(Overt 0.8 Hz)	3.3148	4	0.8287	17.9901*	< 0.0001
Error	$MS_{\text{pool(B)}}$	100	0.0461		

* $p \leqq 0.05$

Table S21. Multiple comparison for the simple main effect test [position; determination coefficients: R^2; Training (A) - Test (Overt 0.5 Hz)]

A(Overt 0.5 Hz)

		Training (A)				
		Control	Covert 0.5 Hz	Covert 0.8 Hz	Overt 0.5 Hz	Overt 0.8 Hz
Training (A)	Test (B)	Test (B): Overt 0.5 Hz				
Control	Overt 0.5 Hz	–	0.0066	0.0330	0.7766*	0.3935*
Covert 0.5 Hz		–	–	0.0264	0.7832*	0.4001*
Covert 0.8 Hz		–	–	–	0.8096*	0.4265*
Overt 0.5 Hz		–	–	–	–	0.3831*
Overt 0.8 Hz		–	–	–	–	–

HSD = 0.3689, $p \leqq 0.05$

Table S22. Multiple comparison for the simple main effect test

[position; determination coefficients: R^2; Training (A) - Test (Overt 0.8 Hz)]

A(Overt 0.8 Hz)

		Training (A)				
		Control	Covert 0.5 Hz	Covert 0.8 Hz	Overt 0.5 Hz	Overt 0.8 Hz
Training (A)	Test (B)	Test (B): Overt 0.8 Hz				
Control	Overt 0.8 Hz	–	0.0517	0.0658	0.1904	0.6812*
Covert 0.5 Hz		–	–	0.1174	0.2421	0.7329*
Covert 0.8 Hz		–	–	–	0.1246	0.6155*
Overt 0.5 Hz		–	–	–	–	0.4908*
Overt 0.8 Hz		–	–	–	–	–

HSD = 0.3689, $p \leqq 0.05$

Table S23. Multiple comparison for the simple main effect test

[position; determination coefficients: R^2; Training (Overt 0.5 Hz) - Test (B)]

B(Overt 0.5 Hz)

		Training (A): Overt 0.5 Hz				
		Test (B)				
Training (A)	Test (B)	Control	Covert 0.5 Hz	Covert 0.8 Hz	Overt 0.5 Hz	Overt 0.8 Hz
Overt 0.5 Hz	Control	–	0.0112	0.0042	0.8660*	0.3037
	Covert 0.5 Hz	–	–	0.0155	0.8547*	0.2925
	Covert 0.8 Hz	–	–	–	0.8702*	0.3080
	Overt 0.5 Hz	–	–	–	–	0.5622*
	Overt 0.8 Hz	–	–	–	–	–

HSD = 0.3496, $p \leqq 0.05$

Table S24. Multiple comparison for the simple main effect test

[position; determination coefficients: R^2; Training (Overt 0.8 Hz) - Test (B)]

B(Overt 0.8 Hz)

		Training (A): Overt 0.8 Hz				
		Test (B)				
Training (A)	Test (B)	Control	Covert 0.5 Hz	Covert 0.8 Hz	Overt 0.5 Hz	Overt 0.8 Hz
Overt 0.8 Hz	Control	–	0.0096	0.0391	0.5011*	0.8128*
	Covert 0.5 Hz	–	–	0.0488	0.5108*	0.8225*
	Covert 0.8 Hz	–	–	–	0.4620*	0.7737*
	Overt 0.5 Hz	–	–	–	–	0.3117
	Overt 0.8 Hz	–	–	–	–	–

HSD = 0.3496, $p \leqq 0.05$

6.3 Results of randomized block design two-way ANOVA (velocity, correlation coefficients)

We show full results of randomized block design two-way ANOVA on correlation coefficients for reconstructing time series of velocity data (Tables S25 – S36).

Table S25. ANOVA summary table (velocity; correlation coefficients: *r*)

Source	Sum of Squares	Degree of Freedom	Mean Square	*F*-Value	Prob > *F*
Subject (*S*)	0.3800	n-1=5	0.0760		
Training task (A)					
Training (*A*)	0.0623	p-1=4	0.0156	0.4566	0.7665
Error (*A*×*S*)	0.6819	(p-1)(n-1)=20	0.0341		
Test task (B)					
Test (*B*)	0.0151	q-1=4	0.0038	0.0817	0.9871
Error (*B*×*S*)	0.9249	(q-1)(n-1)=20	0.0462		
Interaction effect					
Interaction (*A*×*B*)	8.4300	(p-1)(q-1)=3	0.5269	20.8561*	< 0.0001
Error (*A*×*B*×*S*)	2.0210	(p-1)(q-1)(n-1)=80	0.0253		
Total (*T*)	14.4289	npq-1=149			

* $p \leqq 0.05$

Table S26. Simple main effect test (velocity; correlation coefficients: *r*)

Interaction Effects

Simple main effect	Sum of Squares	Degree of Freedom	Mean Square	*F*-Value	Prob > F
A(Control)	1.7203	4	0.4301	15.9118*	< 0.0001
A(Covert 0.5 Hz)	1.6598	4	0.4150	15.3524*	< 0.0001
A(Covert 0.8 Hz)	0.9160	4	0.2290	8.4724*	< 0.0001
A(Overt 0.5 Hz)	1.4963	4	0.3741	13.8400*	< 0.0001
A(Overt 0.8 Hz)	2.6998	4	0.6749	24.9711*	< 0.0001
Error	$MS_{\text{pool(A)}}$	100	0.0270		
B(Control)	1.3905	4	0.3476	11.8001*	< 0.0001
B(Covert 0.5 Hz)	2.1645	4	0.5411	18.3692*	< 0.0001
B(Covert 0.8 Hz)	0.6717	4	0.1679	5.7003*	0.0004
B(Overt 0.5 Hz)	2.1051	4	0.5263	17.8646*	< 0.0001
B(Overt 0.8 Hz)	2.1133	4	0.5283	17.9345*	< 0.0001
Error	$MS_{\text{pool(B)}}$	100	0.0295		

* $p \leqq 0.05$

Table S27. Multiple comparison for the simple main effect test

[velocity; correlation coefficients: *r*; Training (A) - Test (Control)]

A(Control)

		Training (A)				
		Control	Covert 0.5 Hz	Covert 0.8 Hz	Overt 0.5 Hz	Overt 0.8 Hz
Training (A)	Test (B)	Test (B): Control				
Control	Control	–	0.0425	0.3497*	0.5268*	0.5766*
Covert 0.5 Hz		–	–	0.3072*	0.4843*	0.5341*
Covert 0.8 Hz		–	–	–	0.1771	0.2269
Overt 0.5 Hz		–	–	–	–	0.0498
Overt 0.8 Hz		–	–	–	–	–

HSD = 0.2697, $p \leqq 0.05$

Table S28. Multiple comparison for the simple main effect test

[velocity; correlation coefficients: *r*; Training (A) - Test (Covert 0.5 Hz)]

A(Covert 0.5 Hz)

		Training (A)				
		Control	Covert 0.5 Hz	Covert 0.8 Hz	Overt 0.5 Hz	Overt 0.8 Hz
Training (A)	Test (B)	Test (B): Covert 0.5 Hz				
Control	Covert 0.5 Hz	–	0.0301	0.3375*	0.4412*	0.5533*
Covert 0.5 Hz		–	–	0.3677*	0.4714*	0.5834*
Covert 0.8 Hz		–	–	–	0.1037	0.2157
Overt 0.5 Hz		–	–	–	–	0.1120
Overt 0.8 Hz		–	–	–	–	–

HSD = 0.2697, $p \leqq 0.05$

Table S29. Multiple comparison for the simple main effect test

[velocity; correlation coefficients: *r*; Training (A) - Test (Covert 0.8 Hz)]

A(Covert 0.8 Hz)

		Training (A)				
		Control	Covert 0.5 Hz	Covert 0.8 Hz	Overt 0.5 Hz	Overt 0.8 Hz
Training (A)	Test (B)	Test (B): Covert 0.8 Hz				
Control	Covert 0.8 Hz	–	0.0054	0.2446	0.2864*	0.1309
Covert 0.5 Hz		–	–	0.2501	0.2809*	0.1255
Covert 0.8 Hz		–	–	–	0.5310*	0.3755*
Overt 0.5 Hz		–	–	–	–	0.1555
Overt 0.8 Hz		–	–	–	–	–

HSD = 0.2697, $p \leqq 0.05$

Table S30. Multiple comparison for the simple main effect test

[velocity; correlation coefficients: *r*; Training (A) - Test (Overt 0.5 Hz)]

A(Overt 0.5 Hz)

		Training (A)				
		Control	Covert 0.5 Hz	Covert 0.8 Hz	Overt 0.5 Hz	Overt 0.8 Hz
Training (A)	Test (B)	Test (B): Overt 0.5 Hz				
Control	Overt 0.5 Hz	–	0.0591	0.0104	0.5031*	0.3311*
Covert 0.5 Hz		–	–	0.0487	0.5622*	0.3902*
Covert 0.8 Hz		–	–	–	0.5135*	0.3414*
Overt 0.5 Hz		–	–	–	–	0.1720
Overt 0.8 Hz		–	–	–	–	–

HSD = 0.2697, $p \leqq 0.05$

Table S31. Multiple comparison for the simple main effect test

[velocity; correlation coefficients: *r*; Training (A) - Test (Overt 0.8 Hz)]

A(Overt 0.8 Hz)

		Training (A)				
		Control	Covert 0.5 Hz	Covert 0.8 Hz	Overt 0.5 Hz	Overt 0.8 Hz
Training (A)	Test (B)	Test (B): Overt 0.8 Hz				
Control	Overt 0.8 Hz	–	0.1606	0.2392	0.5180*	0.6334*
Covert 0.5 Hz		–	–	0.3997*	0.6786*	0.7940*
Covert 0.8 Hz		–	–	–	0.2789*	0.3943*
Overt 0.5 Hz		–	–	–	–	0.1154
Overt 0.8 Hz		–	–	–	–	–

HSD = 0.2697, $p \leqq 0.05$

Table S32. Multiple comparison for the simple main effect test

[velocity; correlation coefficients: *r*; Training (Control) - Test (B)]

B(Control)

		Training (A): Control				
		Test (B)				
Training (A)	Test (B)	Control	Covert 0.5 Hz	Covert 0.8 Hz	Overt 0.5 Hz	Overt 0.8 Hz
Control	Control	–	0.0307	0.2516	0.4275*	0.5494*
	Covert 0.5 Hz	–	–	0.2209	0.3968*	0.5187*
	Covert 0.8 Hz	–	–	–	0.1759	0.2978*
	Overt 0.5 Hz	–	–	–	–	0.1219
	Overt 0.8 Hz	–	–	–	–	–

HSD = 0.2828, $p \leqq 0.05$

Table S33. Multiple comparison for the simple main effect test

[velocity; correlation coefficients: *r*; Training (Covert 0.5 Hz) - Test (B)]

B(Covert 0.5 Hz)

		Training (A): Covert 0.5 Hz				
		Test (B)				
Training (A)	Test (B)	Control	Covert 0.5 Hz	Covert 0.8 Hz	Overt 0.5 Hz	Overt 0.8 Hz
Covert 0.5 Hz	Control	–	0.0419	0.2146	0.4442*	0.6675*
	Covert 0.5 Hz	–	–	0.2565	0.4861*	0.7094*
	Covert 0.8 Hz	–	–	–	0.2296	0.4530*
	Overt 0.5 Hz	–	–	–	–	0.2234
	Overt 0.8 Hz	–	–	–	–	–

HSD = 0.2828, $p \leqq 0.05$

Table S34. Multiple comparison for the simple main effect test

[velocity; correlation coefficients: *r*; Training (Covert 0.8 Hz) - Test (B)]

B(Covert 0.8 Hz)

		Training (A): Covert 0.8 Hz				
		Test (B)				
Training (A)	Test (B)	Control	Covert 0.5 Hz	Covert 0.8 Hz	Overt 0.5 Hz	Overt 0.8 Hz
Covert 0.8 Hz	Control	–	0.0186	0.3427*	0.0882	0.0394
	Covert 0.5 Hz	–	–	0.3612*	0.0696	0.0580
	Covert 0.8 Hz	–	–	–	0.4309*	0.3033*
	Overt 0.5 Hz	–	–	–	–	0.1276
	Overt 0.8 Hz	–	–	–	–	–

HSD = 0.2828, $p \leqq 0.05$

Table S35. Multiple comparison for the simple main effect test

[velocity; correlation coefficients: *r*; Training (Overt 0.5 Hz) - Test (B)]

B(Overt 0.5 Hz)

		Training (A): Overt 0.5 Hz				
		Test (B)				
Training (A)	Test (B)	Control	Covert 0.5 Hz	Covert 0.8 Hz	Overt 0.5 Hz	Overt 0.8 Hz
Overt 0.5 Hz	Control	–	0.0549	0.0112	0.6024*	0.4954*
	Covert 0.5 Hz	–	–	0.0660	0.5475*	0.4405*
	Covert 0.8 Hz	–	–	–	0.6136*	0.5066*
	Overt 0.5 Hz	–	–	–	–	0.1070
	Overt 0.8 Hz	–	–	–	–	–

HSD = 0.2828, $p \leqq 0.05$

Table S36. Multiple comparison for the simple main effect test

[velocity; correlation coefficients: *r*; Training (Overt 0.8 Hz) - Test (B)]

B(Overt 0.8 Hz)

		Training (A): Overt 0.8 Hz				
		Test (B)				
Training (A)	Test (B)	Control	Covert 0.5 Hz	Covert 0.8 Hz	Overt 0.5 Hz	Overt 0.8 Hz
Overt 0.8 Hz	Control	–	0.0074	0.1940	0.4801*	0.6606*
	Covert 0.5 Hz	–	–	0.2015	0.4875*	0.6680*
	Covert 0.8 Hz	–	–	–	0.2861*	0.4665*
	Overt 0.5 Hz	–	–	–	–	0.1804
	Overt 0.8 Hz	–	–	–	–	–

$HSD = 0.2828, p \leqq 0.05$

6.4 Results of randomized block design two-way ANOVA (velocity, determination coefficients)

We show full results of randomized block design two-way ANOVA on determination coefficients for reconstructing time series of velocity data (Tables S37 – S49).

Table S37. ANOVA summary table (velocity; determination coefficients: R^2)

Source	Sum of Squares	Degree of Freedom	Mean Square	F-Value	Prob > F
Subject (S)	0.2003	n-1=5	0.0401		
Training task (A)					
Training (A)	0.7563	p-1=4	0.1891	11.6167*	< 0.0001
Error (A×S)	0.3255	(p-1)(n-1)=20	0.0163		
Test task (B)					
Test (B)	0.8688	q-1=4	0.2172	26.2520*	< 0.0001
Error (B×S)	0.1655	(q-1)(n-1)=20	0.0083		
Interaction effect					
Interaction (A×B)	6.2559	(p-1)(q-1)=3	0.3910	36.0023*	< 0.0001
Error (A×B×S)	0.8688	(p-1)(q-1)(n-1)=80	0.0109		
Total (T)	9.4411	npq-1=149			

* $p \leqq 0.05$

Table S38. Multiple comparison (velocity; determination coefficients: R^2)

Main effect (Tukey HSD)

Training task (A)	Test task (B)	Mean difference	Standard error	P-Value
Main effect A				
Control	Overt 0.5 Hz	0.1500	0.0233	0.0016
Control	Overt 0.8 Hz	0.1335	0.0233	0.0050
Covert 0.5 Hz	Overt 0.5 Hz	0.1698	0.0233	0.0004
Covert 0.5 Hz	Overt 0.8 Hz	0.1533	0.0233	0.0013
Covert 0.8 Hz	Overt 0.5 Hz	0.1326	0.0233	0.0053
Covert 0.8 Hz	Overt 0.8 Hz	0.1161	0.0233	0.0161
Main effect B				
Control	Overt 0.5 Hz	0.1557	0.0166	< 0.0001
Control	Overt 0.8 Hz	0.1458	0.0166	< 0.0001
Covert 0.5 Hz	Overt 0.5 Hz	0.1533	0.0166	< 0.0001
Covert 0.5 Hz	Overt 0.8 Hz	0.1434	0.0166	< 0.0001
Covert 0.8 Hz	Overt 0.5 Hz	0.1702	0.0166	< 0.0001
Covert 0.8 Hz	Overt 0.8 Hz	0.1603	0.0166	< 0.0001

Table S39. Simple main effect test (velocity; determination coefficients: R^2)

Interaction Effects

Simple main effect	Sum of Squares	Degree of Freedom	Mean Square	F-Value	Prob > F
A(Control)	0.3368	4	0.0842	7.0488*	< 0.0001
A(Covert 0.5 Hz)	0.3273	4	0.0818	6.8517*	< 0.0001
A(Covert 0.8 Hz)	0.2553	4	0.0638	5.3436*	0.0006
A(Overt 0.5 Hz)	2.5103	4	0.6276	52.5441*	< 0.0001
A(Overt 0.8 Hz)	3.5826	4	0.8957	74.9901*	< 0.0001
Error	$MS_{\text{pool(A)}}$	100	0.0119		
B(Control)	0.1559	4	0.0390	3.7695*	0.0067
B(Covert 0.5 Hz)	0.7616	4	0.1904	18.4078*	< 0.0001
B(Covert 0.8 Hz)	0.1328	4	0.0332	3.2096*	0.0159
B(Overt 0.5 Hz)	3.1493	4	0.7873	76.1222*	< 0.0001
B(Overt 0.8 Hz)	2.9251	4	0.7313	70.7030*	< 0.0001
Error	$MS_{\text{pool(B)}}$	100	0.0103		

* $p \leqq 0.05$

Table S40. Multiple comparison for the simple main effect test

[velocity; determination coefficients: R^2; Training (A) - Test (Control)]

A(Control)

		Training (A)				
		Control	Covert 0.5 Hz	Covert 0.8 Hz	Overt 0.5 Hz	Overt 0.8 Hz
Training (A)	Test (B)	Test (B): Control				
Control	Control	–	0.0451	0.0950	0.2117*	0.2126*
Covert 0.5 Hz		–	–	0.1402	0.2569*	0.2578*
Covert 0.8 Hz		–	–	–	0.1167	0.1176
Overt 0.5 Hz		–	–	–	–	0.0009
Overt 0.8 Hz		–	–	–	–	–

$HSD = 0.1796, p \leqq 0.05$

Table S41. Multiple comparison for the simple main effect test

[velocity; determination coefficients: R^2; Training (A) - Test (Covert 0.5 Hz)]

A(Covert 0.5 Hz)

		Training (A)				
		Control	Covert 0.5 Hz	Covert 0.8 Hz	Overt 0.5 Hz	Overt 0.8 Hz
Training (A)	Test (B)	Test (B): Covert 0.5 Hz				
Control	Covert 0.5 Hz	–	0.0836	0.0742	0.1709	0.1960*
Covert 0.5 Hz		–	–	0.1577	0.2545*	0.2796*
Covert 0.8 Hz		–	–	–	0.0967	0.1219
Overt 0.5 Hz		–	–	–	–	0.0252
Overt 0.8 Hz		–	–	–	–	–

$HSD = 0.1796, p \leqq 0.05$

Table S42. Multiple comparison for the simple main effect test

[velocity; determination coefficients: R^2; Training (A) - Test (Covert 0.8 Hz)]

A(Covert 0.8 Hz)

		Training (A)				
		Control	Covert 0.5 Hz	Covert 0.8 Hz	Overt 0.5 Hz	Overt 0.8 Hz
Training (A)	Test (B)	Test (B): Covert 0.8 Hz				
Control	Covert 0.8 Hz	–	0.0229	0.1713	0.0932	0.0653
Covert 0.5 Hz		–	–	0.1484	0.1160	0.0882
Covert 0.8 Hz		–	–	–	0.2644*	0.2366*
Overt 0.5 Hz		–	–	–	–	0.0278
Overt 0.8 Hz		–	–	–	–	–

$HSD = 0.1796, p \leqq 0.05$

Table S43. Multiple comparison for the simple main effect test

[velocity; determination coefficients: R^2; Training (A) - Test (Overt 0.5 Hz)]

A(Overt 0.5 Hz)

		Training (A)				
		Control	Covert 0.5 Hz	Covert 0.8 Hz	Overt 0.5 Hz	Overt 0.8 Hz
Training (A)	Test (B)	Test (B): Overt 0.5 Hz				
Control	Overt 0.5 Hz	–	0.0875	0.0087	0.6668*	0.4030*
Covert 0.5 Hz		–	–	0.0962	0.7543*	0.4905*
Covert 0.8 Hz		–	–	–	0.6581*	0.3943*
Overt 0.5 Hz		–	–	–	–	0.2638*
Overt 0.8 Hz		–	–	–	–	–

HSD = 0.1796, $p \leqq 0.05$

Table S44. Multiple comparison for the simple main effect test

[velocity; determination coefficients: R^2; Training (A) - Test (Overt 0.8 Hz)]

A(Overt 0.8 Hz)

		Training (A)				
		Control	Covert 0.5 Hz	Covert 0.8 Hz	Overt 0.5 Hz	Overt 0.8 Hz
Training (A)	Test (B)	Test (B): Overt 0.8 Hz				
Control	Overt 0.8 Hz	–	0.1631	0.0761	0.5587*	0.7386*
Covert 0.5 Hz		–	–	0.2392*	0.7218*	0.9017*
Covert 0.8 Hz		–	–	–	0.4826*	0.6625*
Overt 0.5 Hz		–	–	–	–	0.1799*
Overt 0.8 Hz		–	–	–	–	–

HSD = 0.1796, $p \leqq 0.05$

Table S45. Multiple comparison for the simple main effect test

[velocity; determination coefficients: R^2; Training (Control) - Test (B)]

B(Control)

		Training (A): Control				
		Test (B)				
Training (A)	Test (B)	Control	Covert 0.5 Hz	Covert 0.8 Hz	Overt 0.5 Hz	Overt 0.8 Hz
Control	Control	–	0.0209	0.1165	0.1374	0.1911*
	Covert 0.5 Hz	–	–	0.0955	0.1165	0.1702*
	Covert 0.8 Hz	–	–	–	0.0209	0.0747
	Overt 0.5 Hz	–	–	–	–	0.0537
	Overt 0.8 Hz	–	–	–	–	–

HSD = 0.1658, $p \leqq 0.05$

Table S46. Multiple comparison for the simple main effect test
[velocity; determination coefficients: R^2; Training (Covert 0.5 Hz) - Test (B)]

B(Covert 0.5 Hz)

		Training (A): Covert 0.5 Hz				
		Test (B)				
Training (A)	Test (B)	Control	Covert 0.5 Hz	Covert 0.8 Hz	Overt 0.5 Hz	Overt 0.8 Hz
Covert 0.5 Hz	Control	–	0.0175	0.1387	0.2700*	0.3993*
	Covert 0.5 Hz	–	–	0.1563	0.2875*	0.4168*
	Covert 0.8 Hz	–	–	–	0.1313	0.2606*
	Overt 0.5 Hz	–	–	–	–	0.1293
	Overt 0.8 Hz	–	–	–	–	–

$HSD = 0.1658, p \leqq 0.05$

Table S47. Multiple comparison for the simple main effect test
[velocity; determination coefficients: R^2; Training (Covert 0.8 Hz) - Test (B)]

B(Covert 0.8 Hz)

		Training (A): Covert 0.8 Hz				
		Test (B)				
Training (A)	Test (B)	Control	Covert 0.5 Hz	Covert 0.8 Hz	Overt 0.5 Hz	Overt 0.8 Hz
Covert 0.8 Hz	Control	–	0.0001	0.1498	0.0336	0.0200
	Covert 0.5 Hz	–	–	0.1499	0.0336	0.0200
	Covert 0.8 Hz	–	–	–	0.1834*	0.1698*
	Overt 0.5 Hz	–	–	–	–	0.0136
	Overt 0.8 Hz	–	–	–	–	–

$HSD = 0.1658, p \leqq 0.05$

Table S48. Multiple comparison for the simple main effect test
[velocity; determination coefficients: R^2; Training (Overt 0.5 Hz) - Test (B)]

B(Overt 0.5 Hz)

		Training (A): Overt 0.5 Hz				
		Test (B)				
Training (A)	Test (B)	Control	Covert 0.5 Hz	Covert 0.8 Hz	Overt 0.5 Hz	Overt 0.8 Hz
Overt 0.5 Hz	Control	–	0.0199	0.0021	0.7412*	0.5793*
	Covert 0.5 Hz	–	–	0.0178	0.7213*	0.5594*
	Covert 0.8 Hz	–	–	–	0.7391*	0.5772*
	Overt 0.5 Hz	–	–	–	–	0.1619
	Overt 0.8 Hz	–	–	–	–	–

$HSD = 0.1658, p \leqq 0.05$

Table S49. Multiple comparison for the simple main effect test

[velocity; determination coefficients: R^2; Training (Overt 0.8 Hz) - Test (B)]

B(Overt 0.8 Hz)

		Training (A): Overt 0.8 Hz				
		Test (B)				
Training (A)	Test (B)	Control	Covert 0.5 Hz	Covert 0.8 Hz	Overt 0.5 Hz	Overt 0.8 Hz
Overt 0.8 Hz	Control	–	0.0043	0.0308	0.4783*	0.7601*
	Covert 0.5 Hz	–	–	0.0352	0.4826*	0.7645*
	Covert 0.8 Hz	–	–	–	0.4475*	0.7293*
	Overt 0.5 Hz	–	–	–	–	0.2818*
	Overt 0.8 Hz	–	–	–	–	–

$HSD = 0.1658, p \leqq 0.05$

7 Full results of randomized block design two-way ANOVA calculated from normalized indices

7.1 Results of randomized block design two-way ANOVA (position, normalized correlation coefficients)

We show full results of randomized block design two-way ANOVA on normalized correlation coefficients for reconstructing time series of positional data (Tables S50 – S61).

Table S50. ANOVA summary table (position; normalized correlation coefficients: *r*)

Source of variation	Sum of Squares	Degree of Freedom	Mean Square	F-Value	Prob > F
Subject: S	1.0467	n-1=5	0.2093		
Training task (A)					
Main effect: A	0.4005	p-1=4	0.1001	1.1033	0.3823
Error: A×S	1.8150	(p-1)(n-1)=20	0.0908		
Test task (B)					
Main effect: B	0.8915	q-1=4	0.2229	2.9530*	0.0455
Error: B×S	1.5094	(q-1)(n-1)=20	0.0755		
Interaction effect					
Interaction effect: A×B	7.7278	(p-1)(q-1)=3	0.4830	7.7753*	< 0.0001
Error: A×B×S	4.9695	(p-1)(q-1)(n-1)=80	0.0621		
Total: T	18.3604	npq-1=149			

* $p \leqq 0.05$

Table S51. Simple main effect test (position; normalized correlation coefficients: *r*)

Interaction Effects

Simple main effect	Sum of Squares	Degree of Freedom	Mean Square	*F*-Value	Prob > F
A(Control)	2.4025	4	0.6006	8.8529*	< 0.0001
A(Covert 0.5 Hz)	2.4150	4	0.6038	8.8991*	< 0.0001
A(Covert 0.8 Hz)	1.3464	4	0.3366	4.9614*	0.0011
A(Overt 0.5 Hz)	0.7573	4	0.1893	2.7904*	0.0303
A(Overt 0.8 Hz)	1.2071	4	0.3018	4.4481*	0.0024
Error	$MS_{\text{pool(A)}}$	100	0.0678		
B(Control)	1.4553	4	0.3638	5.6156*	0.0004
B(Covert 0.5 Hz)	1.1105	4	0.2776	4.2850*	0.0031
B(Covert 0.8 Hz)	1.1971	4	0.2993	4.6193*	0.0018
B(Overt 0.5 Hz)	2.2517	4	0.5629	8.6887*	< 0.0001
B(Overt 0.8 Hz)	2.6047	4	0.6512	10.0505*	< 0.0001
Error	$MS_{\text{pool(B)}}$	100	0.0648		

* $p \leqq 0.05$

Table S52. Multiple comparison for the simple main effect test

[position; normalized correlation coefficients: *r*; Training (A) - Test (Control)]

A(Control)

		Training (A)				
		Control	Covert 0.5 Hz	Covert 0.8 Hz	Overt 0.5 Hz	Overt 0.8 Hz
Training (A)	Test (B)	Test (B): Control				
Control	Control	–	0.0273	0.5290*	0.6137*	0.6193*
Covert 0.5 Hz		–	–	0.5017*	0.5864*	0.5920*
Covert 0.8 Hz		–	–	–	0.0848	0.0903
Overt 0.5 Hz		–	–	–	–	0.0055
Overt 0.8 Hz		–	–	–	–	–

$HSD = 0.4278, p \leqq 0.05$

Table S53. Multiple comparison for the simple main effect test

[position; normalized correlation coefficients: *r*; Training (A) - Test (Covert 0.5 Hz)]

A(Covert 0.5 Hz)

		Training (A)				
		Control	Covert 0.5 Hz	Covert 0.8 Hz	Overt 0.5 Hz	Overt 0.8 Hz
Training (A)	Test (B)	Test (B): Covert 0.5 Hz				
Control	Covert 0.5 Hz	–	0.0612	0.4391*	0.5129*	0.6429*
Covert 0.5 Hz		–	–	0.5003*	0.5741*	0.7041*
Covert 0.8 Hz		–	–	–	0.0738	0.2038
Overt 0.5 Hz		–	–	–	–	0.1300
Overt 0.8 Hz		–	–	–	–	–

HSD = 0.4278, $p \leqq 0.05$

Table S54. Multiple comparison for the simple main effect test

[position; normalized correlation coefficients: *r*; Training (A) - Test (Covert 0.8 Hz)]

A(Covert 0.8 Hz)

		Training (A)				
		Control	Covert 0.5 Hz	Covert 0.8 Hz	Overt 0.5 Hz	Overt 0.8 Hz
Training (A)	Test (B)	Test (B): Covert 0.8 Hz				
Control	Covert 0.8 Hz	–	0.0265	0.4930*	0.1143	0.0027
Covert 0.5 Hz		–	–	0.4665*	0.1408	0.0292
Covert 0.8 Hz		–	–	–	0.6073*	0.4956*
Overt 0.5 Hz		–	–	–	–	0.1117
Overt 0.8 Hz		–	–	–	–	–

HSD = 0.4278, $p \leqq 0.05$

Table S55. Multiple comparison for the simple main effect test

[position; normalized correlation coefficients: *r*; Training (A) - Test (Overt 0.5 Hz)]

A(Overt 0.5 Hz)

		Training (A)				
		Control	Covert 0.5 Hz	Covert 0.8 Hz	Overt 0.5 Hz	Overt 0.8 Hz
Training (A)	Test (B)	Test (B): Overt 0.5 Hz				
Control	Overt 0.5 Hz	–	0.0041	0.0720	0.3145	0.2727
Covert 0.5 Hz		–	–	0.0679	0.3186	0.2768
Covert 0.8 Hz		–	–	–	0.3865	0.3447
Overt 0.5 Hz		–	–	–	–	0.0418
Overt 0.8 Hz		–	–	–	–	–

HSD = 0.4278, $p \leqq 0.05$

Table S56. Multiple comparison for the simple main effect test

[position; normalized correlation coefficients: *r*; Training (A) - Test (Overt 0.8 Hz)]

A(Overt 0.8 Hz)

		Training (A)				
		Control	Covert 0.5 Hz	Covert 0.8 Hz	Overt 0.5 Hz	Overt 0.8 Hz
Training (A)	Test (B)	Test (B): Overt 0.8 Hz				
Control	Overt 0.8 Hz	–	0.1467	0.3527	0.4591*	0.5441*
Covert 0.5 Hz		–	–	0.2060	0.3124	0.3975
Covert 0.8 Hz		–	–	–	0.1064	0.1914
Overt 0.5 Hz		–	–	–	–	0.0851
Overt 0.8 Hz		–	–	–	–	–

HSD = 0.4278, $p \leqq 0.05$

Table S57. Multiple comparison for the simple main effect test

[position; normalized correlation coefficients: *r*; Training (Control) - Test (B)]

B(Control)

		Training (A): Control				
		Test (B)				
Training (A)	Test (B)	Control	Covert 0.5 Hz	Covert 0.8 Hz	Overt 0.5 Hz	Overt 0.8 Hz
Control	Control	–	0.0612	0.4930*	0.3145	0.5441*
	Covert 0.5 Hz	–	–	0.4318*	0.2533	0.4829*
	Covert 0.8 Hz	–	–	–	0.1785	0.0512
	Overt 0.5 Hz	–	–	–	–	0.2297
	Overt 0.8 Hz	–	–	–	–	–

HSD = 0.4170, $p \leqq 0.05$

Table S58. Multiple comparison for the simple main effect test

[position; normalized correlation coefficients: *r*; Training (Covert 0.5 Hz) - Test (B)]

B(Covert 0.5 Hz)

		Training (A): Covert 0.5 Hz				
		Test (B)				
Training (A)	Test (B)	Control	Covert 0.5 Hz	Covert 0.8 Hz	Overt 0.5 Hz	Overt 0.8 Hz
Covert 0.5 Hz	Control	–	0.0273	0.4392*	0.2913	0.3702
	Covert 0.5 Hz	–	–	0.4665*	0.3186	0.3975
	Covert 0.8 Hz	–	–	–	0.1479	0.0690
	Overt 0.5 Hz	–	–	–	–	0.0789
	Overt 0.8 Hz	–	–	–	–	–

HSD = 0.4170, $p \leqq 0.05$

Table S59. Multiple comparison for the simple main effect test

[position; normalized correlation coefficients: *r*; Training (Covert 0.8 Hz) – Test (B)]

B(Covert 0.8 Hz)

		Training (A): Covert 0.8 Hz				
		Test (B)				
Training (A)	Test (B)	Control	Covert 0.5 Hz	Covert 0.8 Hz	Overt 0.5 Hz	Overt 0.8 Hz
Covert 0.8 Hz	Control	–	0.0286	0.5290*	0.1425	0.3375
	Covert 0.5 Hz	–	–	0.5003*	0.1138	0.3089
	Covert 0.8 Hz	–	–	–	0.3865	0.1914
	Overt 0.5 Hz	–	–	–	–	0.1950
	Overt 0.8 Hz	–	–	–	–	–

HSD = 0.4170, $p \leqq 0.05$

Table S60. Multiple comparison for the simple main effect test

[position; normalized correlation coefficients: *r*; Training (Overt 0.5 Hz) - Test (B)]

B(Overt 0.5 Hz)

		Training (A): Overt 0.5 Hz				
		Test (B)				
Training (A)	Test (B)	Control	Covert 0.5 Hz	Covert 0.8 Hz	Overt 0.5 Hz	Overt 0.8 Hz
Overt 0.5 Hz	Control	–	0.0396	0.0064	0.6137*	0.5287*
	Covert 0.5 Hz	–	–	0.0332	0.5741*	0.4891*
	Covert 0.8 Hz	–	–	–	0.6073*	0.5222*
	Overt 0.5 Hz	–	–	–	–	0.0851
	Overt 0.8 Hz	–	–	–	–	–

HSD = 0.4170, $p \leqq 0.05$

Table S61. Multiple comparison for the simple main effect test

[position; normalized correlation coefficients: *r*; Training (Overt 0.8 Hz) - Test (B)]

B(Overt 0.8 Hz)

		Training (A): Overt 0.8 Hz				
		Test (B)				
Training (A)	Test (B)	Control	Covert 0.5 Hz	Covert 0.8 Hz	Overt 0.5 Hz	Overt 0.8 Hz
Overt 0.8 Hz	Control	–	0.0848	0.1236	0.5774*	0.6193*
	Covert 0.5 Hz	–	–	0.2084	0.6623*	0.7041*
	Covert 0.8 Hz	–	–	–	0.4538*	0.4956*
	Overt 0.5 Hz	–	–	–	–	0.0418
	Overt 0.8 Hz	–	–	–	–	–

HSD = 0.4170, $p \leqq 0.05$

7.2 Results of randomized block design two-way ANOVA (position, normalized determination coefficients)

We show full results of randomized block design two-way ANOVA on normalized determination coefficients for reconstructing time series of positional data (Tables S62 – S70).

Table S62. ANOVA summary table (position; normalized determination coefficients: R^2)

Source	Sum of Squares	Degree of Freedom	Mean Square	F-Value	Prob > F
Subject (S)	1.8948	n-1=5	0.3790		
Training task (A)					
Training (A)	6.2410	p-1=4	1.5603	5.0318*	0.0057
Error (A×S)	6.2015	(p-1)(n-1)=20	0.3101		
Test task (B)					
Test (B)	2.0456	q-1=4	0.5114	2.6549	0.0632
Error (B×S)	3.8525	(q-1)(n-1)=20	0.1926		
Interaction effect					
Interaction (A×B)	11.9057	(p-1)(q-1)=3	0.7441	3.4097*	< 0.0001
Error (A×B×S)	17.4587	(p-1)(q-1)(n-1)=80	0.2182		
Total (T)	49.5997	npq-1=149			

Table S63. Multiple comparison (position; normalized determination coefficients: R^2)

Main effect (Tukey HSD)

Training task (A)	Test task (B)	Mean difference	Standard error	P-Value
Main effect A				
Control	Overt 0.5 Hz	-0.5044	0.1017	0.0167
Control	Overt 0.8 Hz	-0.4619	0.1017	0.0316
Covert 0.5 Hz	Overt 0.5 Hz	-0.4444	0.1017	0.0408

Table S64. Simple main effect test (position; normalized determination coefficients: R^2)

Interaction Effects

Simple main effect		Sum of Squares	Degree of Freedom	Mean Square	F-Value	Prob > F
A(Control)		6.5093	4	1.6273	6.8779*	< 0.001
A(Covert 0.5 Hz)		4.8536	4	1.2134	5.1284*	0.0008
A(Covert 0.8 Hz)		3.9631	4	0.9908	4.1875*	0.0035
A(Overt 0.5 Hz)		1.0402	4	0.2600	1.0991	0.3613
A(Overt 0.8 Hz)		1.7805	4	0.4451	1.8814	0.1196
Error	$MS_{\text{pool(A)}}$		100	0.2366		
B(Control)		0.6207	4	0.1552	0.7281	0.5748
B(Covert 0.5 Hz)		1.8846	4	0.4711	2.2108	0.0732
B(Covert 0.8 Hz)		2.8884	4	0.7221	3.3884*	0.0121
B(Overt 0.5 Hz)		3.9822	4	0.9955	4.6715*	0.0017
B(Overt 0.8 Hz)		4.5754	4	1.1439	5.3674*	0.0005
Error	$MS_{\text{pool(B)}}$		100	0.2131		

* $p \leqq 0.05$

Table S65. Multiple comparison for the simple main effect test

[position; normalized determination coefficients: R^2; Training (A) - Test (Control)]

A(Control)

		Training (A)				
		Control	Covert 0.5 Hz	Covert 0.8 Hz	Overt 0.5 Hz	Overt 0.8 Hz
Training (A)	Test (B)	Test (B): Control				
Control	Control	–	0.1111	0.7556	0.9365*	0.9537*
Covert 0.5 Hz		–	–	0.8667*	1.0475*	1.0648*
Covert 0.8 Hz		–	–	–	0.1808	0.1981
Overt 0.5 Hz		–	–	–	–	0.0173
Overt 0.8 Hz		–	–	–	–	–

HSD = 0.7986, $p \leqq 0.05$

Table S66. Multiple comparison for the simple main effect test

[position; normalized determination coefficients: R^2; Training (A) - Test (Covert 0.5 Hz)]

A(Covert 0.5 Hz)

		Training (A)				
		Control	Covert 0.5 Hz	Covert 0.8 Hz	Overt 0.5 Hz	Overt 0.8 Hz
Training (A)	Test (B)	Test (B): Covert 0.5 Hz				
Control	Covert 0.5 Hz	–	0.1558	0.5903	0.7698	0.8088*
Covert 0.5 Hz		–	–	0.7461	0.9256*	0.9646*
Covert 0.8 Hz		–	–	–	0.1795	0.2185
Overt 0.5 Hz		–	–	–	–	0.0390
Overt 0.8 Hz		–	–	–	–	–

$HSD = 0.7986, p \leqq 0.05$

Table S67. Multiple comparison for the simple main effect test

[position; normalized determination coefficients: R^2; Training (A) - Test (Covert 0.8 Hz)]

A(Covert 0.8 Hz)

		Training (A)				
		Control	Covert 0.5 Hz	Covert 0.8 Hz	Overt 0.5 Hz	Overt 0.8 Hz
Training (A)	Test (B)	Test (B): Covert 0.8 Hz				
Control	Covert 0.8 Hz	–	0.1003	0.3975	0.5431	0.5087
Covert 0.5 Hz		–	–	0.2972	0.6435	0.6091
Covert 0.8 Hz		–	–	–	0.9407*	0.9063*
Overt 0.5 Hz		–	–	–	–	0.0344
Overt 0.8 Hz		–	–	–	–	–

$HSD = 0.7986, p \leqq 0.05$

Table S68. Multiple comparison for the simple main effect test

[position; normalized determination coefficients: R^2; Training (Covert 0.8 Hz) - Test (B)]

B(Covert 0.8 Hz)

		Training (A): Covert 0.8 Hz				
		Test (B)				
Training (A)	Test (B)	Control	Covert 0.5 Hz	Covert 0.8 Hz	Overt 0.5 Hz	Overt 0.8 Hz
Covert 0.8 Hz	Control	–	0.0096	0.7556*	0.2342	0.6124
	Covert 0.5 Hz	–	–	0.7461	0.2246	0.6028
	Covert 0.8 Hz	–	–	–	0.5215	0.1432
	Overt 0.5 Hz	–	–	–	–	0.3782
	Overt 0.8 Hz	–	–	–	–	–

$HSD = 0.7536, p \leqq 0.05$

Table S69. Multiple comparison for the simple main effect test

[position; normalized determination coefficients: R^2; Training (Overt 0.5 Hz) - Test (B)]

B(Overt 0.5 Hz)

		Training (A): Overt 0.5 Hz				
		Test (B)				
Training (A)	Test (B)	Control	Covert 0.5 Hz	Covert 0.8 Hz	Overt 0.5 Hz	Overt 0.8 Hz
Overt 0.5 Hz	Control	–	0.0109	0.0042	0.9365*	0.3417
	Covert 0.5 Hz	–	–	0.0151	0.9256*	0.3308
	Covert 0.8 Hz	–	–	–	0.9407*	0.3459
	Overt 0.5 Hz	–	–	–	–	0.5948
	Overt 0.8 Hz	–	–	–	–	–

HSD = 0.7536, $p \leqq 0.05$

Table S70. Multiple comparison for the simple main effect test

[position; normalized determination coefficients: R^2; Training (Overt 0.8 Hz) - Test (B)]

B(Overt 0.8 Hz)

		Training (A): Overt 0.8 Hz				
		Test (B)				
Training (A)	Test (B)	Control	Covert 0.5 Hz	Covert 0.8 Hz	Overt 0.5 Hz	Overt 0.8 Hz
Overt 0.8 Hz	Control	–	0.0108	0.0475	0.5937	0.9537*
	Covert 0.5 Hz	–	–	0.0583	0.6045	0.9646*
	Covert 0.8 Hz	–	–	–	0.5462	0.9063*
	Overt 0.5 Hz	–	–	–	–	0.3601
	Overt 0.8 Hz	–	–	–	–	–

HSD = 0.7536, $p \leqq 0.05$

7.3 Results of randomized block design two-way ANOVA (velocity, normalized correlation coefficients)

We show full results of randomized block design two-way ANOVA on normalized correlation coefficients for reconstructing time series of velocity data (Tables S71 – S82).

Table S71. ANOVA summary table (velocity; normalized correlation coefficients: *r*)

Source	Sum of Squares	Degree of Freedom	Mean Square	*F*-Value	Prob > *F*
Subject (*S*)	0.4504	*n*-1=5	0.0901		
Training task (A)					
Training (*A*)	0.3067	*p*-1=4	0.0767	1.9708	0.1380
Error (*A*×*S*)	0.7782	(*p*-1)(*n*-1)=20	0.0389		
Test task (B)					
Test (*B*)	0.0347	*q*-1=4	0.0087	0.1494	0.9611
Error (*B*×*S*)	1.1599	(*q*-1)(*n*-1)=20	0.0580		
Interaction effect					
Interaction (*A*×*B*)	10.2963	(*p*-1)(*q*-1)=3	0.6435	19.3492*	< 0.0001
Error (*A*×*B*×*S*)	2.6607	(*p*-1)(*q*-1)(*n*-1)=80	0.0333		
Total (*T*)	15.6868	*npq*-1=149			

* $p \leqq 0.05$

Table S72. Simple main effect test (velocity; normalized correlation coefficients: *r*)

Interaction Effects

Simple main effect	Sum of Squares	Degree of Freedom	Mean Square	*F*-Value	Prob > F
A(Control)	2.4935	4	0.6234	18.1279*	< 0.0001
A(Covert 0.5 Hz)	2.4197	4	0.6049	17.5911*	< 0.0001
A(Covert 0.8 Hz)	1.4316	4	0.3579	10.4078*	< 0.0001
A(Overt 0.5 Hz)	1.4041	4	0.3510	10.2080*	< 0.0001
A(Overt 0.8 Hz)	2.8540	4	0.7135	20.7488*	< 0.0001
Error	$MS_{\text{pool(A)}}$	100	0.0344		
B(Control)	1.8387	4	0.4581	11.9909*	< 0.0001
B(Covert 0.5 Hz)	2.8662	4	0.7307	19.1254*	< 0.0001
B(Covert 0.8 Hz)	0.9027	4	0.2235	5.8501*	0.0003
B(Overt 0.5 Hz)	2.3563	4	0.5901	15.4464*	< 0.0001
B(Overt 0.8 Hz)	2.3240	4	0.5803	15.1889*	< 0.0001
Error	$MS_{\text{pool(B)}}$	100	0.0382		

* $p \leqq 0.05$

Table S73. Multiple comparison for the simple main effect test
[velocity; normalized correlation coefficients: *r*; Training (A) - Test (Control)]

A(Control)

		Training (A)				
		Control	Covert 0.5 Hz	Covert 0.8 Hz	Overt 0.5 Hz	Overt 0.8 Hz
Training (A)	Test (B)	Test (B): Control				
Control	Control	–	0.0490	0.3994*	0.6381*	0.6924*
Covert 0.5 Hz		–	–	0.3504*	0.5890*	0.6434*
Covert 0.8 Hz		–	–	–	0.2386	0.2930
Overt 0.5 Hz		–	–	–	–	0.0544
Overt 0.8 Hz		–	–	–	–	–

HSD = 0.3037, $p \leqq 0.05$

Table S74. Multiple comparison for the simple main effect test
[velocity; normalized correlation coefficients: *r*; Training (A) - Test (Covert 0.5 Hz)]

A(Covert 0.5 Hz)

		Training (A)				
		Control	Covert 0.5 Hz	Covert 0.8 Hz	Overt 0.5 Hz	Overt 0.8 Hz
Training (A)	Test (B)	Test (B): Covert 0.5 Hz				
Control	Covert 0.5 Hz	–	0.0329	0.3871*	0.5469*	0.6672*
Covert 0.5 Hz		–	–	0.4200*	0.5798*	0.7001*
Covert 0.8 Hz		–	–	–	0.1598	0.2801
Overt 0.5 Hz		–	–	–	–	0.1203
Overt 0.8 Hz		–	–	–	–	–

HSD = 0.3037, $p \leqq 0.05$

Table S75. Multiple comparison for the simple main effect test
[velocity; normalized correlation coefficients: *r*; Training (A) - Test (Covert 0.8 Hz)]

A(Covert 0.8 Hz)

		Training (A)				
		Control	Covert 0.5 Hz	Covert 0.8 Hz	Overt 0.5 Hz	Overt 0.8 Hz
Training (A)	Test (B)	Test (B): Covert 0.8 Hz				
Control	Covert 0.8 Hz	–	0.0039	0.2886	0.3611*	0.2007
Covert 0.5 Hz		–	–	0.2925	0.3572*	0.1969
Covert 0.8 Hz		–	–	–	0.6496*	0.4893*
Overt 0.5 Hz		–	–	–	–	0.1603
Overt 0.8 Hz		–	–	–	–	–

HSD = 0.3037, $p \leqq 0.05$

Table S76. Multiple comparison for the simple main effect test

[velocity; normalized correlation coefficients: *r*; Training (A) - Test (Overt 0.5 Hz)]

A(Overt 0.5 Hz)

		Training (A)				
		Control	Covert 0.5 Hz	Covert 0.8 Hz	Overt 0.5 Hz	Overt 0.8 Hz
Training (A)	Test (B)	Test (B): Overt 0.5 Hz				
Control	Overt 0.5 Hz	–	0.0786	0.0057	0.4872*	0.2977
Covert 0.5 Hz		–	–	0.0729	0.5658*	0.3763*
Covert 0.8 Hz		–	–	–	0.4929*	0.3034
Overt 0.5 Hz		–	–	–	–	0.1894
Overt 0.8 Hz		–	–	–	–	–

HSD = 0.3037, $p \leqq 0.05$

Table S77. Multiple comparison for the simple main effect test

[velocity; normalized correlation coefficients: *r*; Training (A) - Test (Overt 0.8 Hz)]

A(Overt 0.8 Hz)

		Training (A)				
		Control	Covert 0.5 Hz	Covert 0.8 Hz	Overt 0.5 Hz	Overt 0.8 Hz
Training (A)	Test (B)	Test (B): Overt 0.8 Hz				
Control	Overt 0.8 Hz	–	0.1924	0.2739	0.5178*	0.6311*
Covert 0.5 Hz		–	–	0.4663*	0.7102*	0.8235*
Covert 0.8 Hz		–	–	–	0.2439	0.3572*
Overt 0.5 Hz		–	–	–	–	0.1133
Overt 0.8 Hz		–	–	–	–	–

HSD = 0.3037, $p \leqq 0.05$

Table S78. Multiple comparison for the simple main effect test

[velocity; normalized correlation coefficients: *r*; Training (Control) - Test (B)]

B(Control)

		Training (A): Control				
		Test (B)				
Training (A)	Test (B)	Control	Covert 0.5 Hz	Covert 0.8 Hz	Overt 0.5 Hz	Overt 0.8 Hz
Control	Control	–	0.0329	0.2886	0.4872*	0.6311*
	Covert 0.5 Hz	–	–	0.2557	0.4557*	0.5982*
	Covert 0.8 Hz	–	–	–	0.1986	0.3425*
	Overt 0.5 Hz	–	–	–	–	0.1439
	Overt 0.8 Hz	–	–	–	–	–

HSD = 0.3219, $p \leqq 0.05$

Table S79. Multiple comparison for the simple main effect test

[velocity; normalized correlation coefficients: *r*; Training (Covert 0.5 Hz) - Test (B)]

B(Covert 0.5 Hz)

		Training (A): Covert 0.5 Hz				
		Test (B)				
Training (A)	Test (B)	Control	Covert 0.5 Hz	Covert 0.8 Hz	Overt 0.5 Hz	Overt 0.8 Hz
Covert 0.5 Hz	Control	–	0.0490	0.2435	0.5168*	0.7745*
	Covert 0.5 Hz	–	–	0.2925	0.5658*	0.8235*
	Covert 0.8 Hz	–	–	–	0.2733	0.5311*
	Overt 0.5 Hz	–	–	–	–	0.2577
	Overt 0.8 Hz	–	–	–	–	–

HSD = 0.3219, $p \leqq 0.05$

Table S80. Multiple comparison for the simple main effect test

[velocity; normalized correlation coefficients: *r*; Training (Covert 0.8 Hz) - Test (B)]

B(Covert 0.8 Hz)

		Training (A): Covert 0.8 Hz				
		Test (B)				
Training (A)	Test (B)	Control	Covert 0.5 Hz	Covert 0.8 Hz	Overt 0.5 Hz	Overt 0.8 Hz
Covert 0.8 Hz	Control	–	0.0205	0.3994*	0.0934	0.0422
	Covert 0.5 Hz	–	–	0.4200*	0.0729	0.0628
	Covert 0.8 Hz	–	–	–	0.4929*	0.3572*
	Overt 0.5 Hz	–	–	–	–	0.1357
	Overt 0.8 Hz	–	–	–	–	–

HSD = 0.3219, $p \leqq 0.05$

Table S81. Multiple comparison for the simple main effect test

[velocity; normalized correlation coefficients: *r*; Training (Overt 0.5 Hz) - Test (B)]

B(Overt 0.5 Hz)

		Training (A): Overt 0.5 Hz				
		Test (B)				
Training (A)	Test (B)	Control	Covert 0.5 Hz	Covert 0.8 Hz	Overt 0.5 Hz	Overt 0.8 Hz
Overt 0.5 Hz	Control	–	0.0583	0.0116	0.6381*	0.5247*
	Covert 0.5 Hz	–	–	0.0699	0.5798*	0.4665*
	Covert 0.8 Hz	–	–	–	0.6496*	0.5363*
	Overt 0.5 Hz	–	–	–	–	0.1133
	Overt 0.8 Hz	–	–	–	–	–

HSD = 0.3219, $p \leqq 0.05$

Table S82. Multiple comparison for the simple main effect test
[velocity; normalized correlation coefficients: *r*; Training (Overt 0.8 Hz) - Test (B)]

B(Overt 0.8 Hz)

		Training (A): Overt 0.8 Hz				
		Test (B)				
Training (A)	Test (B)	Control	Covert 0.5 Hz	Covert 0.8 Hz	Overt 0.5 Hz	Overt 0.8 Hz
Overt 0.8 Hz	Control	–	0.0077	0.2031	0.5030*	0.6924*
	Covert 0.5 Hz	–	–	0.2108	0.5107*	0.7001*
	Covert 0.8 Hz	–	–	–	0.2999	0.4893*
	Overt 0.5 Hz	–	–	–	–	0.1894
	Overt 0.8 Hz	–	–	–	–	–

HSD = 0.3219, $p \leqq 0.05$

7.4 Results of randomized block design two-way ANOVA (velocity, normalized determination coefficients)

We show full results of randomized block design two-way ANOVA on normalized determination coefficients for reconstructing time series of velocity data (Tables S83 – S93).

Table S83. ANOVA summary table (velocity; normalized determination coefficients: R^2)

Source	Sum of Squares	Degree of Freedom	Mean Square	*F*-Value	Prob > *F*
Subject (*S*)	0.4462	*n*-1=5	0.0892		
Training task (A)					
Training (*A*)	2.4927	*p*-1=4	0.6232	2.2029	0.1054
Error (*A*×*S*)	5.6579	(*p*-1)(*n*-1)=20	0.2829		
Test task (B)					
Test (*B*)	0.4165	*q*-1=4	0.1041	0.8815	0.4927
Error (*B*×*S*)	2.3623	(*q*-1)(*n*-1)=20	0.1181		
Interaction effect					
Interaction (*A*×*B*)	23.6722	(*p*-1)(*q*-1)=3	1.4795	5.5868*	< 0.0001
Error (*A*×*B*×*S*)	21.1859	(*p*-1)(*q*-1)(*n*-1)=80	0.2648		
Total (*T*)	56.2337	*npq*-1=149			

* $p \leqq 0.05$

Table S84. Simple main effect test (velocity; normalized determination coefficients: R^2)

Interaction Effects

Simple main effect	Sum of Squares	Degree of Freedom	Mean Square	F-Value	Prob > F
A(Control)	5.3676	4	1.3419	4.9989*	0.0010
A(Covert 0.5 Hz)	4.5929	4	1.1482	4.2775*	0.0031
A(Covert 0.8 Hz)	3.5247	4	0.8812	3.2826*	0.0142
A(Overt 0.5 Hz)	3.4368	4	0.8592	3.2008*	0.0162
A(Overt 0.8 Hz)	9.2429	4	2.3107	8.6081*	< 0.0001
Error	$MS_{\text{pool(A)}}$	100	0.2684		
B(Control)	1.5506	4	0.3877	1.6462	0.1686
B(Covert 0.5 Hz)	11.5506	4	2.9802	12.6557*	< 0.0001
B(Covert 0.8 Hz)	1.1718	4	0.2930	1.2441	0.2972
B(Overt 0.5 Hz)	4.9936	4	1.2484	5.3014*	0.0006
B(Overt 0.8 Hz)	4.4520	4	1.1130	4.7264*	0.0016
Error	$MS_{\text{pool(B)}}$	100	0.2355		

* $p \leqq 0.05$

Table S85. Multiple comparison for the simple main effect test [velocity; normalized determination coefficients: R^2; Training (A) – Test (Control)]

A(Control)

		Training (A)				
		Control	Covert 0.5 Hz	Covert 0.8 Hz	Overt 0.5 Hz	Overt 0.8 Hz
Training (A)	Test (B)	Test (B): Control				
Control	Control	–	0.0224	0.4716	0.9325*	0.9364*
Covert 0.5 Hz		–	–	0.4940	0.9549*	0.9588*
Covert 0.8 Hz		–	–	–	0.4609	0.4648
Overt 0.5 Hz		–	–	–	–	0.0039
Overt 0.8 Hz		–	–	–	–	–

$HSD = 0.8476$, $p \leqq 0.05$

Table S86. Multiple comparison for the simple main effect test

[velocity; normalized determination coefficients: R^2; Training (A) - Test (Covert 0.5 Hz)]

A(Covert 0.5 Hz)

		Training (A)				
		Control	Covert 0.5 Hz	Covert 0.8 Hz	Overt 0.5 Hz	Overt 0.8 Hz
Training (A)	Test (B)	Test (B): Covert 0.5 Hz				
Control	Covert 0.5 Hz	–	0.1060	0.3755	0.8017	0.8350
Covert 0.5 Hz		–	–	0.4815	0.9077*	0.9410*
Covert 0.8 Hz		–	–	–	0.4262	0.4596
Overt 0.5 Hz		–	–	–	–	0.0333
Overt 0.8 Hz		–	–	–	–	–

$HSD = 0.8476, p \leqq 0.05$

Table S87. Multiple comparison for the simple main effect test

[velocity; normalized determination coefficients: R^2; Training (A) - Test (Covert 0.8 Hz)]

A(Covert 0.8 Hz)

		Training (A)				
		Control	Covert 0.5 Hz	Covert 0.8 Hz	Overt 0.5 Hz	Overt 0.8 Hz
Training (A)	Test (B)	Test (B): Covert 0.8 Hz				
Control	Covert 0.8 Hz	–	0.0051	0.4503	0.4799	0.4456
Covert 0.5 Hz		–	–	0.4453	0.4849	0.4506
Covert 0.8 Hz		–	–	–	0.9302*	0.8959*
Overt 0.5 Hz		–	–	–	–	0.0343
Overt 0.8 Hz		–	–	–	–	–

$HSD = 0.8476, p \leqq 0.05$

Table S88. Multiple comparison for the simple main effect test

[velocity; normalized determination coefficients: R^2; Training (A) - Test (Overt 0.5 Hz)]

A(Overt 0.5 Hz)

		Training (A)				
		Control	Covert 0.5 Hz	Covert 0.8 Hz	Overt 0.5 Hz	Overt 0.8 Hz
Training (A)	Test (B)	Test (B): Overt 0.5 Hz				
Control	Overt 0.5 Hz	–	0.6471	0.0787	0.3986	0.0584
Covert 0.5 Hz		–	–	0.5684	1.0457*	0.7056
Covert 0.8 Hz		–	–	–	0.4773	0.1372
Overt 0.5 Hz		–	–	–	–	0.3401
Overt 0.8 Hz		–	–	–	–	–

$HSD = 0.8476, p \leqq 0.05$

Table S89. Multiple comparison for the simple main effect test

[velocity; normalized determination coefficients: R^2; Training (A) - Test (Overt 0.8 Hz)]

A(Overt 0.8 Hz)

		Training (A)				
		Control	Covert 0.5 Hz	Covert 0.8 Hz	Overt 0.5 Hz	Overt 0.8 Hz
Training (A)	Test (B)	Test (B): Overt 0.8 Hz				
Control	Overt 0.8 Hz	–	0.9936*	0.0819	0.4131	0.6154
Covert 0.5 Hz		–	–	1.0754*	1.4066*	1.6090*
Covert 0.8 Hz		–	–	–	0.3312	0.5335
Overt 0.5 Hz		–	–	–	–	0.2023
Overt 0.8 Hz		–	–	–	–	–

HSD = 0.8476, p ≦ 0.05

Table S90. Multiple comparison for the simple main effect test

[velocity; normalized determination coefficients: R^2; Training (Covert 0.5 Hz) - Test (B)]

B(Covert 0.5 Hz)

		Training (A): Control				
		Test (B)				
Training (A)	Test (B)	Control	Covert 0.5 Hz	Covert 0.8 Hz	Overt 0.5 Hz	Overt 0.8 Hz
Control	Control	–	0.0224	0.4677	1.0681*	1.6314*
	Covert 0.5 Hz	–	–	0.4453	1.0457*	1.6090*
	Covert 0.8 Hz	–	–	–	0.6004	1.1637*
	Overt 0.5 Hz	–	–	–	–	0.5632
	Overt 0.8 Hz	–	–	–	–	–

HSD = 0.7876, p ≦ 0.05

Table S91. Multiple comparison for the simple main effect test

[velocity; normalized determination coefficients: R^2; Training (Overt 0.5 Hz) - Test (B)]

B(Overt 0.5 Hz)

		Training (A): Covert 0.5 Hz				
		Test (B)				
Training (A)	Test (B)	Control	Covert 0.5 Hz	Covert 0.8 Hz	Overt 0.5 Hz	Overt 0.8 Hz
Covert 0.5 Hz	Control	–	0.0535	0.4236	0.8244*	1.2193*
	Covert 0.5 Hz	–	–	0.4771	0.8779*	1.2728*
	Covert 0.8 Hz	–	–	–	0.4008	0.7957*
	Overt 0.5 Hz	–	–	–	–	0.3949
	Overt 0.8 Hz	–	–	–	–	–

HSD = 0.7876, p ≦ 0.05

Table S92. Multiple comparison for the simple main effect test

[velocity; normalized determination coefficients: R^2; Training (Overt 0.5 Hz) - Test (B)]

B(Overt 0.5 Hz)

		Training (A): Overt 0.5 Hz				
		Test (B)				
Training (A)	Test (B)	Control	Covert 0.5 Hz	Covert 0.8 Hz	Overt 0.5 Hz	Overt 0.8 Hz
Overt 0.5 Hz	Control	–	0.0248	0.0023	0.9325*	0.7302
	Covert 0.5 Hz	–	–	0.0225	0.9077*	0.7054
	Covert 0.8 Hz	–	–	–	0.9302*	0.7279
	Overt 0.5 Hz	–	–	–	–	0.2023
	Overt 0.8 Hz	–	–	–	–	–

$HSD = 0.7876, p \leqq 0.05$

Table S93. Multiple comparison for the simple main effect test

[velocity; normalized determination coefficients: R^2; Training (Overt 0.8 Hz) - Test (B)]

B(Overt 0.8 Hz)

		Training (A): Overt 0.8 Hz				
		Test (B)				
Training (A)	Test (B)	Control	Covert 0.5 Hz	Covert 0.8 Hz	Overt 0.5 Hz	Overt 0.8 Hz
Overt 0.8 Hz	Control	–	0.0046	0.0405	0.5963	0.9364*
	Covert 0.5 Hz	–	–	0.0451	0.6009	0.9410*
	Covert 0.8 Hz	–	–	–	0.5558	0.8959*
	Overt 0.5 Hz	–	–	–	–	0.3401
	Overt 0.8 Hz	–	–	–	–	–

$HSD = 0.7876, p \leqq 0.05$

8 Time lag between cortical currents and target motion for SLiR

We reconstructed target kinetics from cortical currents on each cortical region under the overt condition by varying the time lag between cortical currents and target kinetics. After that, we calculated correlation coefficients between target kinetics and reconstructed data (Figure S17).

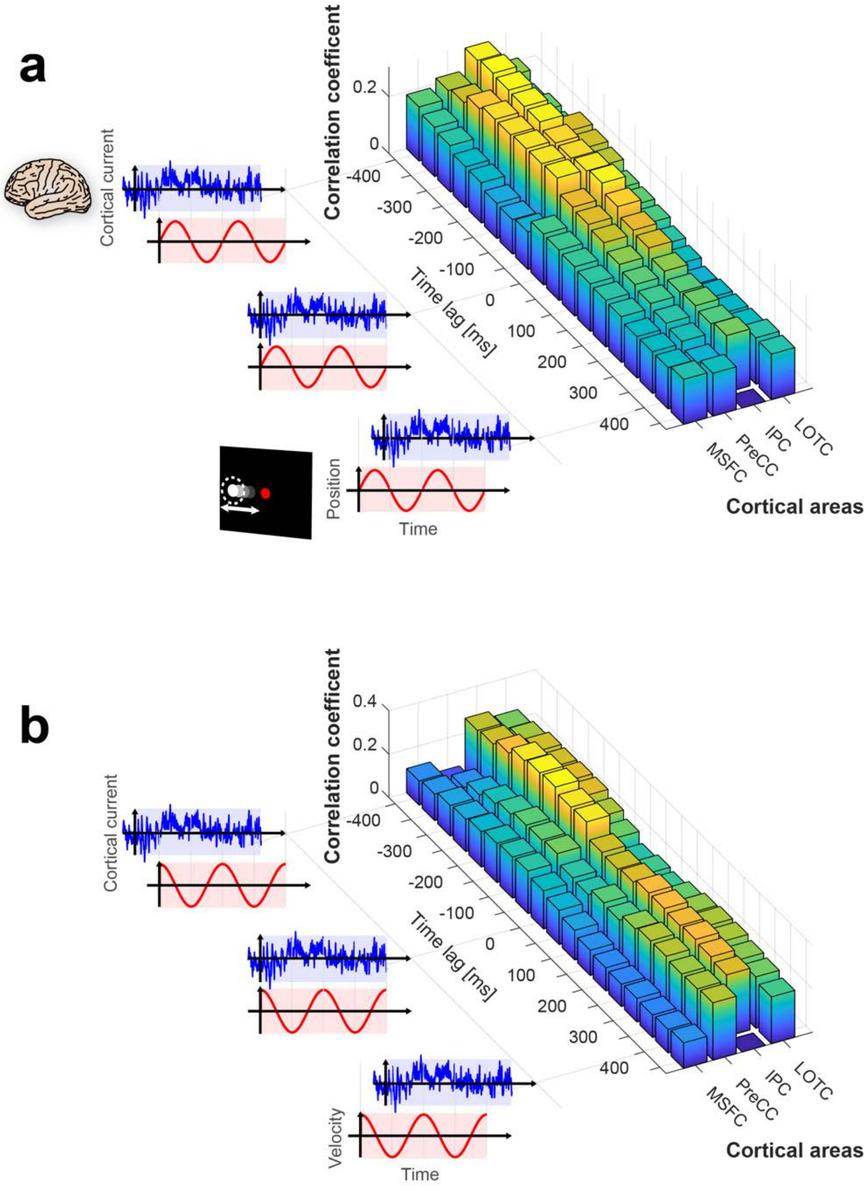

Figure S17. Effects of time lag between cortical currents and target kinetics (Subj.5; overt condition). (**a**) Correlation coefficients between target positions and reconstructed data. (**b**) Correlation coefficients between target velocities and reconstructed data.

9 Preliminary training

Before the main experiment began, we conducted a preliminary experiment (4 tasks [covert0.5Hz/covert0.8Hz/overt0.5Hz/overt0.8Hz] × 40 trials = 160 trials) without MEG observation on a different day for all participants, in order to familiarize them with the covert/overt visual pursuit tasks. In this preliminary experiment, we monitored eye movements on-line via Monitor using the EyeLink-II system (SR-Research Ltd., Mississauga, Ontario, Canada). We also gave verbal feedback if required. Finally, all participants were able to control their eye movements according to the covert/overt visual pursuit tasks.

References

1. Belopolsky A.V. & Theeuwes J. When are attention and saccade preparation dissociated? *Psychol Sci.* **20**(11), 1340-7 (2009).
2. Casteau, S & Smith, D. Associations and Dissociations between Oculomotor Readiness and Covert Attention. *Vision*. **2**, 17 (2019).
3. Benjamini, Y. & Hochberg, Y. Controlling the false discovery rate: a practical and powerful approach to multiple testing. *J. Roy. Statist. Soc. Ser. B* **57**, 289-300 (1995).
